# Synthesis of Mono‐, Di‐, Tri‐, and Tetra‐cationic Pyridinium and Vinylpyridinium Modified [2.2]Paracyclophanes: Modular Receptors for Supramolecular Systems

**DOI:** 10.1002/open.202400024

**Published:** 2024-03-12

**Authors:** Yichuan Wang, Yuting Li, Olaf Fuhr, Martin Nieger, Zahid Hassan, Stefan Bräse

**Affiliations:** ^1^ Institute of Organic Chemistry (IOC) Karlsruhe Institute of Technology (KIT) Fritz-Haber-Weg 6 76131 Karlsruhe Germany; ^2^ Institute of Nanotechnology (INT) and Karlsruhe Nano Micro Facility (KNMFi) Karlsruhe Institute of Technology (KIT) Hermann-von-Helmholtz-Platz 1 76344 Eggenstein-Leopoldshafen Germany; ^3^ Department of Chemistry University of Helsinki P. O. Box 55 00014 University of Helsinki Finland; ^4^ Institute of Biological and Chemical Systems Functional Molecular Systems (IBCS-FMS) Karlsruhe Institute of Technology (KIT) Hermann-von-Helmholtz-Platz 1 76344 Eggenstein-Leopoldshafen Germany

**Keywords:** Cationic Pyridines, 3D Molecular Tectons, Supramolecular Interactions, Molecular receptors, Cross-coupling chemistry, [2.2]Paracyclophane

## Abstract

In this report, a new series of mono‐, di‐, tri‐, and tetra‐cationic pyridinium and vinyl pyridinium‐modified [2.2]paracyclophanes as useful molecular tectons for supramolecular systems are described. Regioselective functionalization at specific positions, followed by resolution step and successive transformations through Pd‐catalyzed Suzuki‐Miyaura and Mizoroki‐Heck cross‐coupling chemistry furnish a series of modular PCP scaffolds. In our proof‐of‐concept study, on *N*‐methylation, the PCPs bearing (cationic) pyridyl functionalities were demonstrated as useful molecular receptors in host‐guest supramolecular assays. The PCPs on grafting with light‐responsive azobenzene (−N=N−) functional core as side‐groups impart photosensitivity that can be remotely transformed on irradiation, offering photo‐controlled smart molecular functions. Furthermore, the symmetrical PCPs bearing bi‐, and tetra‐pyridyl functionalities at the peripheries have enormous potential to serve as ditopic and tetratopic 3D molecular tectons for engineering non‐covalent supramolecular assemblies with new structural and functional attributes.

## Graphical abstract

**Modular Scaffolds for Supramolecular Systems**: In this report, a new series of mono‐, di‐, tri‐, and tetra‐cationic pyridinium and vinylpyridinium modified [2.2]paracyclophanes (PCPs) is described. On *N*‐methylation, the 3D PCPs bearing (cationic) pyridyl and vinylpyridinium functionalities have been demonstrated as efficient molecular receptors for application in supramolecular systems. The PCPs on grafting with light‐responsive azobenzene (−N=N−) functional core as side‐groups impart photosensitivity that can be remotely transformed on irradiation, offering photo‐controlled smart molecular functions.

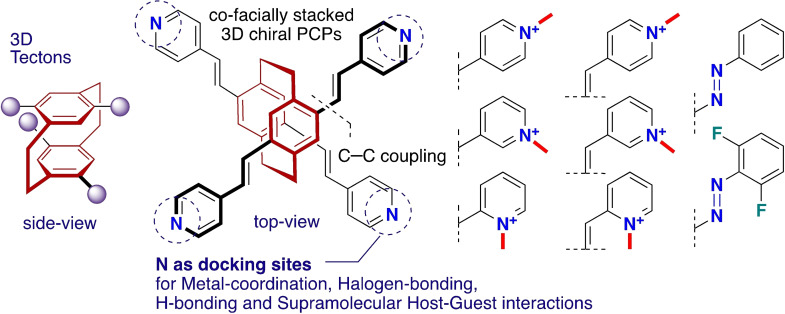

## Introduction

**Figure 1:**
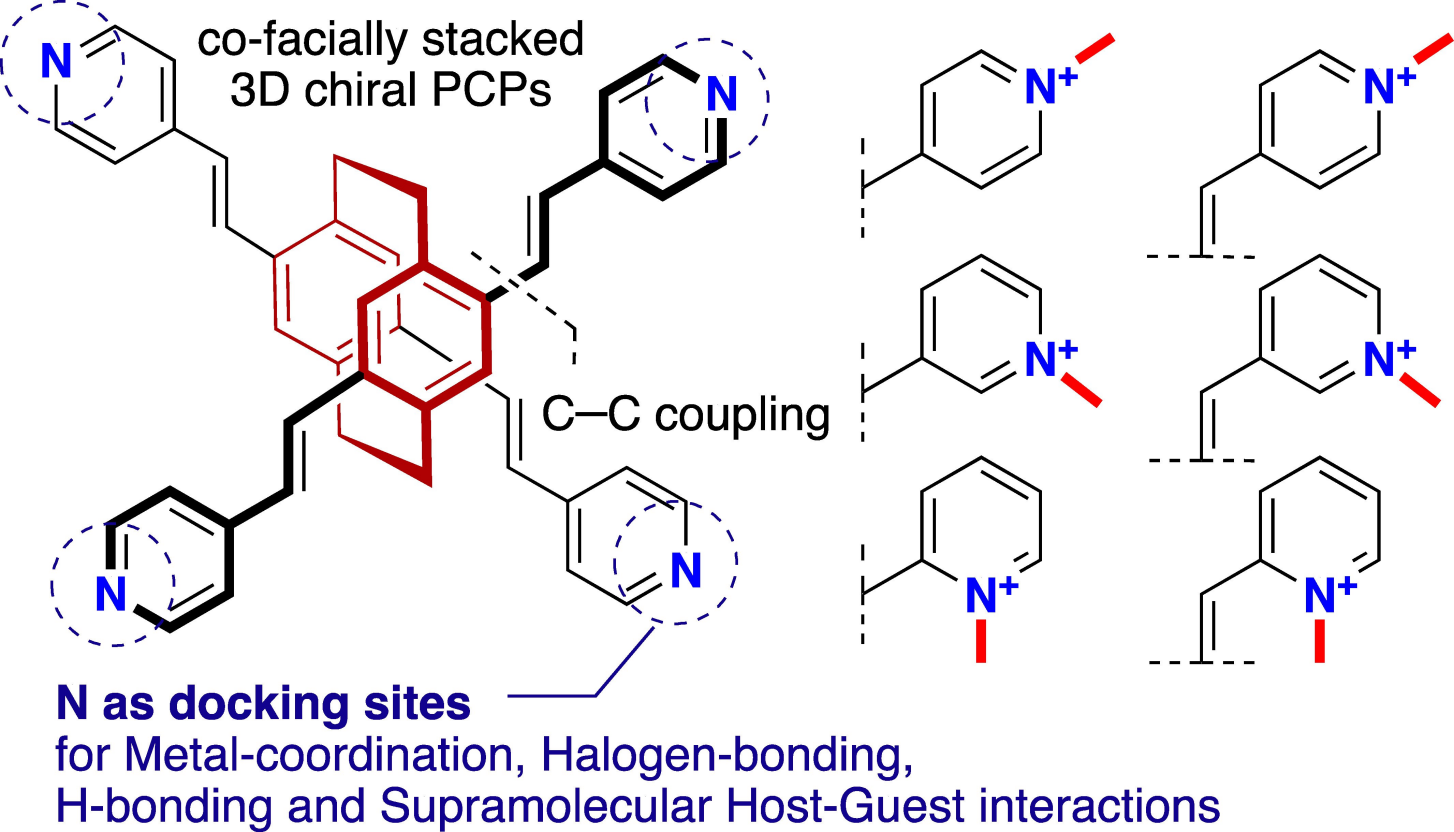
Design concept of pyridyl‐ and vinylpyridyl‐modified cationic PCPs as potential scaffolds for supramolecular systems.

[2.2]Paracyclophanes (PCPs) is a small stable co‐facially stacked pro‐chiral 3D scaffold consisting of two coplanar phenyl rings linked in a face‐to‐face orientation by two short ethylene bridges −[CH_2_]_2_− in their *para*‐positions that features unusual chemical characteristics caused by the transannular π–π electronic interactions of the benzene rings stacked nearby and exhibits unique stereochemical features (planar chirality) upon selective substitution.^[1]^ Rigidity, stability, planarity, conformational behaviors, chirality, π‐stacking, and electronic communication via through‐space and through‐bond pathways within the PCP are interesting molecular features/aspects for consideration as useful precursors in materials fabrication. PCPs have been largely investigated as planar chiral ligands in asymmetric catalysis which proved to be a useful toolbox for stereo‐controlled synthesis,^[2]^ π‐stacked conjugated polymers,^[3]^ optoelectronic materials,^[4]^ and technologically‐relevant polymer coatings (e. g., parylene) of industrial importance formed by chemical vapor deposition polymerization using PCP‐based precursors.[^5^, ^6^] Many of the functions and features of cyclophane‐based materials are the province of their molecular‐level chemistry. To serve a particular purpose, the optimal structure and functional design of the fundamental PCP i. e., chemically‐programmed core is of utmost relevance,^[7]^ as the selective substitution pattern on the benzene (phanes, or decks) and/or ethylene bridges heavily influences the nature of the PCP, and even minor changes can alter its properties significantly.^[8]^ For instance, the ingenious design of the chemically‐programmed PCP tectons (PCP appended with four (methoxyphenyl)ethynyl arms) in enantiopure form brings the innate stereochemical features (planar chirality) and facilitates vital non‐covalent interactions of C−H⋅⋅⋅π, C−H⋅⋅⋅O, and π–π interactions to direct conformational arrangements, which eventually lead to unprecedented stereocontrolled skeletal morphology of 3D microvessels.^[9]^ The 4,7,12,15‐tetracarboxamide‐susbtituted PCP generates supramolecular cyclophane stacks helically laced‐up by two transannular H‐bond strands where the chiral sense is dictated by the planar chirality of the PCP (*R*_p_ or *S*_p_).^[10]^ PCP backbone on functionalizing with coordination‐capable phenyl‐carboxylates moieties enables strong metal‐coordination in combination with inorganic metal‐nodes and thus forms diverse coordination‐driven structures featuring intriguing properties.^[11]^ PCPs bearing multi‐pyridyl end‐groups hold enormous potential from application perspectives in engineering diverse supramolecular systems as demonstrated for other non‐cyclophanyl scaffolds.^[12]^ PCP derivatives are particularly appealing, inheriting the innate planar chirality and layered structure of the PCP. In this contribution, we envision the mono‐, di‐, tri‐, and tetra‐cationic pyridinium and vinylpyridinium‐modified PCP scaffolds by palladium‐catalyzed cross‐coupling reactions. By employing the co‐facially stacked cyclophanyl‐derived constitutional isomers allow the installation of pyridinium and vinylpyridinium moieties in a distance‐defined and spatially‐oriented relation (Figure 1). Model PCPs bearing (cationic) pyridyl functional end‐groups have been demonstrated as efficient molecular receptors in host‐guest supramolecular assays.

## Results and Discussion

**Scheme 1:**
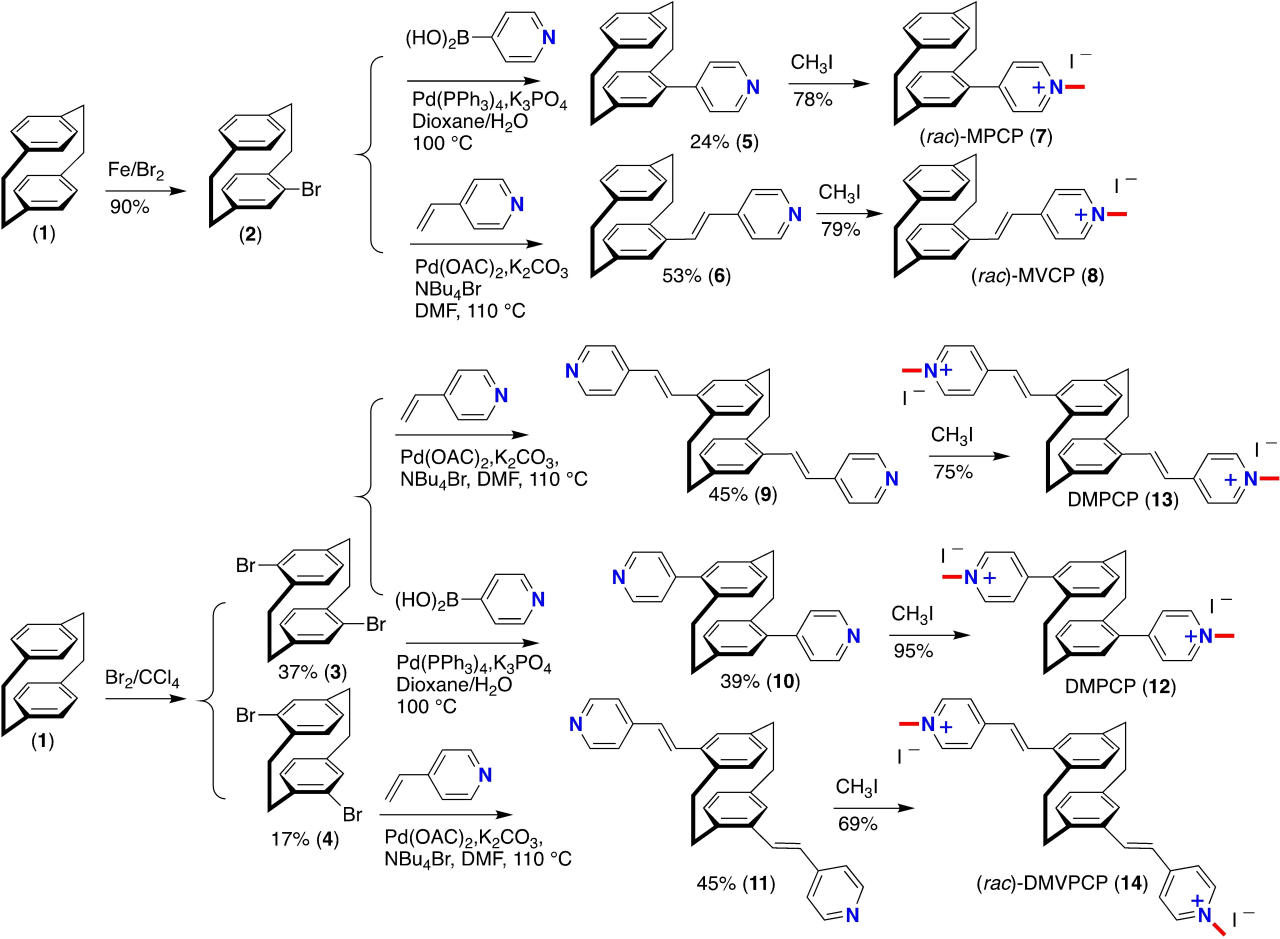
Synthesis of mono‐ **7** and differently‐functionalized di‐cationic pyridinium **12** and vinylpyridinium‐modified PCPs **(8, 13**, and **14)** prepared via Pd‐catalyzed Suzuki‐Miyaura and Mizoroki‐Heck cross‐coupling chemistry, followed by on *N*‐methylation.

We have a long‐standing interest in enlarging the chemical space around the PCP scaffold‐ensuring the holistic approach of designing functional molecules for functional materials with technologically relevant applications ranging from planar chiral ligands/catalysts and through‐space electronic communications to functional parylene coatings.^[13]^ We commenced our studies by regioselective functionalization of the PCP **1**, employing our previously established synthetic procedure^[14]^ to access differently‐substituted mono‐, and dibromo‐cyclophanyl products. Using carefully chosen synthesis routes and transformation steps, the PCP core allows different functional groups to be selectively positioned at either only one or both benzene rings. The mono‐ (**2**), and dibromo PCPs (both pseudo *para*‐PCP (**3**) and pseudo‐*meta*‐PCP (**4**) were subsequently transformed by Pd‐catalyzed Suzuki‐Miyaura cross‐coupling for the construction of the aryl‐aryl bonds and Mizoroki‐Heck cross‐coupling chemistry for the formation of carbon‐carbon double bonds as shown in Scheme 1. For disubstitution on one phenyl ring of the PCP, the conventional prefixes of *ortho*‐, *meta*‐, and *para*‐ are used, while disubstitution on two transannularly adjacent positions on the benzene rings gives rise to pseudo‐*geminal*, pseudo‐*ortho*, pseudo‐*meta*, and pseudo‐*para* prefixes to specify *ortho*‐, *meta*‐, and *para*‐ relationships displaced from the usual homoannular into a transannular context. To incorporate the pyridine or vinylpyridine at the peripheries, the corresponding boronic acids or vinylpyridines were used as coupling partners. In this particular set‐up, the Suzuki‐Miyaura cross‐coupling was performed using palladium‐tetrakis(triphenylphosphine) as a catalyst source, potassium phosphate as a base in a biphasic solvent mixture of 1,4‐dioxane/water (2 : 1 v/v) at 100 °C for 16 h. To couple vinylpyridiyl component, Mizoroki‐Heck cross‐coupling was performed using palladium acetate as a catalyst in combination with K_2_CO_3_ in DMF at 100 °C for 18 hours. On *N*‐methylation by using methyl iodide in acetonitrile, mono‐, **7**, and di‐cationic pyridinium **12**, as well as vinylpyridinium‐modified PCPs of **8**, **13**, and **14** were obtained in high yields. The reactions were performed at 40 °C for 16 h and were protected from direct light (see the Supporting Information). Tetrabromo‐substituted PCPs i. e., 4,7,12,15‐tetrabromo‐PCP **15** and 4,5,12,13‐tetrabromo‐PCP **16** were prepared by slowly adding PCP to a mixture of liquid bromine and catalytic iodine. The reaction was stirred for 7 days at room temperature in a flask wrapped with aluminum foil to exclude light.^[15]^ This led to the two structural isomers of homo‐symmetrically tetrasubstituted products **15** and **16** in 27 % and 21 % yield respectively after purification by recrystallization in dichloromethane. Precisely pre‐designed building blocks, especially the design of angles and points of extension at the building blocks′ peripheries are considered crucial. The tetra‐substituted PCP grafted with stilbenoid dimer‐type components in varying spatial arrangements have been studied to examine the influence on the optical properties of the chromophores components as a function of their spatial relationship compared to the non‐strained non‐cyclophanyl stilbenoid dimer‐type analogues.^[16]^

**Scheme 2:**
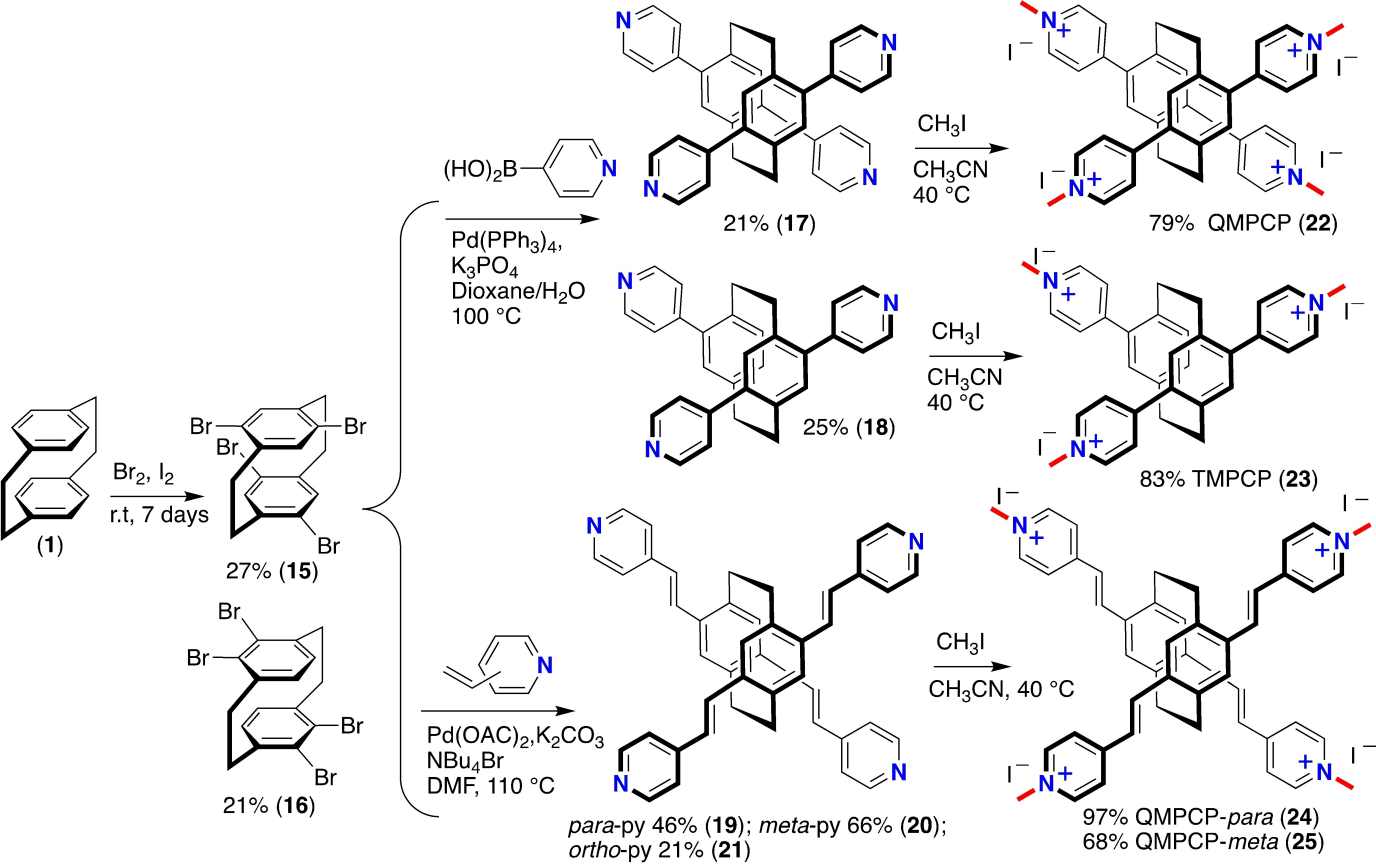
Synthesis of tetra‐cationic pyridinium **22**, tri‐ **23** and vinylpyridinium‐modified PCPs (**24**, **25**) prepared *via* Pd‐catalyzed Suzuki‐Miyaura and Mizoroki‐Heck cross‐coupling reaction employing **15**, followed by on *N*‐methylation.

During our investigations for grafting tetra‐cationic pyridinium and vinylpyridinium‐modified PCPs as model receptors, product **15** was transformed by tetra‐fold Pd‐catalyzed Suzuki‐Miyaura and Mizoroki‐Heck cross‐coupling chemistry employing different pyridine‐4‐boronic acid or different vinylpyrdiyl cross‐coupling components to afford the PCP‐scaffolds with varying spatial 3D arrangements. Pd‐catalyzed Suzuki‐Miyaura afforded tetra‐pyridyl substituted PCP (**17**) in 21 % yield. During this reaction, a side‐product of tri‐pyridyl substituted PCP (**18**) was also isolated in 25 % yield. In literature, in some settings, dehalogenation of the aryl halide as side‐reaction in Suzuki‐Miyaura coupling has been known. Employing 4‐vinylpyridine, 3‐vinylpyridine, and 2‐vinylpyridine precursors components furnished vinylpyridyl‐substituted PCPs (**19**–**21**). The corresponding scaffolds (**19**–**21**) were obtained in a yield of 46 %, 66 %, and 21 %, respectively. On *N*‐methylation, tetra‐cationic **22** along with tri‐cationic pyridinium **23** and vinylpyridinium‐modified PCP with spatial tuning of the positive charges (*para*‐, *meta*‐, and *ortho*‐*N*‐methylated cationic pyridinium groups) localized on the PCP‐scaffolds (**24** and **25**) were obtained employed their corresponding precursors. The synthesis procedure can be carried out on a multi‐gram scale, demonstrating their practicality and applicability to large‐scale production (Scheme 2). For synthesis details, see the Supporting Information.

**Scheme 3:**
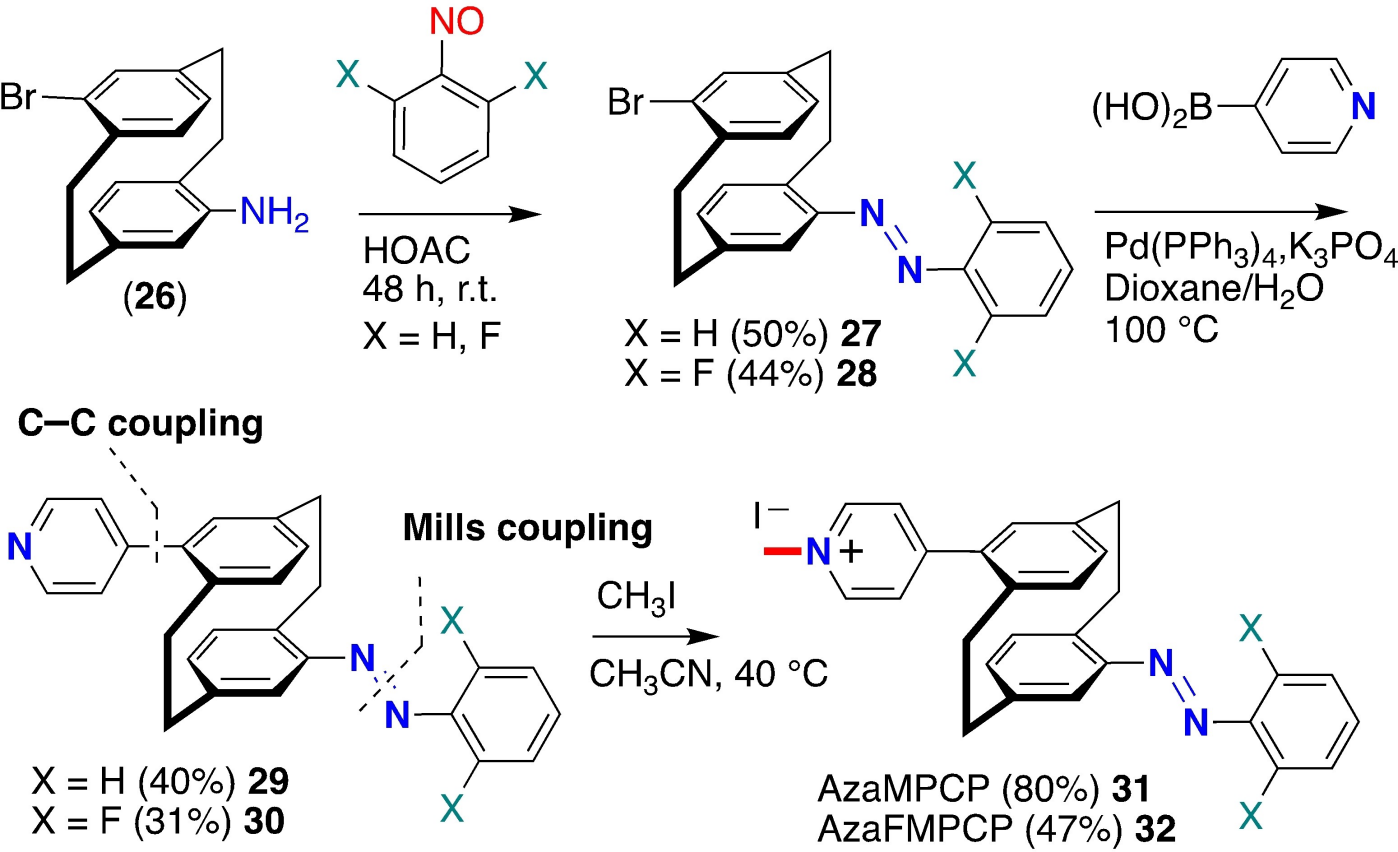
Synthesis of (*E*)‐azophenyl‐functionalized cationic pyridyl‐substituted PCPs (**31**, **32**) prepared through stepwise Mills/Suzuki‐Miyaura cross‐coupling reactions followed by on *N*‐methylation.

Our modular approach was extended for synthesizing azo‐functionalized cationic pyridyl‐modified PCPs through stepwise Mills/Suzuki‐Miyaura cross‐coupling reactions, previously optimized for non‐cyclophanyl aromatic scaffolds.^[17]^ The synthesis of the photoswitchable azobenzene that contains a diaryl diazene (−N=N−) core relies on the Mills reaction. Synthetic photoswitches are useful precursors for developing stimuli‐responsive systems with photo‐controlled capability for switching them on and off. Employing 4‐bromo‐16‐amino‐PCP (**26**) and nitrosoarene derivatives under acetic conditions incorporate photo‐responsive azobenzene (−N=N−) functional moiety and afforded products **27** and **28** (Scheme 3). The final azo‐derivatives were coupled via Suzuki coupling (**29** and **30**) and followed by methylation to give the target compounds **31** and **32**. Selective substitution of the azobenzene system such as the incorporation of fluorinated azobenzene side groups) are aimed to trigger variations in thermal stability and photochemical properties.^[18]^ In Suzuki coupling reactions, compounds with fluorine atoms substitution have lower yields. The PCPs on grafting with light‐responsive azobenzene functional core as side‐groups at pre‐defined positions imparts photosensitivity that can be remotely transformed on irradiation and undergoes reversible molecular motion (*trans* to *cis* photoisomerization), offering photo‐controlled smart functions.

**Figure 2:**
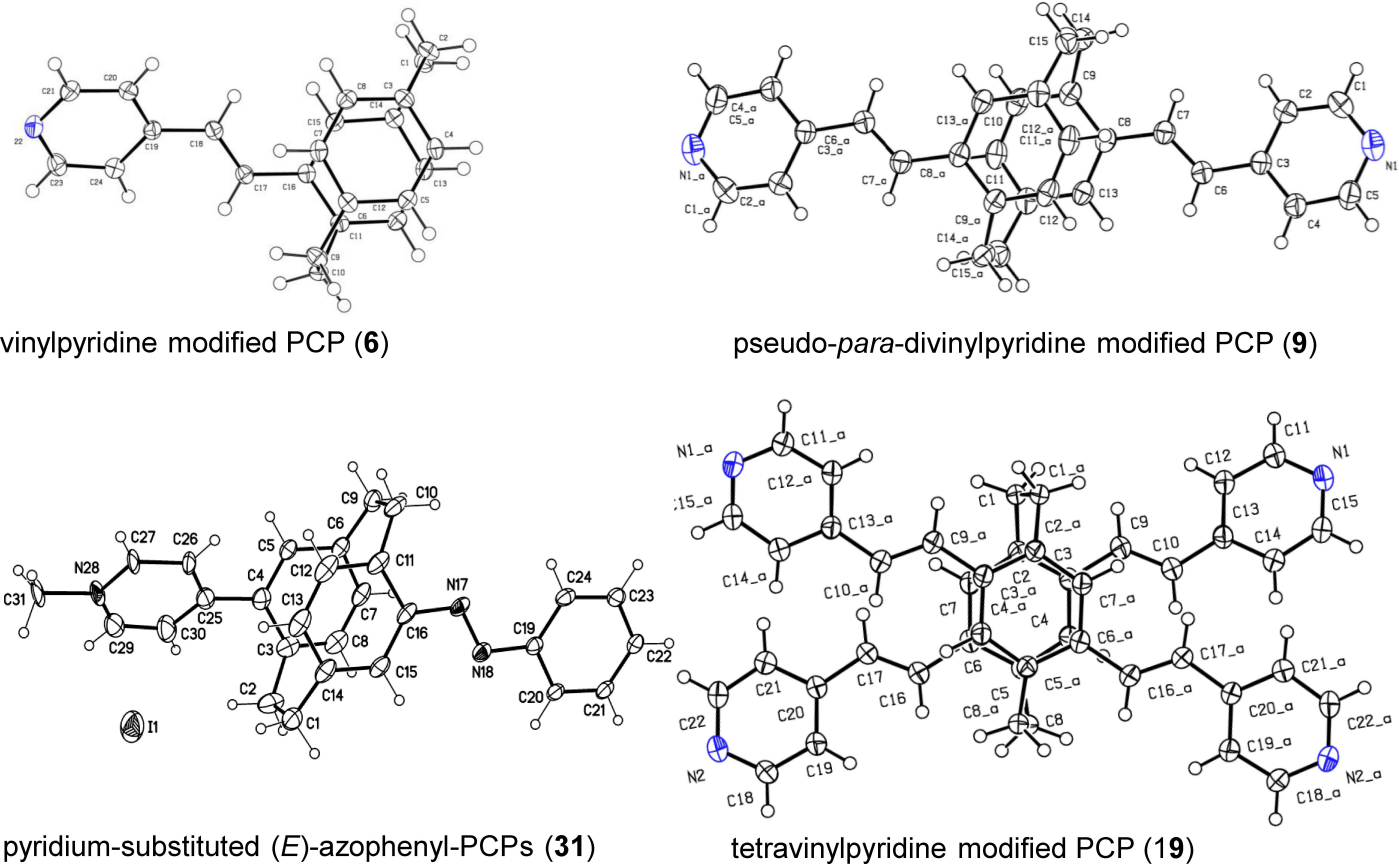
Single‐crystal X‐ray molecular structure of the mono‐ (**6**), pseudo‐para‐divinyl pyridine modified PCP (**9**), tetravinylpyridine modified PCP (**19**), and azo‐functionalized pyridinium‐PCP (**31**).

The functionalized PCP‐derived product formation was purified and characterized by detailed spectroscopic techniques and mass spectrometry. To determine molecular orientations and connectivity, crystallography becomes a privileged reliable form of evidence‐based structure confirmation albeit when single crystal analysis is possible. In this vein, well‐grown crystals were obtained for compounds (**6**, **9**, **19**, and **31**), and their molecular structures were further confirmed by single‐crystal X‐ray analysis (Figure 2).

In our search for molecular receptors (guest/dye) to develop a new and efficient host‐guest supramolecular assay that reaches high levels of binding affinity in aqueous media, we introduced cationic pyridinium‐modified PCP‐derived indicator dyes taking advantage of their innate chromophoric properties and ingenious features. Developing new analysis methods based on supramolecular sensing‐driven approaches with high levels of sensitivity and specificity holds great promise for diagnostics and chemical applications.^[19]^ In this endeavor, developing chemosensors that exhibit high affinity towards a range of bio‐relevant molecules, cucurbit[n]urils CB[n] become one of the prime candidates among well‐known host molecules form ultra‐stable supramolecular complexes with multiple guests under the cavity‐dependent confined environment.^[20]^ In our proof‐of‐concept investigations, the cationic pyridinium‐modified PCP as a guest combined with the macrocyclic host CB[n] was demonstrated as a useful chemosensing candidate.^[21]^ The PCP‐derivatives bind to CB[n], and the CB[n]‐based complexation (high‐affinity bindings) was investigated by direct‐binding assays, indicator displacement assays, and guest displacement assays that exhibit high sensitivity, fast response time, and technical simplicity. In our preliminary explorations, the PCP scaffold certificated a great affinity guest to CB[8] macrocyclic host, allowing for expanding the range of applications. Mono‐substituted pyridyl PCP was shown to have the highest binding constant with CB[8] (Ka=2×1013 M^−1^ in unbuffered water; Ka=5×1011 M^−1^ in water and phosphate buffer saline buffer). We have further demonstrated fluorescence‐detected circular dichroism for CB‐based supramolecular chiral host‐guest complexes exploiting planar chirality of the enantiopure PCP.^[22]^ The substitution pattern of the PCPs heavily influences not only the nature and molecular features of the PCP scaffold itself but on complexing with CB[n] can be reflected in fluorescent sensing properties as minor changes in the chromophore conjugation, their relative orientation, and substitution patterns of the cyclopentyl‐derived scaffolds can alter fluorescent sensing properties of the CB[n] host‐guest supramolecular system significantly. Beyond the concept of host‐guest molecular assays, this work offers several novelties. For instance, all of the bespoke pyridyl‐substituted PCP‐derivatives as ditopic and tetratopic 3D tectons reported here particularly appealing for exploring cyclophane‐based advanced material applications via self‐assembly inheriting the innate planar chirality and layered structure of the PCP such as functional metal‐bipyridinium frameworks. In this vein, cyclophanes are prepared on a large scale and investigations on structuring (non)coordination‐driven materials are currently underway.

## Conclusions

In summary, a new series of mono‐, di‐, tri‐, and tetra‐cationic pyridinium and vinylpyridinium‐modified [2.2]paracyclophanes as useful molecular tectons for supramolecular systems are presented. The modular 3D PCP scaffolds were prepared by regioselective functionalization, followed by successive transformations via Pd‐catalyzed Suzuki‐Miyaura/Mill coupling and Mizoroki‐Heck reactions. Furthermore, the PCPs on grafting with light‐responsive azobenzene core impart photosensitivity that can be remotely transformed on irradiation which might enable photo‐controlled smart molecular assay. The PCPs bearing pyridyl functionalities on *N*‐methylation were shown as efficient molecular receptors in CB[n]‐based host‐guest supramolecular systems, as demonstrated in our proof‐of‐concept preliminary investigations. This research on CB[n]‐based host‐guest supramolecular systems has been of fundamental interest and understanding, predominantly spanning molecular design and binding pathways. It remains to be explored whether PCP‐derived chromophore conjugation, their relative orientation, and substitution patterns of the differently‐functionalized cyclophanyl scaffolds could have critical effects on modulating/enhancing sensing properties of the CB[n] host‐guest supramolecular system.

By grafting characteristic features onto the PCP derivatives such as N/O metal‐coordination sites, planar chirality, and photo‐responsiveness to external stimuli, provide useful molecular blocks for constructing various molecular structures which can also inherent such innate features once assembled into materials such as coordination‐driven metal‐organic frameworks, metal‐organic cages/rings with metal ions/clusters or non‐coordination supramolecular assemblies via intermolecular interactions of halogen‐bonding or hydrogen bonds. Beyond the concept of host‐guest supramolecular systems, sketching out possible future research, we also believe these pyridine‐substituted ditopic and tetratopic PCPs have enormous potential to serve as coordination‐capable molecular tectons for engineering function‐inspired porous assemblies with new structural and functional attributes. By bridging cyclophanes chemistry with supramolecular chemistry strategies to form supramolecular assemblies points towards exciting new possibilities in cyclophane‐based materials applications

## Supporting Information

**Crystallographic data**: CCDC 2241449 (compound **6**); CCDC 2240975 (compound **9**) and CCDC 2240975 (compound **19**) contain the supplementary crystallographic data for this paper. Compound **31** due to the disorder and the squeezed out unknown solvent the data were not deposited into the Cambridge database, but could be used for determination of the constitution and conformation. These data can be obtained free of charge from The Cambridge Crystallographic Data Centre via www.ccdc.cam.ac.uk/data_request/cif.

Supporting Information is available and includes complete detailed synthetic procedures, spectroscopic techniques, and single‐crystal X‐ray analysis.

## Conflict of interests

The authors declare no competing financial interest.

1

## Supporting information

As a service to our authors and readers, this journal provides supporting information supplied by the authors. Such materials are peer reviewed and may be re‐organized for online delivery, but are not copy‐edited or typeset. Technical support issues arising from supporting information (other than missing files) should be addressed to the authors.

Supporting Information

## Data Availability

The data that support the findings of this study are available from the corresponding author upon reasonable request.
