# Supplementary material for: Synthesis of Mono‐, Di‐, Tri‐, and Tetra‐cationic Pyridinium and Vinylpyridinium Modified [2.2]Paracyclophanes: Modular Receptors for Supramolecular Systems
Source: ChemistryOpen. 2024 Mar 12;13(8):e202400024. doi: 10.1002/open.202400024 (PMC11319226; doi:10.1002/open.202400024)
Supplement: Supplementary file 1 — Supporting Information [file OPEN-13-e202400024-s001.pdf]

ChemistryOpen

Supporting Information

Synthesis of Mono-, Di-, Tri-, and Tetra-cationic Pyridinium and Vinylpyridinium Modified [2.2]Paracyclophanes: Modular Receptors for Supramolecular Systems

Yichuan Wang, Yuting Li, Olaf Fuhr, Martin Nieger, Zahid Hassan,* and Stefan Bräse*

Supporting Information

Synthesis of Mono-, Di-, Tri-, and Tetra-cationic Pyridinium and Vinylpyridinium Modified [2.2]Paracyclophanes: Modular Receptors for Supramolecular Systems

Dr. Yichuan Wang[1], Yuting Li[1], Dr. Olaf Fuhr[2], Dr. Martin Nieger[3], Dr. Zahid Hassan[1]* Prof. Stefan Bräse[1,4]*

[1] Institute of Organic Chemistry (IOC), Karlsruhe Institute of Technology (KIT), Fritz-Haber-Weg 6, 76131 Karlsruhe, Germany.

[2] Institute of Nanotechnology (INT) and Karlsruhe Nano Micro Facility (KNMFi), Karlsruhe Institute of Technology (KIT), Hermann-von-Helmholtz-Platz 1, 76344 Eggenstein-Leopoldshafen, Germany.

[3] Department of Chemistry, University of Helsinki, P. O. Box 55, 00014 University of Helsinki (Finland)

[4] Institute of Biological and Chemical Systems – Functional Molecular Systems (IBCS-FMS), Karlsruhe Institute of Technology (KIT), Hermann-von-Helmholtz-Platz 1, 76344 Eggenstein-Leopoldshafen, Germany.

*Corresponding Author(s): Zahid Hassan zahid.hassan@kit.edu; Stefan Bräse: braese@kit.edu

List of Contents for Experimental Section:

1. Materials and Methods
2. Synthetic Procedure Details
3. Characterization Data for Products
4. X-Ray Diffractometry

1 Materials and Methods

The starting materials, solvents, and reagents were purchased from abcr, Acros, Alfa Aesar, Apollo Scientific, Carbolution, ChemPUR, Fluka, Fluorochem, Merck, Riedel-de Haën, Sigma Aldrich, Strem, TCI, or Thermo Fisher Scientific and used without further purification unless stated otherwise.

Solvents of technical quality were purified by distillation or with the solvent purification system MB SPS5 (acetonitrile, dichloromethane, diethyl ether, tetrahydrofuran, toluene) from MBraun. Solvents of *p.a.* quality were purchased from Acros, Fisher Scientific, Sigma Aldrich, Roth, or Riedel-de Haën and were used without further purification. Diphenyl ether and α,α,α,-trifluoro toluene were distilled over calcium hydride. *n*-Pentane was distilled over sodium and benzophenone. Other solvents were obtained from commercial suppliers: anhydrous benzene (Sigma Aldrich, <0.005% water), anhydrous *N*,*N*-dimethylformamide (Sigma Aldrich, <0.005% water), anhydrous 1,4-dioxane (Sigma Aldrich, <0.005% water), anhydrous dimethyl sulfoxide (Sigma Aldrich, <0.005% water), anhydrous ethanol (Sigma Aldrich, <0.005% water), anhydrous methanol (Sigma Aldrich, <0.005% water), anhydrous isopropanol (Sigma Aldrich, <0.005% water).

Oxygen-free solvents were obtained by the freeze-pump-thaw (three cycles) technique.

Air- and moisture-sensitive reactions were carried out under an argon atmosphere in oven-dried glassware using standard Schlenk techniques.

An MBraun LABmaster with an argon atmosphere was used for reaction setups in the glove box. All materials used were dried for at least 24 h at 100 °C before bringing them into the glove box.

Flat-bottom crimp neck vials from ChromaGlobe with aluminum crimp caps were used for certain reactions.

Liquids were added with a stainless-steel cannula, and solids were added in a powdered shape.

Reactions at low temperatures were cooled using flat dewars produced by ISOTHERM (Karlsruhe) with water/ice or isopropanol/anhydrous ice mixtures.

Solvents were evaporated under reduced pressure at 45 °C using a rotary evaporator. For solvent mixtures, each solvent was measured volumetrically.

Flash column chromatography was performed using Merck silica 60 (0.040 × 0.063 mm, 230–400 mesh ASTM) and quartz sand (glowed and purified with hydrochloric acid).

The Rayonet reactor Model RPR-100 with (16) 14W light bulbs (254 nm) was used for the irradiation with UV light.

Reaction Monitoring

All reactions were monitored by thin-layer chromatography (TLC) using silica-coated aluminum plates (Merck, silica 60, F254). UV active compounds were detected with a UV lamp at 254 nm and 366 nm excitation. Vanillin, basic potassium permanganate, ninhydrin, or Seebach solution was used as a TLC stain when required.

GC-MS (gas chromatography-mass spectrometry) measurements were performed on an Agilent Technologies model 6890N (electron impact ionization), equipped with an Agilent 19091S-433 column (5% phenyl methyl siloxane, 30 m, 0.25 μm) and a 5975B VL MSD detector with a turbopump. Helium was used as a carrier gas.

Nuclear Magnetic Resonance Spectroscopy (NMR)

NMR spectra were recorded on a Bruker Avance 400 NMR instrument at 400 MHz for ^{1}H NMR, 101 MHz for ^{13}C NMR, or a Bruker Avance 500 NMR instrument at 500 MHz for ^{1}H NMR, 126 MHz for ^{13}C NMR.

The NMR spectra were recorded at room temperature in deuterated solvents acquired from Eurisotop, Sigma Aldrich, or Deutero. The chemical shift δ is displayed in parts per million [ppm] and the references used were the ^{1}H and ^{13}C peaks of the solvents themselves:

d_1-chloroform ($CDCl_3$): 7.26 ppm for ^{1}H and 77.16 ppm for ^{13}C

d_6-dimethyl sulfoxide (DMSO-d_6): 2.50 ppm for ^{1}H and 39.52 ppm for ^{13}C

d_4-methanol (CD_3OD): 3.31 ppm for ^{1}H and 49.00 ppm for ^{13}C

d_3-acetonitrile (CD_3CN): 1.94 ppm for ^{1}H and 118.26 ppm for ^{13}C

For the characterization of centrosymmetric signals, the median point was chosen for multiplets in the signal range. The following abbreviations were used to describe the proton splitting pattern: d = doublet, t = triplet, m = multiplet, dd = doublet of a doublet, ddd = doublet of doublet of a doublet, dddd = doublet of doublet of doublet of a doublet, dt = doublet of a triplet. Absolute values of the coupling constants "*J*" are given in Hertz [Hz] in absolute value and decreasing order. Signals of the ^{13}C spectrum were assigned by distortionless enhancement by polarization transfer (DEPT) spectra DEPT90 and DEPT135 or phase-edited heteronuclear single quantum coherence (HSQC). They were specified in the following way: + = primary or tertiary carbon atoms (positive phase), – = secondary carbon atoms (negative phase), and C_q = quaternary carbon atoms (no signal).

Infrared Spectroscopy (IR)

The infrared spectra were recorded with a Bruker Alpha P instrument. All samples were measured by attenuated total reflection (ATR). The positions of the absorption bands are given in wavenumbers $\tilde{\nu}$ in cm^{-1} and were measured in the range from 3600 cm^{-1} to 500 cm^{-1}.

Characterization of the absorption bands was done in dependence on the absorption strength with the following abbreviations: vs (very strong, 0−9%), s (strong, 10−39%), m (medium, 40−69%), w (weak, 70−89%), vw (very weak, 90−100%).

Mass Spectrometry (MS)

Electron ionization (EI) and fast atom bombardment (FAB) experiments were conducted using a Finnigan, MAT 90 (70 eV) instrument, with 3-nitrobenzyl alcohol (3-NBA) as matrix and reference for high resolution. For the interpretation of the spectra, molecular peaks $[M]^{+}$, peaks of protonated molecules $[M+H]^{+}$, and characteristic fragment peaks are indicated with their mass-to-charge ratio (*m/z*), and their intensity in percent, relative to the base peak (100%) is given. In the case of high-resolution measurements, the maximum tolerated error is ±5 ppm.

APCI and ESI experiments were recorded on a Q-Exactive (Orbitrap) mass spectrometer (Thermo Fisher Scientific, San Jose, CA, USA) equipped with a HESI II probe to record high resolution. The tolerated error is ±5 ppm of the molecular mass. The spectra were interpreted by molecular peaks $[M]^{+}$, peaks of protonated molecules $[M+H]^{+}$, and characteristic fragment peaks and indicated with their mass-to-charge ratio (*m/z*).

2 Synthesis

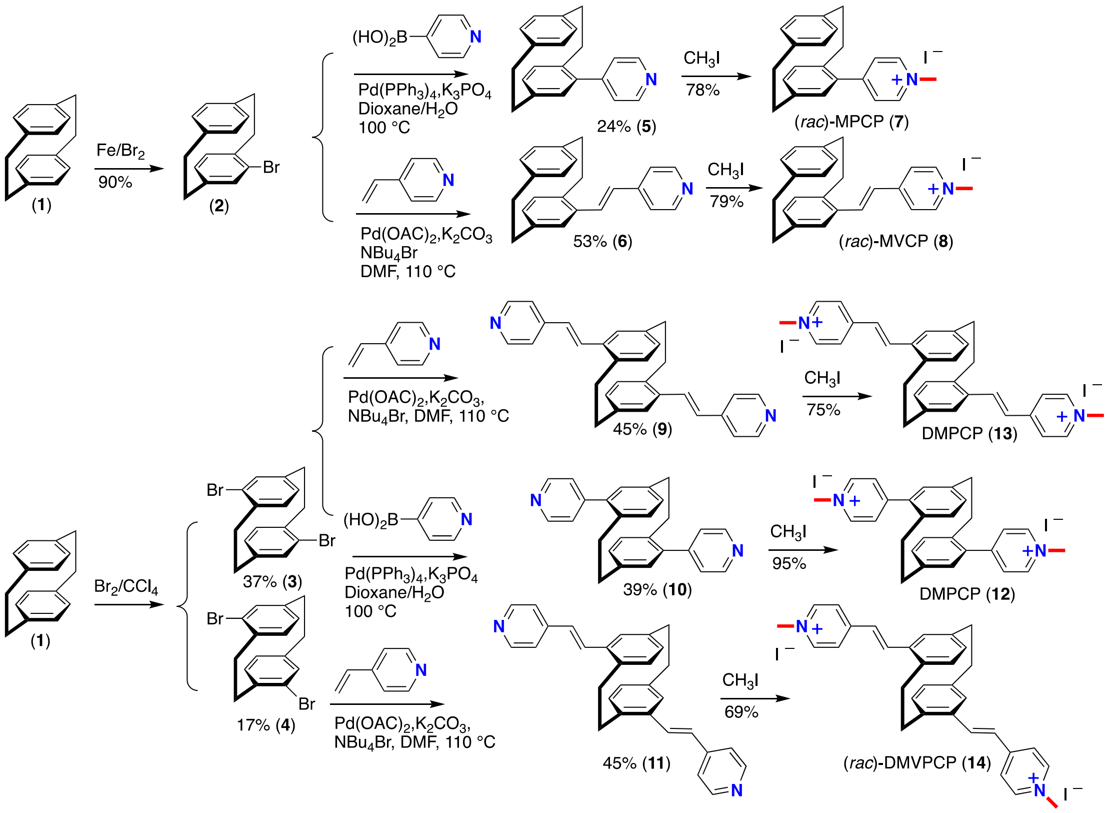

Scheme 1. *Synthesis of mono-* ***7*** *and differently-functionalized di-cationic pyridinium* ***12*** *and vinylpyridinium-modified PCPs* ***(8, 13,*** *and* ***14)*** *prepared via Pd-catalyzed Suzuki-Miyaura and Heck cross-coupling reaction, followed by on N-methylation.*

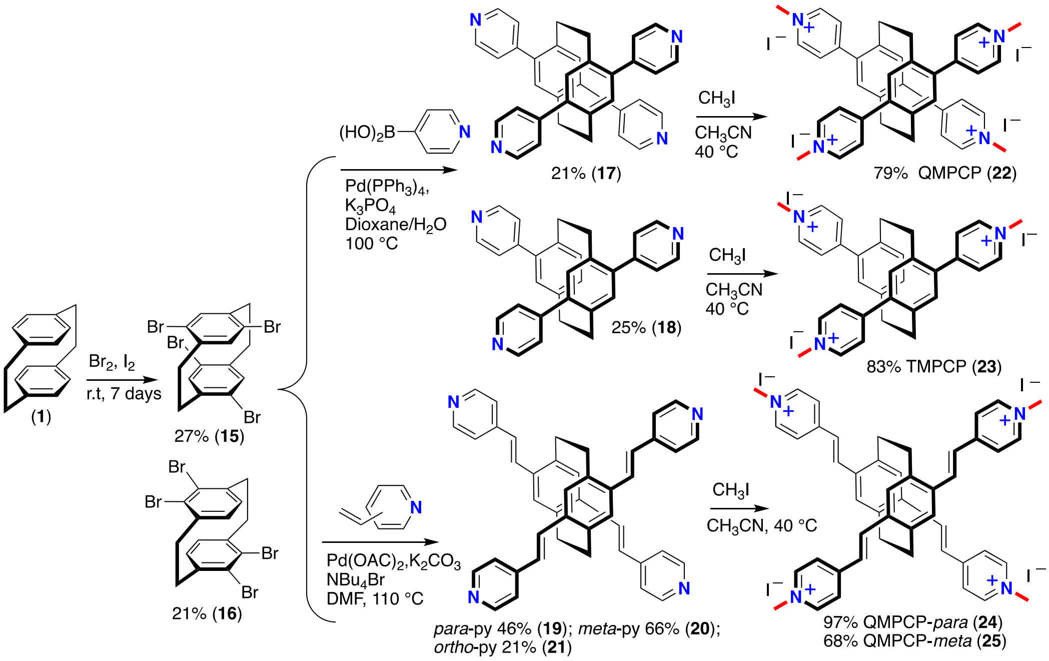

Scheme 2. *Synthesis of tetra-cationic pyridinium* ***22****, tri-* ***23*** *and vinylpyridinium-modified PCPs (****24, 25****) prepared via Pd-catalyzed Suzuki-Miyaura and Heck cross-coupling reaction employing* ***15****, followed by on N-methylation.*

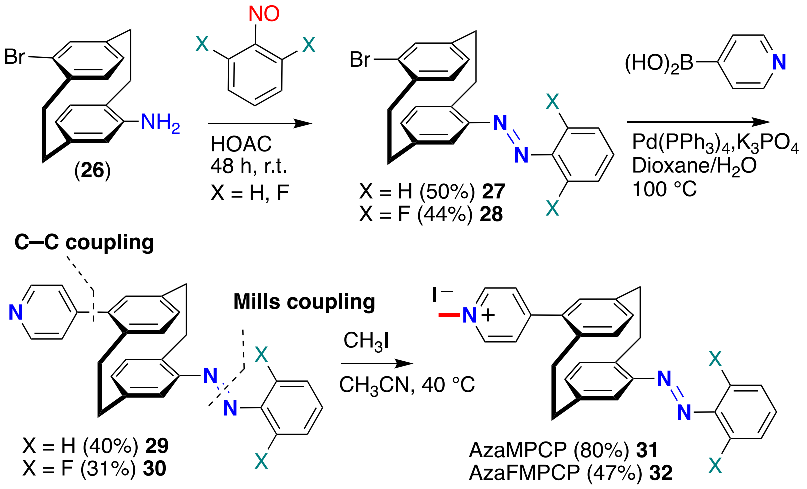

Scheme 3. *Synthesis of (E)-azophenyl-functionalized cationic pyridyl-substituted PCPs (**31**, **32**) prepared through stepwise Mills/Suzuki-Miyaura cross-coupling reactions followed by on N-methylation.*

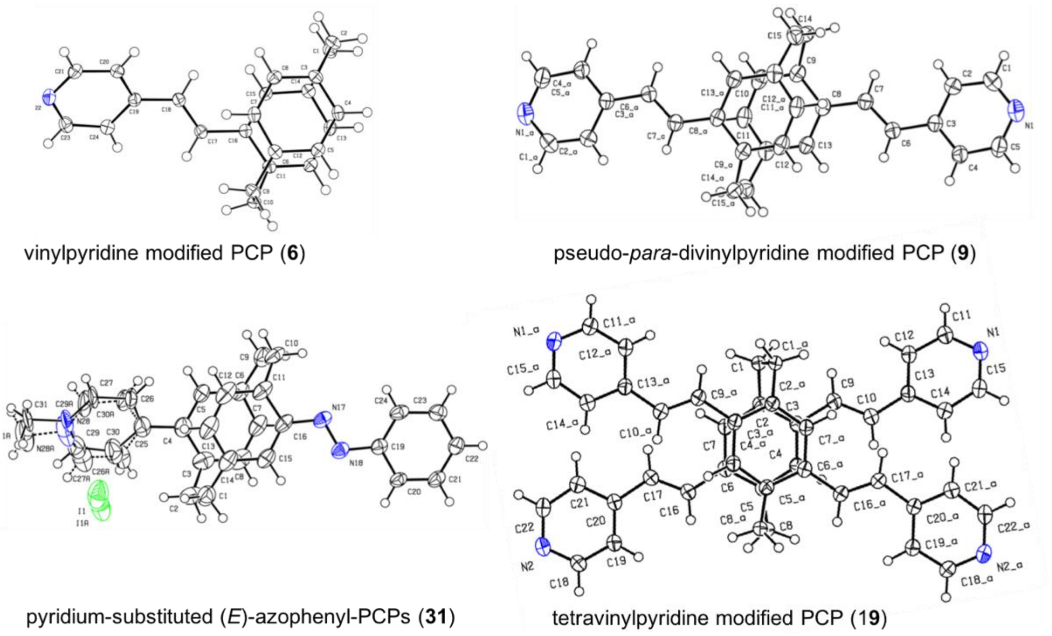

Figure 2. *Single-crystal X-ray molecular structure of the mono- (**6**), pseudo-para-divinyl pyridine modified PCP (**9**), tetravinylpyridine modified PCP (**19**), and azo-functionalized pyridinium-PCP(**31**).*

Characterization Data for Products:

^{1}HNMR, and ^{13}CNMR spectra for the newly synthasized compounds are provided herewith. Characterization data for known mono- (**2**), di- (**3**), and tetrabromo-substituted PCPs (**15** and **16**) (mentioned in this manusrcipt) are available in the literature (B. Konig, B. Knieriem, A. de Meijere, *Chem. Ber*. **1993**, *126*, 1643-I650).

(*rac*)-4-(4'-Pyridyl) [2.2]paracyclophane (5):

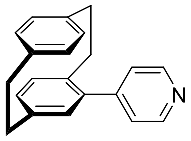

A vessel was charged with (*rac*)-4-bromo[2.2]paracyclophane (1282 mg, 3.0 mmol, 1.0 equiv.), Pyridine-4-boronic acid (1082 mg, 6.0 mmol, 2.0 equiv.), Palladium-tetrakis(triphenylphosphine) (208 mg, 0.18 mmol, 0.04 equiv.), potassium phosphate (1590 mg, 7.5 mmol, 1.6 equiv.) under argon atmosphere. Then dioxane (16 mL) and water (8 mL) was added, the mixture heated under reflux for 16 h. The reaction mixture was then cooled to room temperature, extracted with ether (3 × 20 mL), the extracts were dried ($MgSO_4$) and evaporated under reduced pressure. The crude solid was purified by column chromatography on silica gel by cyclohexane/ethyl acetate 2:1 to obtain the title compound (304 mg, 1.1 mmol, 24%) as a white solid. **R_f** = 0.43 (cyclohexane/ethyl acetate 2:1). **^{1}H NMR (400 MHz, $CDCl_3$, ppm):** δ = 8.70 (d, *J* = 5.6 Hz, 2H, H_{Py}), 7.42 (dd, *J* = 4.6, 1.4 Hz, 2H, H_{Py}), 6.64 (dd, *J* = 8.0, 3.5 Hz, 2H, H_{Ar}), 6.59 (dd, *J* = 7.8, 5.0 Hz, 3H, H_{Ar}), 6.54 (d, *J* = 2.0 Hz, 2H, H_{Ar}), 3.40 (ddd, *J* = 12.6, 10.1, 2.9 Hz, 1H, H_{PC}), 3.20–3.11 (m, 2H, H_{PC}), 3.09–2.88 (m, 3H, PC-H), 2.66 (ddd, *J* = 13.1, 10.1, 4.5 Hz, 1H, H_{PC}). **^{13}C NMR (101 MHz, CDCl3, ppm):** δ = 149.7 (+, CH, 2C, C_{Py}), 148.9 (C_q, C_{Py}), 140.2 (C_q, C_{Ar}), 139.6 (C_q, C_{Ar}), 139.5 (C_q, C_{Ar}), 139.1 (C_q, C_{Ar}), 137.3 (C_q, C_{Ar}), 136.2 (+, CH, C_{Ar}), 133.6 (+, CH, C_{Ar}), 133.3 (+, CH, C_{Ar}), 139.7 (+, CH, C_{Ar}), 132.0 (+, CH, C_{Ar}), 132.0 (+, CH, C_{Ar}), 129.7 (+, CH, C_{Ar}), 124.6 (+, CH, 2C, C_{Py}), 35.5 (–, CH_2), 35.2 (–, CH_2), 34.9 (–, CH_2), 33.9 (–, CH_2). **MS (ESI, 70 eV, 20 °C, %):** m/z = 286/287 (100/22) $[M+H]^+$. **HRMS-ESI (m/z):** $[M+H]^+$, calc. for $C_{21}H_{20}N$, 286.1590; found: 286.1588. **IR (ATR, ṽ)** = 3023 (w), 2953 (w), 2927 (s), 2897 (w), 2854 (m), 1594 (vs), 1541 (w), 1496 (w), 1475 (w), 1439 (w), 1409 (s), 1400 (s), 1213 (w), 1092 (w), 992 (w), 898 (m), 850 (s), 824 (vs), 793 (s), 734 (m), 717 (vs), 667 (w), 647 (vs), 622 (vs), 594 (s), 558 (s), 516 (vs), 483 (vs), 436 (m), 424 (m), 378 (w) cm^{-1}.
https://dx.doi.org/10.14272/reaction/SA-FUHFF-UHFFFADPSC-KMTTZSIPTT-UHFFFADPSC-NUHFF-NUHFF-NUHFF-ZZZ

(*rac*)-4-(*N*-methyl-4'-pyridinium)[2.2]paracyclophane iodide (MPCP; 7):

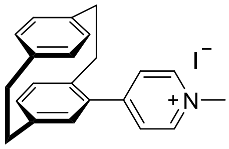

A solution of a mixture of (*rac*)-4-(4'-pyridyl)[2.2]paracyclophane (148 mg, 0.5 mmol, 1.0 equiv.) and methyl iodide (3.2 mmol, 0.2 mL) in acetonitrile (12 mL) was stirred at 40 °C for 12 h protected from light. After the solution was cooled to room temperature, removed solvent under reduced pressure to give a residue, washed with water (20 mL), and dried in air to yield (172 mg, 0.40 mmol, 78%) a light yellow solid. **^{1}H NMR (400 MHz, $CDCl_3$, ppm):** δ = 9.22 (d, *J* = 6.5 Hz, 2H, H_{Py}), 8.08–8.01 (m, 2H, H_{Py}), 6.72–6.68 (m, 3H, H_{Ar}), 6.66–6.56 (m, 3H, H_{Ar}), 6.44 (dd, *J* = 7.9, 2.0 Hz, 1H, H_{Ar}), 4.70 (s, 3H, CH3), 3.34 (ddd, *J* = 13.7, 10.1, 3.6 Hz, 1H, H_{PC}), 3.25–3.05 (m, 5H, H_{PC}), 3.01–2.90 (m, 1H, H_{PC}), 2.79 (ddd, J = 14.1, 10.0, 5.1 Hz, 1H, H_{PC}). **^{13}C NMR (100 MHz, $CDCl_3$, ppm):** δ = 145.1 (+, CH, 2C, C_{Py}), 141.4 (C_q, C_{Py}), 139.8 (C_q, C_{Ar}), 139.1 (C_q, C_{Ar}), 137.1 (+, CH, 2C, C_{Ar}), 136.4 (C_q, 2C, C_{Ar}), 133.7 (+, CH, C_{Ar}), 132.8 (C_q, C_{Ar}), 132.8 (+, CH, C_{Ar}), 132.5 (+, CH, C_{Ar}), 131.9 (+, CH, C_{Ar}), 129.8 (C_q, C_{Ar}), 127.9 (+, CH, 2C, C_{Py}), 48.7 (+, CH_3), 35.3(–, CH_2), 35.3 (–, CH_2), 34.0 (–, CH_2), 29.7 (–, CH_2). **MS (ESI, 70 eV, 20 °C, %):** m/z = 300/301 (100/23) $[M-I]^+$, **HRMS-ESI (m/z):** $[M-I]^+$, calc. for $C_{22}H_{22}N$, 300.1747; found: 300.1744. **IR (ATR, ṽ)** = 3504 (m), 3489 (m), 3465 (m), 3441 (m), 2924 (m), 1638 (vs), 1561 (m), 1514 (s), 1500 (m), 1460 (m), 1220 (m), 1197 (s), 849 (vs), 805 (s), 720 (vs), 646 (vs), 554 (m), 514 (vs), 484 (vs), 462 (s), 448 (vs), 438 (vs), 428 (vs), 412 (vs), 402 (vs), 387 (vs), 378 (vs) cm^{-1}.
https://dx.doi.org/10.14272/reaction/SA-FUHFF-UHFFFADPSC-HTDJDZOPDB-UHFFFADPSC-NUHFF-MUHFF-NUHFF-ZZZ

(*rac*)-4-(Vinyl-4'-pyridyl)[2.2]paracyclophane (6):

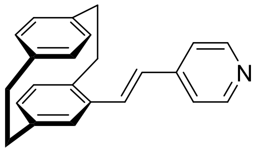

A vessel was charged with (*rac*)-4-bromo[2.2]paracyclophane (1.44 g, 5.0 mmol, 1.0 equiv.), 4-vinylpyridine (1.05 g, 10.0 mmol, 2.0 equiv.), Palladium acetate (56 mg, 0.25 mmol, 0.05 equiv.), tetrapropylammonium bromide (1.33 g, 5.0 mmol, 1.0 equiv.), K_2CO_3 (1.73 mg, 12.5 mmol, 2.5 equiv.), and DMF (25 mL) under argon. The reaction mixture heated at 110 °C for 24 h. Then the reaction was cooled to room temperature, diluted with CH_2Cl_2, and washed with brine. The organic layer was dried with Na_2SO_4 and concentrated. The residual liquid (containing a small amount of DMF) was precipitated with hexane and collected by filtration. The crude solid was purified by column chromatography on silica gel by cyclohexane/ethyl acetate 2:1 to obtain the title compound (820 mg, 2.6 mmol, 53%) as an orange solid. **R_f** = 0.45 (dichloromethane/methanol 20:1). **^{1}H NMR (400 MHz, $CDCl_3$, ppm):** δ = 8.64–8.58 (m, 2H, H_{Py}), 7.43–7.36 (m, 3H, H_{Py}, C*H*=CH), 6.80 (d, *J* = 16.1 Hz, 1H, H_{Ar}), 6.70 (d, *J* = 1.8 Hz, 1H, H_{Ar}), 6.62 (dd, *J* = 7.9, 1.8 Hz, 1H, H_{Ar}), 6.57–6.53 (m, 3H, H_{Ar}), 6.50 (d, *J* = 7.8 Hz, 1H, H_{Ar}), 6.43 (dd, *J* = 7.9, 1.8 Hz, 1H, H_{Ar}), 3.64–3.53 (m, 1H, H_{PC}), 3.25–3.10 (m, 3H, H_{PC}), 3.10–2.86 (m, 4H, H_{PC}). **^{13}C NMR (100 MHz, $CDCl_3$, ppm):** δ = 150.3 (+, CH, 2C, C_{Py}), 145.1 (C_q, C, C_{Py}), 140.2 (C_q, C, C_{Ar}), 139.4 (C_q, C, C_{Ar}), 139.1 (Cq, C, C_{Ar}), 136.3 (Cq, C, C_{Ar}), 135.2 (+, CH, C_{Ar}), 133.1 (+, CH, C_{Ar}), 133.1 (+, CH, C_{Ar}), 133.0 (+, CH, 2C, C_{Ar}), 131.8 (+, CH, C_{Ar}), 131.1 (+, *C*H=CH), 130.5 (+, CH, C_{Ar}), 130.1 (+, CH, C_{Ar}), 126.3 (+, CH=*C*H), 120.9 (+, CH, 2C, C_{Py}), 35.5 (–, CH_2), 35.2 (–, CH_2), 35.1 (–, CH_2), 33.9 (–, CH_2). **MS (EI, 70 eV, 20 °C, %):** *m/z* = 312/313 (100/32) $[M+H]^+$. **HRMS-EI (*m/z*):** $[M+H]^+$, calc. for $C_{23}H_{21}N$, 312.1747; found: 312.1742. **IR (ATR, ṽ)** = 2927 (m), 2856 (w), 1591 (vs), 1409 (s), 1214 (w), 1082 (w), 958 (vs), 948 (s), 936 (m), 895 (m), 875 (s), 857 (m), 846 (s), 796 (vs), 775 (m), 737 (w), 718 (vs), 653 (s), 633 (s), 584 (s), 554 (s), 544 (s), 520 (vs), 513 (vs), 482 (s), 467 (s), 455 (s), 431 (m), 411 (w), 394 (w), 384 (m) cm^{-1}.

https://dx.doi.org/10.14272/reaction/SA-FUHFF-UHFFFADPSC-GWWUWJSSYC-UHFFFADPSC-NUHFF-NXJJT-NUHFF-ZZZ

(*rac*)-4-((*E*)-Vinyl-*N*-methyl-4'-pyridinium)[2.2]paracyclophane iodide (MVCP; 8):

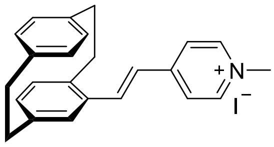

A solution of a mixture of (*rac*)-4-(vinyl-4'-pyridyl)[2.2]paracyclophane iodide (105 mg, 0.50 mmol, 1.0 equiv.) and methyl iodide (3.21 mmol, 0.2 mL) in acetonitrile (10 mL) was stirred at 40 °C for 16 h protected from light. After the solution was cooled to room temperature, removed solvent under reduced pressure to give a residue, washed with water (20 mL), and dried in air to yield (121 mg, 0.26 mmol, 79%) a brown yellow solid. **R_f** = 0.5 (dichloromethane/methane 5:1). **^{1}H NMR (400 MHz, DMSO-d_6, ppm):** δ = 8.87 (d, *J* = 6.5 Hz, 2H, H_{Py}), 8.38 (d, *J* = 6.6 Hz, 2H, H_{Py}), 7.96 (d, *J* = 16.1 Hz, 1H, CH=CH), 7.30 (d, *J* = 16.1 Hz, 1H, CH=CH), 6.96 (d, *J* = 1.7 Hz, 1H, H_{Ar}), 6.63 (dd, *J* = 7.8, 1.7 Hz, 1H, H_{Ar}), 6.59–6.52 (m, 3H, H_{Ar}), 6.45 (q, *J* = 1.1 Hz, 2H, H_{Ar}), 4.28 (s, 3H, CH_3), 3.84–3.72 (m, 1H, H_{PC}), 3.13–3.07 (m, 3H, H_{PC}), 3.04–2.97 (m, 3H, H_{PC}), 2.93–2.88 (m, 1H, H_{PC}). **^{13}C NMR (101 MHz, DMSO-d_6, ppm):** δ = 153.3 (C_q, C_{Ar}), 145.4 (+, 2C, CH, C_{Py}), 141.5 (C_q, C_{Ar}), 140.7 (C_q, C_{Ar}), 139.6 (C_q, C_{Ar}), 139.4 (C_q, C_{Ar}), 138.9 (+, CH=CH), 135.8 (C_q, C_{Ar}), 135.7 (+, CH, C_{Ar}), 135.0 (+, CH, C_{Ar}), 133.6 (+, CH, C_{Ar}), 133.5 (+, CH, C_{Ar}), 132.1 (+, CH, C_{Ar}), 131.3 (+, CH, C_{Ar}), 130.7 (+, CH, C_{Ar}), 124.1 (+, 2C, CH, C_{Py}), 123.9 (+, CH=CH), 47.3 (+, CH_3), 35.3 (–, CH_2), 35.2 (–, CH_2), 35.0 (–, CH_2), 33.6 (–, CH_2). **MS (EI, m/z, 70 eV, 70 °C):** 326.3 (100) $[M-I]^+$, 326 (100), 327 (25). **HRMS–EI (m/z):** $[M-I]^+$ calc. for $C_{24}H_{24}N$, 326.1903; found: 326.1909. **IR (ATR, ṽ)** = 3009 (m), 2922 (m), 2850 (m), 1639 (s), 1612 (vs), 1587 (s), 1564 (m),

1514 (s), 1500 (m), 1480 (m), 1468 (m), 1449 (m), 1319 (m), 1183 (vs), 1154 (s), 1143 (s), 1118 (s), 1101 (s), 1082 (s), 1050 (m), 963 (s), 936 (m), 885 (m), 868 (s), 819 (vs), 798 (s), 728 (m), 717 (s), 642 (s), 615 (m), 582 (m), 520 (s), 506 (vs), 489 (s), 470 (m), 465 (m), 453 (m), 446 (m), 428 (m), 419 (m), 401 (m), 384 (m) cm^{-1}.
https://dx.doi.org/10.14272/reaction/SA-FUHFF-UHFFFADPSC-GRWXNDHDQX-UHFFFADPSC-NUHFF-MABGN-NUHFF-ZZZ.1

4,16-Di(4'-pyridyl)[2.2]paracyclophane (10):

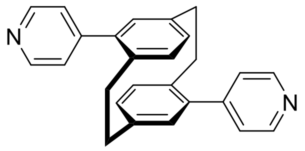

A vessel was charged with 4, 16-dibromo[2.2]paracyclophane (545 mg, 1.49 mmol, 1.00 equiv.), pyridine-4-boronic acid (458 mg, 3.73 mmol, 2.50 equiv.), palladium-tetrakis(triphenylphosphine) (103 mg, 89 µmol, 0.06 equiv.), potassium phosphate (637 mg, 4.47 mmol, 2.00 equiv.), 1,4-dioxane (12 mL) and water (6 mL) under argon atmosphere. The mixture was heated at 100 °C for 16 h. Then the reaction mixture was cooled down to room temperature, extracted with ether (3 × 20 mL), and the extracts were dried ($MgSO_4$) and evaporated under reduced pressure. The residue was purified by column chromatography to give 4,16-di(4'-pyridyl)[2.2]paracyclophane as a white solid (213 mg, 588 µmol, 39%). $\mathbf{R_f}$ = 0.25 (dichloromethane /ethyl acetate 1:3). **^{1}H NMR (400 MHz, $CDCl_3$, ppm):** δ = 8.66 (d, J = 6.1 Hz, 4H, H_{Py}), 7.37 (d, J = 6.0 Hz, 4H, H_{Py}), 6.62 (dd, J = 4.9, 2.9 Hz, 4H, H_{Ar}), 6.53 (dd, J = 7.9, 1.9 Hz, 2H, H_{Ar}), 3.37 (ddd, J = 13.9, 10.0, 4.1 Hz, 2H, H_{PC}), 3.02 (ddd, J = 14.0, 10.2, 4.7 Hz, 2H, H_{PC}), 2.85 (ddd, J = 14.1, 10.2, 4.1 Hz, 2H, H_{PC}), 2.73 (ddd, J = 13.3, 10.0, 4.7 Hz, 2H, H_{PC}). **^{13}C NMR (100 MHz, $CDCl_3$, ppm):** δ = 150.2 (+, CH, 4C, C_{Py}), 148.4 (C_q, 2C, C_{Py}), 140.2 (C_q, 2C, C_{Ar}), 139.6 (C_q, 2C, C_{Ar}), 137.2 (C_q, 2C, C_{Ar}), 135.1 (+, CH, 2C, C_{Ar}), 132.3 (+, CH, 2C, C_{Ar}), 130.4 (+, CH, 2C, C_{Ar}), 124.5 (+, CH, 4C, C_{Py}), 34.7 (–, 2C, CH_2), 33.6 (–, 2C, CH_2). **MS (ESI, 70 eV, 20 °C, %):** m/z = 363 (100) $[M+H]^+$, 364 (28) $[M+H]^+$,156 (24) $[C_{11}H_{10}N]^+$, 286 (23) $[M–C_5H_2N]^+$. **HRMS-ESI (m/z):** $[M+H]^+$, calc. for $C_{26}H_{23}N_2$, 363.1856; found: 363.1850. **IR (ATR, ṽ)** = 2948 (w), 2929 (m), 2895 (w), 2853 (w), 1591 (vs), 1538 (m), 1475 (m), 1453 (w), 1436 (m), 1415 (m), 1400 (s), 1322 (w), 1213 (m), 1181 (m), 1118 (m), 1103 (m), 1065 (m), 990 (m), 914 (m), 873 (w), 822 (vs), 751 (m), 734 (s), 721 (vs), 707 (s), 694 (s), 667 (s), 653 (vs), 620 (vs), 608 (m), 571 (vs), 558 (s), 538 (vs), 483 (vs), 438 (m), 422 (s), 392 (m), 381 (m) cm^{-1}.
https://dx.doi.org/10.14272/reaction/SA-FUHFF-UHFFFADPSC-VKMYNZXLPC-UHFFFADPSC-NUHFF-NUHFF-NUHFF-ZZZ

4,16-Di(*N*-methyl-4'-pyridinium)[2.2]paracyclophane iodide (pseudo-*para*-DMPCP) (12):

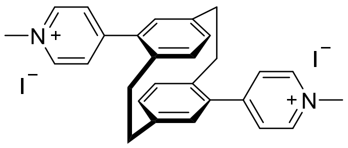

A solution of a mixture of 4,16-di-(4'-pyridyl)[2.2]paracyclophane (74 mg, 110 µmol, 1.00 equiv.) and methyl iodide (3.21 mmol, 200 µmL) in acetonitrile (5 mL) was stirred at 40 °C for 16 h protected from light. After the solution was cooled to room temperature, removed solvent under reduced pressure to give a residue, washed with DCM (20 mL), and dried in air to yield (125 mg, 193 µmol, 95%) a light-yellow solid. **^{1}H NMR (400 MHz, DMSO-d6, ppm):** δ = 9.05 (d, J = 6.4 Hz, 4H, H_{Py}), 8.32 (d, J = 6.9 Hz, 4H, H_{Py}), 7.10 (d, J = 7.9 Hz, 2H, H_{Ar}), 7.05 (d, J = 1.8 Hz, 2H, H_{Ar}), 6.58 (dd, J = 7.8, 1.8 Hz, 2H, H_{Ar}), 4.40 (s, 6H, CH_3), 3.36 (td, J = 9.7, 4.8 Hz, 2H, H_{PC}), 3.23 (ddd, J = 14.0, 9.9, 4.9 Hz, 2H, H_{PC}), 2.99 (ddd, J = 13.6, 9.9, 4.0 Hz, 2H, H_{PC}), 2.71 (ddd, J = 13.7, 10.2, 5.0 Hz, 2H, H_{PC}). **^{13}C NMR (101 MHz, DMSO-d6, ppm):** δ = 155.4 (+, CH, 4C, C_{Py}), 145.9 (C_q, 2C, C_{Py}), 140.8 (C_q, 2C, C_{Ar}), 139.0 (C_q, 2C, C_{Ar}), 136.9 (C_q, 2C, C_{Ar}),

136.0 (+, CH, 2C, C_{Ar}), 133.1 (+, CH, 2C, C_{Ar}), 132.7 (+, CH, 2C, C_{Ar}), 127.8 (+, CH, 4C, C_{Py}), 47.6 (+, 2C, CH_3), 34.5 (–, 2C, CH_2), 33.5 (–, 2C, CH_2). **MS (ESI, 70 eV, 20 °C, %):** m/z = 196/197 (100/97) $[M-2I]^{2+}$. **HRMS-ESI (m/z):** $[M-2I]^{2+}$, calc. for $C_{28}H_{28}N_2$, 196.1121; found: 196.1121. **IR (ATR, ṽ):** = 1636 (m), 1215 (m), 1188 (s), 1067 (vs), 972 (w), 847 (s), 805 (w), 557 (m), 484 (vs), 456 (vs), 442 (vs), 388 (s) cm^{-1}.
https://dx.doi.org/10.14272/reaction/SA-FUHFF-UHFFFADPSC-PNLSHCRELK-UHFFFADPSC-NUHFF-LUHFF-NUHFF-ZZZ

4,16-Di(4'-pyridyl-(*E*)-vinyl[2.2]paracyclophane (9):

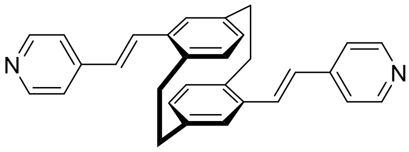

A vessel was charged with 4,16-dibromo[2.2]paracyclophane (5.49 g, 15.0 mmol, 1.00 equiv), 4-vinylpyridine (7.89 g, 75.0 mmol, 5.00 equiv., 7.99 mL), palladium acetate (168 mg, 750 µmol, 0.05 equiv.), tetrapropylammonium bromide (7.99 g, 30.0 mmol, 2.00 equiv.), K_2CO_3 (6.22 g, 30.0 mmol, 3.00 equiv.), and DMF (75 mL) under argon. The reaction mixture was heated at 100 °C for 18 hours. Then the reaction was cooled to room temperature, diluted with dichloromethane, and washed with brine. The organic layer was dried with Na_2SO_4 and concentrated and purified by flash column chromatography on silica gel using dichloromethane/methanol 20:1, obtaining the title compound (3.86 mg, 9.31 mmol, 62%) as an orange solid. $\mathbf{R_f}$ = 0.4 (dichloromethane/methanol 20:1) **^{1}H NMR (400 MHz, CDCl$_3$, ppm):** δ = 8.67–8.58 (m, 4H, H_{Py}), 7.46–7.36 (m, 6H, H_{Py}, C*H*=CH), 6.81 (d, *J* = 16.2 Hz, 2H, CH=C*H*), 6.74 (d, *J* = 1.9 Hz, 2H, H_{Ar}), 6.64 (dd, J = 7.8, 1.9 Hz, 2H, H_{Ar}), 6.44 (d, J = 7.8 Hz, 2H, H_{Ar}), 3.67–3.55 (m, 2H, H_{PC}), 3.22–3.11 (m, 2H, H_{PC}), 3.08–2.93 (m, 4H, H_{PC}). **^{13}C NMR (100 MHz, CDCl$_3$, ppm):** δ = 150.3 (+, CH, 4C, C_{Py}), 145.0 (C_q, 2C, C_{Py}), 139.6 (C_q, 2C, C_{Ar}), 138.9 (C_q, 2C, C_{Ar}), 136.4 (C_q, 2C, C_{Ar}), 133.8 (+, 2C, C_{Ar}), 131.1 (+, 2C, *C*H=CH), 130.6 (+, 2C, C_{Ar}), 130.5 (+, 2C, C_{Ar}), 126.7 (+, 2C, CH=*C*H), 120.9 (+, CH, 4C, C_{Py}), 34.6 (–, 2C, CH_2), 33.3 (–, 2C, CH_2). **MS (EI, 70 eV, 20 °C, %):** m/z = 415/416 (100/32) $[M+H]^+$, 154 (87) 137 (56), 136 (67). **HRMS-EI (m/z):** $[M+H]^+$, calc. for $C_{30}H_{26}N_2$, 415.2169; found: 415.2383. **IR (ATR, ṽ)** = 3016 (w), 2928 (w), 1588 (vs), 1548 (m), 1490 (w), 1477 (w), 1438 (w), 1411 (s), 1341 (w), 1329 (w), 1306 (w), 1241 (w), 1220 (w), 1197 (w), 1142 (w), 989 (m), 972 (vs), 942 (w), 894 (w), 875 (s), 857 (s), 803 (vs), 783 (m), 741 (m), 721 (m), 656 (m), 628 (m), 565 (s), 534 (vs), 518 (vs), 480 (m), 470 (m) cm^{-1}.
https://dx.doi.org/10.14272/reaction/SA-FUHFF-UHFFFADPSC-AJVZTLNAQX-UHFFFADPSC-NUHFF-NQVUJ-NUHFF-ZZZ

4,16-Di(*N*-methyl-4'-pyridinium-(*E*)-vinyl)[2.2]paracyclophane iodide (pDMPCP; 13) :

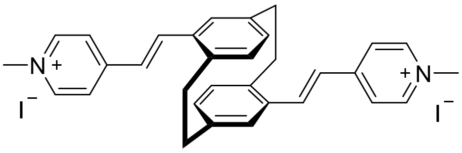

4,16-Di-((*E*)-4'-pyridylvinyl[2.2]paracyclophane (100 mg, 240 µmol, 1.00 equiv.) dissolved in acetonitrile 5 mL, then methyl iodide (200 µL) was added. The mixture was stirred at 40 °C for16 h, excluded of the light. The solvent was removed under reduced pressure to give a residue and then washed with dichloromethane and methanol to obtain 4,16-di(*N*-methyl-4'-pyridinium-(*E*)-vinyl)[2.2]paracyclophane iodide (125 mg, 179 µmol, 74%) as a yellow solid. **^{1}H NMR (400 MHz, DMSO-d6, ppm):** δ = 8.88 (d, *J* = 6.4 Hz, 4H, H_{Py}), 8.39 (d, *J* = 6.4 Hz, 4H, H_{Py}), 8.01 (d, *J* = 16.1 Hz, 2H, CH=CH), 7.31 (d, *J* = 15.9 Hz, 2H, CH=CH), 7.02 (s, 2H, H_{Ar}), 6.63–6.51 (m, 4H, H_{Ar}), 4.29 (s, 6H, CH_3), 3.81 (td, *J* = 11.5, 10.9, 6.1 Hz, 2H, H_{PC}), 3.23–2.87 (m, 6H, H_{PC}). **^{13}C NMR**

(100 MHz, DMSO-d6, ppm): δ = 153.2 (C_q, 2C, C_{Py}), 145.4 (+, 4C, C_{Py}), 141.3 (C_q, 2C, C_{Ar}), 140.3 (C_q, 2C, C_{Ar}), 138.8 (+, 2C, CH=CH), 136.1 (C_q, 2C, C_{Ar}), 134.3 (+, 2C, C_{Ar}), 132.7 (+, 2C, C_{Ar}), 131.5 (+, 2C, C_{Ar}), 124.4 (+, 2C, CH=CH), 124.2 (+, 4C, C_{Py}), 47.4 (+, 2C, CH_3), 34.6 (–, 2C, CH_2), 33.0 (–, 2C, CH_2). **MS (ESI, 70 eV, 20 °C, %):** m/z = 222/223/223 (100/32/5) $[M–2I]^{2+}$. **HRMS-ESI (m/z):** $[M–2I]^{2+}$, calc. for $C_{32}H_{32}N_2$, 222.1277/222.6294; found: 222.1273/222.6289. **IR (ATR, ṽ)** = 3020 (w), 2935 (w), 1638 (vs), 1615 (vs), 1587 (w), 1561 (w), 1517 (s), 1482 (w), 1466 (m), 1442 (w), 1326 (m), 1211 (w), 1183 (vs), 972 (s), 962 (vs), 874 (vs), 854 (w), 822 (vs), 657 (m), 552 (w), 521 (vs), 506 (vs) cm^{-1}.
https://dx.doi.org/10.14272/reaction/SA-FUHFF-UHFFFADPSC-VELYGFXWBW-UHFFFADPSC-NUHFF-LODAN-NUHFF-ZZZ

(*rac*)-4,15-Di(4'-pyridyl-(*E*)-vinyl)[2.2]paracyclophane (11):

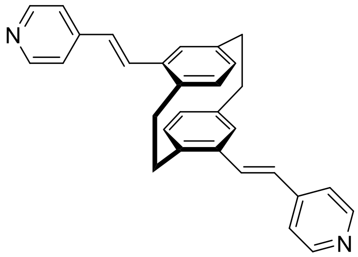

A vessel was charged with 4,15-dibromo[2.2]paracyclophane (732 mg, 2.00 mmol, 1.00 equiv.), 4-vinylpyridine (842 mg, 8.00 mmol, 4.00 equiv.), palladium acetate (22.0 mg, 99.0 µmol, 0.05 equiv.), tetrapropylammonium bromide (1.33 mg, 5.00 mmol, 2.50 equiv.), potassium acetate (1.38 mg, 10.0 mmol, 5.0 equiv.), and DMF (25 mL) under argon, and heated at 110 °C for 48 hours. Then the reaction was cooled to room temperature, diluted with CH_2Cl_2 (3 × 25 mL), and washed with brine. The organic layer was dried with Na_2SO_4 and concentrated. The residual liquid was purified by silica gel column chromatography to give 4,15-di((*E*)-4'-pyridinylvinyl)[2.2]paracyclophane as a light orange solid (531 mg, 1.28 mmol, 45%). R_f = 0.35 (dichloromethane /methanol 20:1). **^{1}H NMR (400 MHz, $CDCl_3$, ppm):** δ = 8.65–8.61 (m, 4H, H_{Py}), 7.42 (d, J = 6.2 Hz, 4H, H_{Py}), 7.38 (d, J = 16.2 Hz, 2H, CH=CH), 6.86 (d, J = 16.2 Hz, 2H, CH=CH), 6.80 (d, J = 1.9 Hz, 2H, H_{Ar}), 6.66 (d, J = 7.8 Hz, 2H, H_{Ar}), 6.47 (dd, J = 7.9, 1.9 Hz, 2H, H_{Ar}), 3.66–3.52 (m, 2H, H_{PC}), 3.25–3.12 (m, 2H, H_{PC}), 3.09–2.96 (m, 2H, H_{PC}), 2.92–2.81 (m, 2H, H_{PC}). **^{13}C NMR (100 MHz, $CDCl_3$, ppm):** δ = 150.3 (+, CH, 4C, C_{Py}), 145.0 (C_q, 2C, C_{Ar}), 140.1 (C_q, 2C, C_{Ar}), 138.7 (C_q, 2C, C_{Ar}), 136.8 (C_q, 2C, C_{Py}), 132.0 (+, CH, 2C, C_{Ar}), 131.6 (+, CH, 2C, C_{Ar}), 130.7 (+, 2C, CH=CH), 130.1 (+, CH, 2C, C_{Ar}), 126.5 (+, 2C, CH=CH), 120.9 (+, CH, 4C, C_{Py}), 35.0 (–, 2C, CH_2), 33.6 (–, 2C, CH_2). **MS (EI, 70 eV, 20 °C, %):** m/z = 415/416 (100/32) $[M+H]^+$, 193 (75) $[C_{30}H_{27}N_2]^+$ **HRMS-EI (m/z):** $[M+H]^+$, calc. for $C_{28}H_{28}N_2$, 415.2169; found: 415.2166. **IR (ATR, ṽ)** = 3021 (w), 2934 (w), 2922 (w), 1591 (vs), 1545 (w), 1487 (w), 1482 (w), 1472 (w), 1405 (m), 1215 (w), 1198 (w), 990 (w), 958 (vs), 912 (w), 891 (w), 871 (m), 850 (m), 805 (vs), 799 (vs), 737 (w), 720 (s), 687 (w), 650 (w), 632 (m), 603 (w), 558 (w), 547 (s), 531 (vs), 507 (vs), 484 (m), 469 (w), 458 (w), 441 (w), 424 (w), 404 (w), 378 (w) cm^{-1}.
https://dx.doi.org/10.14272/reaction/SA-FUHFF-UHFFFADPSC-IWOSJQVRUP-UHFFFADPSC-NUHFF-NURGD-NUHFF-ZZZ

(*rac*)-4,15-Di(*N*-Methyl-4'-pyridinium-(*E*)-vinyl)[2.2]paracyclophane iodide (pseudo-*meta*-DMVCP; 14):

A solution of a mixture of 4,15-di(4'-pyridyl-(*E*)-vinyl)[2.2]paracyclophane (84.0 mg, 232 µmol, 1.00 equiv.) and methyl iodide (3.21mmol, 200 µL) in acetonitrile (10 mL) was stirred at 40 °C for

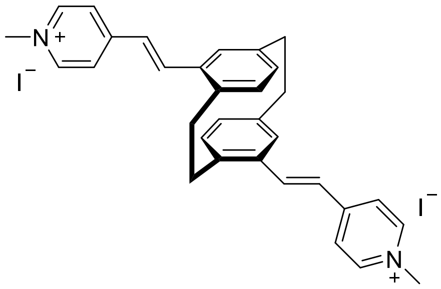

8 h protected from light. After the solution was cooled to room temperature, removed solvent under reduced pressure to give a residue, washed with water (20 mL), and dried in air to yield an orange solid (98.2 mg, 140 µmol, 69%). **^{1}H NMR (400 MHz, DMSO-d$_6$, ppm):** δ = 8.89 (d, *J* = 6.5 Hz, 4H, H_{Py}), 8.40 (d, *J* = 6.5 Hz, 4H, H_{Py}), 7.98 (d, *J* = 16.1 Hz, 2H, C*H*=CH), 7.36 (d, *J* = 16.0 Hz, 2H, C*H*=C*H*), 7.08 (d, *J* = 1.7 Hz, 2H, H_{Ar}), 6.65–6.52 (m, 4H, H_{Ar}), 4.30 (s, 6H, CH_3), 3.86–3.77 (m, 2H, H_{PC}), 3.24–3.12 (m, 2H, H_{PC}), 3.10–2.98 (m, 2H, H_{PC}), 2.94–2.81 (m, 2H, H_{PC}). **^{13}C NMR (101 MHz, DMSO-d$_6$, ppm):** δ = 153.2 (C_q, 2C, C_{Py}), 145.4 (+, CH, 4C, C_{Py}), 141.2 (C_q, 2C, C_{Ar}), 140.6 (C_q, 2C, C_{Ar}), 138.4 (+, 2C, *C*H=CH), 136.3 (C_q, 2C, C_{Ar}), 134.1 (+, CH, 2C, C_{Ar}), 132.5 (+, CH, 2C, C_{Ar}), 131.1 (+, CH, 2C, C_{Ar}), 124.2 (+, CH, 4C, C_{Py}), 124.2 (+, 2C, CH=*C*H), 47.4 (+, 2C, CH_3), 34.8 (–, 2C, CH_2), 33.7 (–, 2C, CH_2). **MS (ESI, 70 eV, 20 °C, %):** m/z = 222 (100) $[M-2I]^{2+}$. **HRMS-ESI (m/z):** $[M-2I]^{2+}$, calc. for $C_{32}H_{32}N_2$, 222.1277; found: 222.1277. **IR (ATR, ṽ)**: = 3019 (m), 3013 (m), 2924 (w), 1638 (s), 1608 (vs), 1587 (s), 1561 (m), 1516 (s), 1483 (w), 1466 (m), 1441 (w), 1323 (w), 1187 (vs), 975 (w), 962 (m), 887 (w), 877 (m), 824 (s), 730 (w), 708 (w), 517 (vs), 497 (w), 486 (w), 466 (w) cm^{-1}.
https://dx.doi.org/10.14272/reaction/SA-FUHFF-UHFFFADPSC-LDXRSQKGYP-UHFFFADPSC-NUHFF-LDXWS-NUHFF-ZZZ

4,5,12,13- And 4,7,12,15-tetrabromo[2.2]paracyclophane (15 and **16):**

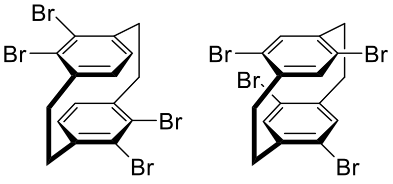

[2.2]Paracyclophane (5.00 g, 24.0 mmol, 1.00 equiv.) was slowly added in a 250 g flask to a mixture of bromine (46.5 mg, 15.0 mL, 291 mmol, 12.1 equiv.) and iodine (75.0 mg, 296 µmol, 0.012 equiv.). The solution was kept in the dark (vigorous evolution of hydrogen bromide!) and stirred for 7 d at room temperature in a flask wrapped with aluminum foil to exclude light. The reaction mixture was then decomposed by adding 300 ml of 20% aqueous sodium hydroxide. The precipitate is collected by filtration, washed with hot ethanol (3 × 50 mL), and dried in vacuo, as a mixture in equal amounts. The product mixture was extracted with dichloromethane (4 × 80 mL) to separate the isomers, yielding a residue of almost pure 4,5,12,13-tetrabromo[2.2]paracyclophane. A pure sample of 4,7,12,15-tetrabromo[2.2]paracyclophane was obtained by chromatography of 1 g of the mixture over 100 g of silica gel to yield a colorless solid (3.45 g, 6.59 mmol, 27%). And 4,5,12,13-tetrabromo[2.2]paracyclophane was combined to yield the final branch colorless solid (2.79 g, 5.33 mmol, 22%).

4,5,12,13-Tetrabromo[2.2]paracyclophane: R$_f$ = 0.55 (cyclohexane). **^{1}H NMR (400 MHz, CDCl$_3$, ppm)**: δ = 6.99 (s, 4H, H_{Ar}), 3.42–3.29 (m, 4H, H_{PC}), 3.16–3.04 (m, 4H, H_{PC}). **^{13}C NMR (100 MHz, CDCl$_3$, ppm):** δ = 140.7 (C_q, 4C, C_{Ar}), 129.3 (C_q, 4C, C_{Ar}), 128.7 (+, 4C, C_{Ar}), 34.6 (–, 4C, CH_2). **MS (EI, 70 eV, 20 °C, %):** m/z = 526/524/522 (19/28/19) $[M+H]^+$, 264/262/260/ (47/100/48) $[C_8H_6Br_2]^+$. **HRMS-EI (m/z):** $[M+H]^+$, calc. for $C_{16}H_{12}{}^{79}Br_2{}^{81}Br_2$, 523.7632; found: 523.7731. **IR (ATR, ṽ):** = 3456 (s), 3445 (s), 3414 (s), 3405 (s), 2934 (s), 1436 (vs), 1373 (vs), 1062 (vs), 837 (vs), 771 (s), 764 (s), 735 (vs), 707 (vs), 673 (vs), 523 (vs), 477 (s), 465 (s), 436 (s), 424 (s), 414 (s), 405 (s), 395 (s), 384 (s) cm^{-1}.

4,7,12,15-Tetrabromo[2.2]paracyclophane: R$_f$ = 0.5 (cyclohexane). **^{1}H NMR (400 MHz, $CDCl_3$, ppm):** δ = 7.20 (s, 4H, H_{Ar}), 3.30–3.17 (m, 4H, H_{PC}), 3.14–2.92 (m, 4H, H_{PC}). **^{13}C NMR (100 MHz, $CDCl_3$, ppm):** δ = 140.3 (C_q. 4C, C_{Ar}), 134.4 (+, 4C, C_{Ar}), 125.3 (C_q. 4C, C_{Ar}), 32.7 (–, 4C, CH_2). **MS (EI, 70 eV, 20 °C, %):** m/z = 526/524/522 (19/28/19) $[M+H]^+$, 264/262/260/ (48/100/49) $[C_8H_6Br_2]^+$. **HRMS-EI (m/z):** $[M+H]^+$, calc. for $C_{16}H_{12}{}^{79}Br_2{}^{81}Br_2$, 523.7632; found: 523.7731. **IR (ATR, ṽ)** = 3456 (s), 3445 (s), 3414 (s), 3405 (s), 2934 (s), 1436 (vs), 1373 (vs), 1062 (vs), 914 (s), 837 (vs), 771 (s), 764 (s), 735 (vs), 707 (vs), 673 (vs), 523 (vs), 477 (s), 465 (s), 436 (s), 424 (s), 414 (s), 405 (s), 395 (s), 384 (s) cm^{-1}.
https://dx.doi.org/10.14272/reaction/SA-FUHFF-UHFFFADPSC-OSLKNCBIIW-UHFFFADPSC-NUHFF-NUHFF-NUHFF-ZZZ

4,7,12,15-tetra(4'-pyridinyl)[2.2]paracyclophane (17) and 4,7,12-Tri(4'-pyridinyl)[2.2]paracyclophane (18):

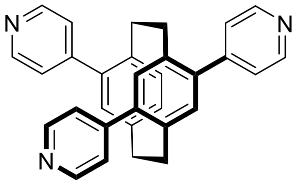

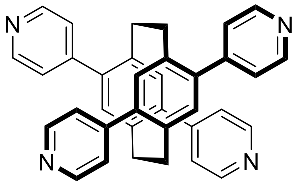

A vessel was charged with 4,7,12,15-tetrabromo[2.2]paracyclophane (500 mg, 954 µmol, 1.00 equiv.), pyridine-4-boronic acid (939 mg, 7.64 mmol, 8.00 equiv.), palladium-tetrakis(triphenylphosphine) (70.0 mg, 6.00 µmol, 0.06 equiv.), potassium phosphate (1.06 g, 50.0 µmol, 5.23 equiv.), dioxane (16 mL) and water (8 mL) under argon atmosphere. The mixture was heated under reflux for 16 h. The reaction mixture was then cooled to room temperature, and extracted with DCM (3 × 50 mL), the extracts were dried with $MgSO_4$ and evaporated under reduced pressure. The crude solid was purified by flash column chromatography on silica gel using dichloromethane /methanol 20:1, then obtain the compounds 4,7,12-tri(4'-pyridinyl)[2.2]paracyclophane (105 mg. 239 µmol, 25%) and 4,7,12,15-tetra(4'-pyridinyl)[2.2]paracyclophane (102 mg, 197 µmol, 21%) respectively as colorless solid.

4,7,12,15-Tetra(4'-pyridinyl)[2.2]paracyclophane (17): R$_f$ = 0.2 (dichloromethane /methanol 20:1).-**^{1}H NMR (400 MHz, $CDCl_3$, ppm):** δ = 8.73–8.61 (m, 8H, H_{Py}), 7.28–7.26 (m, 8H, H_{Py}), 6.88 (s, 4H, H_{Ar}), 3.67–3.51 (m, 4H, H_{PC}), 2.97–2.81 (m, 4H, H_{PC}).-**^{13}C NMR (100 MHz, $CDCl_3$, ppm):** δ = 150.4 (+, 8C, C_{Py}), 147.4 (q, 4C, C_{Py}), 138.8 (q, 4C, C_{Ar}), 137.8 (q, 4C, C_{Ar}), 132.59 (+, 4C, C_{Ar}), 123.38 (+, 8C, C_{Py}), 33.29 (–, 4C, CH_2).-**MS (70 eV, ESI), m/z (%):** 517/518 (100/39) $[M+H]^+$.-**HRMS-EI (m/z)**: calc. for $C_{36}H_{29}N_4$, 517.2387; found: 517.2390.-**IR (ATR, ṽ)** = 1589 (vs), 1543 (m), 1411 (s), 826 (vs), 812 (vs), 727 (m), 718 (m), 693 (vs), 671 (s), 541 (s), 528 (s), 484 (vs), 469 (s), 458 (s), 419 (m) cm^{-1}.

4,7,12-Tri(4'-pyridinyl)[2.2]paracyclophane (18): R$_f$ = 0.24 (dichloromethane /methanol 20:1). **^{1}H NMR (400 MHz, $CDCl_3$, ppm):** δ = 8.81–8.75 (m, 2H, H_{Py}), 8.72–8.67 (m, 2H, H_{Py}), 8.63–8.56 (m, 2H, H_{Py}), 7.55–7.49 (m, 2H, H_{Py}), 7.34–7.29 (m, 2H, H_{Py}), 7.18–7.11 (m, 2H, H_{Py}), 6.88–6.80 (m, 2H, H_{Ar}), 6.77 (dd, J = 8.1, 6.6 Hz, 3H, H_{Ar}), 3.62 (ddd, J = 13.6, 10.1, 1.7 Hz, 1H, H_{PC}), 3.55–3.45 (m, 1H, H_{PC}), 3.36–3.25 (m, 2H, H_{PC}), 3.08 (ddd, J = 13.7, 10.5, 6.4 Hz, 1H, H_{PC}), 2.99–2.92 (m, 1H, H_{PC}), 2.72–2.53 (m, 2H, H_{PC}). **^{13}C NMR (100 MHz, $CDCl_3$, ppm):** δ = 150.3 (+, 2C, C_{Py}), 150.2 (+, 2C, C_{Py}), 150.0 (+, 2C, C_{Py}), 148.2 (C_q, C_{Py}), 147.7 (C_q, C_{Py}), 147.5 (C_q, C_{Py}), 140.2 (C_q, C_{Ar}), 139.8 (C_q, C_{Ar}), 138.4 (C_q, C_{Ar}), 138.1 (C_q, C_{Ar}), 138.0 (C_q, C_{Ar}), 137.8 (C_q, C_{Ar}), 137.4 (C_q, C_{Ar}), 135.0 (+, CH, C_{Ar}), 133.1 (C_q, C_{Ar}), 132.5 (C_q, C_{Ar}), 132.5 (C_q, C_{Ar}), 129.7 (C_q, C_{Ar}), 124.1 (+,

2C, CH, C_{Py}), 123.6 (+, 2C, CH, C_{Py}), 123.5 (+, 2C, CH, C_{Py}), 34.5 (–, CH_2), 33.8 (–, CH_2), 33.3 (–, CH_2), 33.2 (–, CH_2). **MS (70 eV, ESI), m/z (%):** 440/441 (100/44) $[M+H]^+$. **HRMS-EI (m/z)**: calc. for $C_{31}H_{25}N_3$, 440.2122; found: 440.2114. **IR (ATR, ṽ):** = 3020 (w), 2958 (w), 2936 (w), 2917 (w), 2871 (w), 2863 (w), 1591 (vs), 1541 (s), 1475 (m), 1456 (w), 1441 (w), 1409 (s), 1401 (m), 1380 (w), 1215 (w), 1181 (w), 1065 (w), 992 (m), 882 (m), 827 (vs), 820 (vs), 803 (s), 756 (m), 728 (m), 720 (m), 710 (s), 688 (m), 680 (s), 669 (m), 653 (w), 639 (s), 623 (s), 591 (w), 571 (m), 562 (m), 548 (s), 524 (m), 511 (w), 496 (vs), 476 (vs), 450 (s), 436 (m), 426 (m), 418 (m), 411 (m), 398 (w), 388 (w), 378 (m) cm^{-1}.
https://dx.doi.org/10.14272/reaction/SA-FUHFF-UHFFFADPSC-DKUQPLPGXQ-UHFFFADPSC-NUHFF-NUHFF-NUHFF-ZZZ.1

4,7,12,15-Tetra(*N*-methyl-4'-pyridinium)[2.2]paracyclophane iodide (QMPCP; 22):

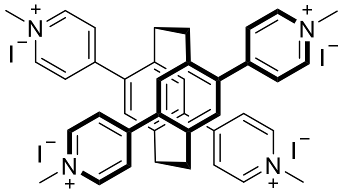

4,7,12,15-Tetra-(4'-pyridine)[2.2]paracyclophane (15.0 mg, 29 µmol, 1.00 equiv.) dissolved in acetonitrile 5 mL, then methyl iodide (100 µL) was added. The mixture was stirred at 40 °C for 12 h excluding the light. The solvent was removed under reduced pressure to give a residue and then recrystallized from methanol to obtain 4,7,12,15-tetra-(*N*-methyl-4'-pyridinium)[2.2]paracyclophane iodide (25.1 mg, 23.1 µmol, 79%) as a yellow solid. **^{1}H NMR (400 MHz, DMSO-d6, ppm):** δ = 9.09 (d, *J* = 6.3 Hz, 8H, H_{Py}), 8.19 (d, *J* = 6.2 Hz, 8H, H_{Py}), 7.52 (s, 4H, H_{Ar}), 4.45 (s, 12H, CH_3), 3.56–3.68 (m, 4H, H_{PC}), 3.27–3.19 (m, 4H, H_{PC}). **^{13}C NMR (100 MHz, DMSO-d6, ppm):** δ = 152.1 (C_q, 4C, C_{Py}), 146.2 (+, 8C, C_{Py}), 140.5 (C_q, 4C, C_{Ar}), 137.4 (C_q, 4C, C_{Ar}), 133.5 (+, 4C, C_{Ar}), 127.0 (+, 8C, C_{Py}), 47.8 (+, 4C, CH_3), 33.3 (–, 4C, CH_2). **HRMS-ESI (m/z):** $[M–2I]^{2+}$, calc. for $C_{48}H_{48}N_4I_2$, 467.0979/467.5996; found: 467.0977/467.5992 (100/54). $[M–3I]^{3+}$, calc. for $C_{48}H_{48}N_4I_3$, 269.0969/269.4314; found: 269.0967/269.4311 (65/7). $[M–4I]^{4+}$, calc. for $C_{48}H_{48}N_4$, 170.0964/170.3473; found: 170.0964/1703472 (34/8). **IR (ATR, ṽ):** = 3444 (m), 3431 (m), 3415 (m), 3410 (m), 3401 (m), 3393 (m), 3381 (m), 3370 (m), 3343 (m), 3029 (m), 3010 (m), 1635 (vs), 1561 (m), 1519 (s), 1459 (m), 1436 (m), 1191 (s), 1044 (m), 830 (vs), 693 (m), 555 (m), 537 (s), 526 (s), 506 (m), 486 (s), 469 (vs), 446 (vs), 441 (vs), 419 (vs), 414 (vs), 405 (vs), 398 (vs), 388 (vs), 377 (vs) cm^{-1}.
https://dx.doi.org/10.14272/reaction/SA-FUHFF-UHFFFADPSC-VVMGLLAOFL-UHFFFADPSC-NUHFF-JUHFF-NUHFF-ZZZ.1

4,7,12-Tri(*N*-methyl-4'-pyridinium)[2.2]paracyclophane iodide (TMPCP; 23):

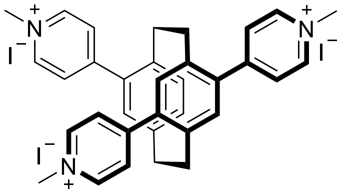

4,7,12-Tri-(4'-pyridinyl)[2.2]paracyclophane (22.0 mg, 50.1 µmol, 1.00 equiv) dissolved in acetonitrile 5 mL, then methyl iodide (100 µL) was added. The mixture was stirred at 40 °C for 16 h, excluding the light. The solvent was removed under reduced pressure to give a residue and then recrystallized from methanol to obtain 4-bromo-7,12,15-tri-(*N*-methyl-4'-pyridinyl)[2.2]paracyclophane iodide (65.2 mg, 75.1 µmol, 89%) as a yellow solid. **^{1}H NMR (400 MHz, DMSO-d6, ppm):** δ = 9.13 (d, *J* = 6.4 Hz, 2H, H_{Py}), 9.09 (d, *J* = 6.5 Hz, 2H, H_{Py}), 8.99 (d, *J* = 6.5 Hz, 2H, H_{Py}), 8.48 (d, *J* = 6.3 Hz, 2H, H_{Py}), 8.13 (d, *J* = 6.5 Hz, 2H, H_{Py}), 7.99 (d, *J* = 6.4 Hz, 2H, H_{Py}), 7.50 (s, 1H, H_{Ar}), 7.26 (dd, *J* = 7.8, 1.8 Hz, 1H, H_{Ar}), 7.24 (s, 1H, H_{Ar}), 7.14 (d, *J* = 1.8 Hz, 1H, H_{Ar}), 6.79 (d, *J* = 7.9 Hz, 1H, H_{Ar}), 4.45 (s, 3H, CH_3), 4.44 (s, 3H, CH_3), 4.39 (s, 3H, CH_3), 3.59 (dd, *J* = 13.1, 9.8 Hz, 1H, H_{PC}), 3.45–3.33 (m, 4H, H_{PC}), 3.06–2.86 (m, 2H, H_{PC}), 2.73–2.85 (m, 1H, H_{PC}). **^{13}C NMR (100 MHz, DMSO-d6, ppm):** δ

= 153.8 (C_q, C_{Py}), 153.6 (C_q, C_{Py}), 153.1 (C_q, C_{Py}), 146.3 (+, 2C, CH, C_{Py}), 146.2 (+, 2C, CH, C_{Py}), 146.0 (+, 2C, CH, C_{Py}), 140.8 (C_q, C_{Ar}), 140.6 (C_q, C_{Ar}), 130.0 (C_q, C_{Ar}), 139.5 (C_q, C_{Ar}), 139.4 (C_q, C_{Ar}), 136.7 (C_q, C_{Ar}), 136.1 (+, CH, C_{Ar}), 135.7 (+, CH, C_{Ar}), 135.3 (C_q, C_{Ar}), 133.9 (+, CH, C_{Ar}), 133.8 (+, CH, C_{Ar}), 130.1 (+, CH, C_{Ar}), 127.8 (+, 2C, CH, C_{Py}), 127.1 (+, 2C, CH, C_{Py}), 126.8 (+, 2C, CH, C_{Py}), 47.9 (+, CH_3), 47.8 (+, CH_3), 47.6 (+, CH_3), 33.9 (–, CH_2), 33.6 (–, CH_2), 33.5 (–, CH_2), 33.1 (–, CH_2). **MS (ESI, 70 eV, 20 °C, %) m/z** = 306 (100) $[M–2I]^{2+}$, 378 (20) $[M–I]^{+}$. **HRMS-ESI (m/z):** $[M–2I]^{2+}$, calc. for $C_{34}H_{34}IN_3$, 305.5893/306.0910; found: 305.5892/306.0908. $[M–I]^{+}$, calc. for $C_{34}H_{34}I_2N_3$, 738.0837/739.0871; found: 738.0836/739.0870. **IR (ATR, ṽ):** = 3398 (s), 3378 (s), 3374 (s), 3367 (s), 3359 (s), 3347 (m), 3339 (m), 1635 (vs), 1561 (m), 1514 (s), 1458 (s), 1193 (s), 836 (vs), 816 (s), 727 (s), 623 (m), 599 (s), 581 (s), 569 (s), 547 (vs), 524 (vs), 494 (vs), 466 (vs), 449 (vs), 441 (vs), 424 (vs), 415 (vs), 405 (vs), 395 (vs), 388 (vs) cm^{-1}.
https://dx.doi.org/10.14272/reaction/SA-FUHFF-UHFFFADPSC-VVMGLLAOFL-UHFFFADPSC-NUHFF-JUHFF-NUHFF-ZZZ

4,7,12,15-Tetra(4'-pyridyl-(*E*)-vinyl)[2.2]paracyclophane (19):

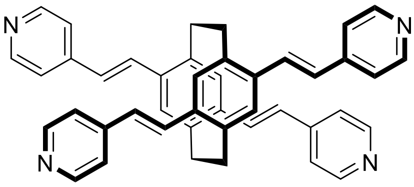

A vessel was charged with 4,7,12,15-tetrabromo[2.2]paracyclophane (1.00 g, 1.91 mmol, 1.00 equiv.), 4-vinylpyridine (3.01 mg, 28.6 mmol, 3.05 mL, 15.0 equiv.), palladium acetate (21.0 mg, 93.5 µmol, 0.05 equiv.), tetrapropylammonium bromide (1.27 g, 4.78 mmol, 2.50 equiv.), potassium carbonate (1.32 g, 9.55 mmol, 5.00 equiv.), and DMF (12 mL) under argon. The reaction mixture was heated at 110 °C for 18 hours. Then the reaction was cooled to room temperature, diluted with CH_2Cl_2, and washed with brine. The organic layer was dried with $MgSO_4$ and concentrated, and purified by flash column chromatography on silica gel using dichloromethane/methanol 20:1, obtaining the title compound (549 mg, 885 µmol, 46%) as an orange solid. $\mathbf{R_f}$ = 0.3 (dichloromethane/methanol 20:1). **^{1}H NMR (400 MHz, $CDCl_3$, ppm):** δ = 8.67–8.61 (m, 8H, H_{Py}), 7.41 (d, J = 16.2 Hz, 4H, CH=CH), 7.36–7.27 (m, 8H, H_{Py}), 7.00 (s, 4H, H_{Ar}), 6.81 (d, J = 16.1 Hz, 4H, CH=CH), 3.72–3.60 (m, 4H, H_{PC}), 3.01–2.88 (m, 4H, H_{PC}). **^{13}C NMR (100 MHz, $CDCl_3$, ppm):** δ = 150.5 (+, CH, 8C, C_{Py}), 144.4 (C_q, 4C, C_{Py}), 138.6 (C_q, 4C, C_{Ar}), 136.6 (C_q, 4C, C_{Ar}), 129.1 (+, 4C, CH=CH), 129.0 (+, 4C, C_{Ar}), 126.7 (+, 4C, CH=CH), 120.8 (+, CH, 8C, C_{Py}), 33.1 (–, 2C, CH_2), 1.0 (–, 2C, CH_2). **HRMS-ESI (m/z):** $[M+H]^{+}$, calc. for $C_{44}H_{36}N_4$, 621.3013/622.3047; found: 621.3010/622.3043 (45/100). **IR (ATR, ṽ)** = 3377 (w), 3369 (w), 3356 (w), 3349 (w), 3340 (w), 3332 (w), 3325 (w), 3315 (w), 3299 (w), 3288 (w), 3284 (w), 3269 (w), 3258 (w), 3245 (w), 3216 (w), 3033 (w), 2934 (w), 1589 (vs), 1547 (m), 1459 (w), 1412 (m), 1324 (w), 1217 (w), 965 (s), 833 (m), 799 (s), 592 (m), 524 (s), 514 (m), 497 (w) cm^{-1}.
https://dx.doi.org/10.14272/reaction/SA-FUHFF-UHFFFADPSC-SYMSPZWKXL-UHFFFADPSC-NUHFF-NMPTO-NUHFF-ZZZ.1

4,7,12,15-Tetra(3'-pyridyl-(*E*)-vinyl)[2.2]paracyclophane (20):

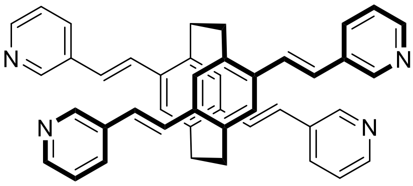

A vessel was charged with 4,7,12,15-tetrabromo[2.2]paracyclophane (200 mg, 382 µmol, 1.00 equiv.), 3-vinylpyridine (241 mg, 2.29 mmol, 6.00 equiv.), palladium acetate (4.30 mg, 19.2 µmol, 0.05 equiv.), tetrapropylammonium bromide (254 mg, 955 µmol, 2.5 equiv.), potassium carbonate (264 mg, 1.91 mmol, 5.00 equiv.), and DMF (8 mL) under argon.

The reaction mixture was heated at 110 °C for 16 hours. Then the reaction was cooled to room temperature, diluted with CH_2Cl_2, and washed with brine. The organic layer was dried with $MgSO_4$ and concentrated, and purified by flash column chromatography on silica gel using dichloromethane/methanol 20:1, obtaining the title compound (203 mg, 327 µmol, 86%) as an orange solid. **R$_f$** = 0.4 (dichloromethane/methanol 20:1). **^{1}H NMR (400 MHz, CDCl$_3$, ppm):** δ = 8.71 (d, *J* = 2.3 Hz, 4H, H_{Py}), 8.55 (dd, *J* = 4.8, 1.6 Hz, 4H, H_{Py}), 7.75 (dt, *J* = 7.9, 2.0 Hz, 4H, H_{Py}), 7.37–7.32 (m, 4H, H_{Py}), 7.28 (d, *J* = 16.5 Hz, 4H, CH=CH), 7.00 (s, 4H, H_{Ar}), 6.87 (d, *J* = 16.2 Hz, 4H, CH=CH), 3.63 (q, *J* = 5.7 Hz, 4H, H_{PC}), 2.98–2.87 (m, 4H, H_{PC}). **^{13}C NMR (100 MHz, CDCl$_3$, ppm):** δ = 148.8 (+, 4C, C_{Py}), 148.2 (+, 4C, C_{Py}), 138.2 (C_q, 4C, C_{Ar}), 136.7 (C_q, 4C, C_{Ar}), 133.1 (+, 4C, C_{Py}), 133.0 (C_q, 4C, C_{Ar}), 128.5 (+, 4C, C_{Ar}), 127.1 (+, 4C, CH=CH), 125.3 (+, 4C, CH=CH), 123.8 (+, 4C, C_{Py}), 33.1 (–, 4C, CH_2). **HRMS-ESI (m/z):** [M+H]$^+$, calc. for $C_{44}H_{36}N_4$, 621.3013/622.3047; found: 621.3032/622.3001 (48/100). **IR (ATR, ṽ)** = 2931 (w), 1564 (w), 1485 (w), 1472 (w), 1460 (w), 1412 (m), 1323 (w), 1306 (w), 1238 (w), 1215 (w), 1183 (w), 1122 (w), 1099 (w), 1021 (m), 956 (vs), 904 (m), 873 (w), 858 (w), 846 (w), 826 (m), 790 (s), 758 (w), 700 (vs), 633 (m), 620 (m), 603 (m), 575 (w), 569 (w), 550 (w), 524 (w), 511 (w), 497 (m), 462 (w), 448 (w), 424 (w), 414 (w), 397 (m), 384 (w) cm^{-1}.
https://dx.doi.org/10.14272/reaction/SA-FUHFF-UHFFFADPSC-KEZSCKXKKQ-UHFFFADPSC-NUHFF-NDFLH-NUHFF-ZZZ.1

4,7,12,15-Tetra(2'-pyridyl-(*E*)-vinyl)[2.2]paracyclophane (21):

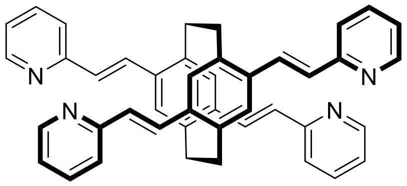

A vessel was charged with 4,7,12,15-tetrabromo[2.2]paracyclophan (500 mg, 961 µmol, 1.00 equiv.), 2-vinylpyridine (750 mg, 7.20 mmol, 750 µL), palladium acetate (11.0 mg, 49.0 µmol, 0.05 equiv.), tetrapropylammonium bromide (635 mg, 2.40 mmol, 2.50 equiv.), potassium carbonate (660 mg, 4.80 mmol, 5.00 equiv.), and DMF (12 mL) under argon. The reaction mixture was heated at 110 °C for 17 hours. Then the reaction was cooled to room temperature, diluted with CH_2Cl_2, and washed with brine. The organic layer was dried with $MgSO_4$ and concentrated. The crude product was purified via flash column chromatography on silica gel using dichloromethane/methanol 20:1, obtaining the title compound (123 mg, 198 µmol, 21%) as an orange solid. **R$_f$** = 0.45 (dichloromethane/methanol 20:1). **^{1}H NMR (400 MHz, CDCl$_3$, ppm):** δ = 8.68 (ddd, *J* = 4.8, 1.9, 0.9 Hz, 4H, H_{Py}), 7.82 (d, *J* = 15.8 Hz, 4H, CH=CH), 7.66 (td, *J* = 7.6, 1.8 Hz, 4H, H_{Py}), 7.22 (dt, *J* = 7.9, 1.1 Hz, 4H, H_{Py}), 7.18 (ddd, *J* = 7.5, 4.8, 1.1 Hz, 4H, H_{Py}), 7.09 (s, 4H, H_{Ar}), 6.99 (d, *J* = 15.8 Hz, 4H, CH=CH), 3.77–3.64 (m, 4H, H_{PC}), 3.03–2.90 (m, 4H, H_{PC}). **^{13}C NMR (100 MHz, CDCl$_3$, ppm):** δ = 155.9 (C_q, 4C, C_{Py}), 149.9 (+, 4C, C_{Py}), 139.0 (C_q, 4C, C_{Ar}), 136.9 (C_q, 4C, C_{Ar}), 136.5 (+, 4C, C_{Py}), 129.3 (+, 4C, CH=CH), 128.9 (+, 4C, C_{Ar}), 128.1 (+, 4C, CH=CH), 122.9 (+, 4C, C_{Py}), 122.0 (+, 4C, C_{Py}), 33.2 (–, 4C, CH_2). **HRMS-ESI (m/z):** [M+H]$^+$, calc. for $C_{44}H_{36}N_4$, 621.3013/622.3047; found: 621.3032/622.3001 (48/100). **IR (ATR, ṽ)** = 2922 (m), 1581 (vs), 1558 (s), 1483 (w), 1466 (vs), 1426 (vs), 1299 (w), 1203 (m), 1147 (s), 1091 (w), 1048 (w), 990 (w), 963 (vs), 907 (m), 887 (m), 860 (m), 829 (m), 771 (vs), 739 (vs), 715 (s), 676 (w), 635 (w), 612 (s), 550 (w), 526 (m), 500 (m), 479 (m), 456 (m), 446 (m), 431 (w), 426 (w), 401 (vs), 390 (w) cm^{-1}.
https://dx.doi.org/10.14272/reaction/SA-FUHFF-UHFFFADPSC-MRAPWJUTWD-UHFFFADPSC-NUHFF-NXLQL-NUHFF-ZZZ.1

3-Vinylpyridine:

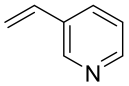

To a solution of methyl triphenylphosphonium bromide (1.78 g, 5.00 mmol, 1.00 equiv.) in THF (25 mL) at –78 °C was added LDA (536 mg, 5.00 mmol, 1.00 equiv.). Then 3-pyridinecarboxaldehyde (537 mg, 570 µL, 10.0 mmol, 1.00 equiv.) was added dropwise, and the resulting mixture was allowed to warm up to room temperature for 2 h. The reaction was quenched with water (50 mL) and extracted with cyclohexane (2 × 50 mL). The combined organic extracts were dried over $MgSO_4$, filtered, and concentrated in vacuo. The crude product was purified via flash column chromatography on silica gel using cyclohexane/ethyl acetate 4:3, obtaining the title compound (251 mg, 2.39 mmol, 45%) as colorless liquid as colorless liquid, but due to toxicity and stench, it will be used directly in next step in a small amount of solution. **R*f*** = 0.4 (cyclohexane/ethyl acetate 4:3). **^{1}H NMR (400 MHz, $CDCl_3$, ppm):** δ = 8.59 (d, *J* = 2.3 Hz, 1H, H_{Py}), 8.46 (dd, *J* = 4.8, 1.7 Hz, 1H, H_{Py}), 7.69 (dt, *J* = 7.9, 2.0 Hz, 1H, H_{Py}), 7.26–7.18 (m, 1H, H_{Py}), 6.67 (dd, *J* = 17.7, 11.0 Hz, 1H, CH=CH), 5.80 (d, *J* = 17.7 Hz, 1H, CH=CH), 5.35 (d, *J* = 11.0 Hz, 1H, CH=CH). **^{13}C NMR (100 MHz, $CDCl_3$, ppm):** δ = 148.9 (+, CH, C_{Py}), 148.3 (+, CH, C_{Py}), 133.5 (+, CH=CH2), 133.0 (C_q, C_{Py}), 132.6 (+, CH, C_{Py}), 123.4 (+, CH, C_{Py}), 116.2 (–, CH_2). **MS (EI, 70 eV, 20 °C, %):** m/z = 105/104 (100/78) $[M]^+$. **HRMS-EI (m/z):** $[M]^+$, calc. for C_7H_7N, 105.0578; found: 105.0781. **IR (ATR, ṽ)** = 1480 (m), 1473 (m), 1411 (m), 1400 (s), 1024 (s), 989 (vs), 914 (vs), 813 (vs), 713 (vs), 662 (m), 628 (s), 397 (s) cm^{-1}.
https://dx.doi.org/10.14272/reaction/SA-FUHFF-UHFFFADPSC-DPZYLEIWHT-UHFFFADPSC-NUHFF-NUHFF-NUHFF-ZZZ

4,7,12,15-Tetra(*N*-Methyl-4'-pyridinium-(*E*)-vinyl)[2.2]paracyclophane iodide (QMVCPp; 24):

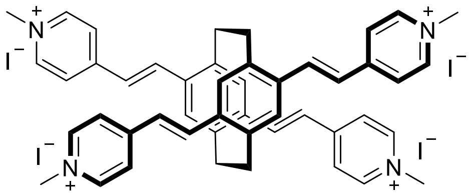

4,7,12,15-Tetra-(4'-pyridyl-(*E*)-vinyl)[2.2]paracyclophane (150 mg, 242 µmol, 1.00 equiv.) dissolved in acetonitrile 6 mL, then methyl iodide (150 µL) was added. The mixture was stirred at 40 °C for 14 h excluding the light. The solvent was removed under reduced pressure to give a residue and then recrystallized from methanol to obtain 4,7,12,15-tetra-(*N*-methyl-4'-pyridinium-(*E*)-vinyl[2.2]paracyclophane iodide (280 mg, 236 µmol, 97%) as a red solid. **^{1}H NMR (400 MHz, DMSO-d$_6$, ppm):** δ = 8.89 (d, *J* = 6.5 Hz, 8H, H_{Py}), 8.21 (d, *J* = 6.5 Hz, 8H, H_{Py}), 7.99 (d, *J* = 16.1 Hz, 4H, H_{Ar}), 7.18 (s, d, *J* = 16.6 Hz, 8H, H_{Ar}), 4.34 (s, 12H, CH_3), 3.98–3.93 (m, 4H, H_{PC}), 3.03–2.98 (m, 4H, H_{PC}). **^{13}C NMR (100 MHz, DMSO-d6, ppm):** δ = 152.6 (C_q, 4C, C_{Py}), 145.4 (+, 4C, C_{Py}), 141.4 (C_q, 4C, C_{Ar}), 137.5 (C_q, 4C, C_{Ar}), 136.5 (+, 4C, CH=CH), 131.0 (+, 4C, C_{Ar}), 125.1 (+, 4C, CH=CH), 124.5 (+, 4C, C_{Py}), 47.5 (+, 12C, CH_3), 33.1 (–, 4C, CH_2). **HRMS-ESI (m/z):** $[M–4I]^{4+}$, calc. for $C_{48}H_{48}N_4^{4+}$, 170.0964, found: 170.0965 (13); $[M–3I]^{3+}$, calc. for $C_{48}H_{48}N_4I^{3+}$, 269.0969/269.4314, found: 269.0969/269.4313 (24/12); $[M–2I]^{2+}$, calc. for $C_{48}H_{48}N_4I_2^{2+}$, 467.0979/467.5996, found: 467.0981/467.5997 (81/40); $[M–I]^+$, calc. for $C_{48}H_{48}N_4I_3^+$, 1061.1008, found: 1061.1010(4). **IR (ATR, ṽ)** = 3465 (m), 3439 (m), 3427 (m), 3391 (m), 1638 (s), 1604 (vs), 1562 (s), 1514 (s), 1466 (s), 1330 (m), 1320 (m), 1210 (m), 1190 (vs), 1169 (s), 975 (s), 965 (m), 880 (m), 837 (s), 561 (m), 543 (m), 516 (vs), 477 (m), 465 (m), 395 (m), 385 (s), 377 (s) cm^{-1}.
https://dx.doi.org/10.14272/reaction/SA-FUHFF-UHFFFADPSC-NBCOVCGBTU-UHFFFADPSC-NUHFF-JQLHQ-NUHFF-ZZZ

4,7,12,15-Tetra(*N*-methyl-3'-pyridinium-(*E*)-vinyl)[2.2]paracyclophane iodide (QMVCPm; 25):

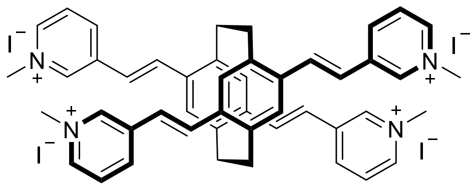

4,7,12,15-Tetra-((*E*)-vinyl-3'-pyridyl)[2.2]paracyclophane (62.0 mg, 99.9 µmol, 1.00 equiv.) dissolved in acetonitrile 6 mL, then methyl iodide (2.00 µL) was added. The mixture was stirred at 40 °C for 14 h excluding the light. The solvent was removed under reduced pressure to give a residue and then recrystallized from methanol to obtain 4,7,12,15-tetra-(*N*-methyl-3'-pyridinium-(*E*)-vinyl)[2.2]paracyclophane iodide (81.1 mg, 68.1 µmol, 68%) as an orange solid. **^{1}H NMR (400 MHz, DMSO-d_6, ppm):** δ = 9.36 (s, 4H, H_{Py}), 8.88 (d, *J* = 6.0 Hz, 4H, H_{Py}), 8.61 (d, *J* = 8.2 Hz, 4H, H_{Py}), 8.14 (dd, *J* = 8.2, 5.9 Hz, 4H, H_{Py}), 7.73 (d, *J* = 16.2 Hz, 4H, CH=CH), 7.11 (d, *J* = 16.3 Hz, 8H, CH=CH), 7.11 (s, 4H, H_{Ar}), 4.42 (s, 12H, CH_3), 3.85 (q, *J* = 6.7 Hz, 4H, H_{PC}), 3.02 (h, *J* = 8.5 Hz, 4H, H_{PC}). **^{13}C NMR (100 MHz, DMSO-d6, ppm):** δ = 143.7 (+, 4C, C_{Py}), 143.4 (+, 4C, C_{Py}), 141.9 (+, 4C, C_{Py}), 140.1 (C_q, 4C, C_{Py}), 137.7 (C_q, 4C, C_{Ar}), 137.1 (C_q, 4C, C_{Ar}), 131.7 (+, 4C, CH=CH), 129.9 (+, 4C, C_{Ar}), 127.9 (+, 4C, C_{Py}), 122.6 (+, 4C, CH=CH), 48.6 (+, 4C, CH_3), 33.0 (–, 4C, CH_2). **HRMS-ESI (m/z):** $[M–4I]^{4+}$, calc. for $C_{48}H_{48}N^{4+}$, 170.0964/170.3473; found: 170.0964/1703472 (34/8); $[M–3I]^{3+}$, calc. for $C_{48}H_{48}N_4I^{3+}$, 269.0969/269.4314; found: 269.0967/269.4311 (65/7); $[M–2I]^{2+}$, calc. for $C_{48}H_{48}N_4I_2^{2+}$, 467.0979/467.5996; found: 467.0977/467.5992 (100/54). **IR (ATR, ṽ)** = 3463 (w), 3427 (m), 3414 (m), 3023 (w), 3014 (w), 1615 (s), 1579 (m), 1504 (s), 1466 (w), 1298 (m), 953 (vs), 799 (m), 721 (w), 667 (vs), 533 (m), 473 (s), 443 (m), 419 (s), 398 (s), 388 (m), 378 (m) cm^{-1}.

https://dx.doi.org/10.14272/reaction/SA-FUHFF-UHFFFADPSC-JSESGCFTBC-UHFFFADPSC-NUHFF-JGOYP-NUHFF-ZZZ

4-(*E*)-Azophenyl[2.2]paracyclophane:

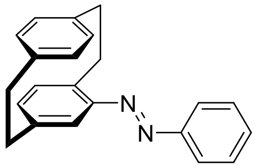

To a solution of 4-amino[2,2]paracyclophane (223 mg, 1.00 mmol, 1 equiv.) in acetic acid (10 mL), nitrosobenzene (214 mg, 2.00 mmol, 2 equiv.) was added and the reaction mixture was stirred for 48 hours at room temperature. The reaction mixture was extracted with dichloromethane, washed with water and brine, dried over $MgSO_4$, filtered, and concentrated under reduced pressure. The crude product was purified via flash column chromatography on silica gel using cyclohexane/ethyl acetate 20:1, obtaining the title compound (98 mg, 0.31 mmol, 31%) as a yellow solid. **R_f** = 0.4 (cyclohexane/ethyl acetate 20:1). **^{1}H NMR (400 MHz, $CDCl_3$, ppm):** δ = 7.93–7.85 (m, 2H, H_{Ar}), 7.49 (dd, *J* = 8.3, 6.7 Hz, 2H, H_{Ar}), 7.46–7.38 (m, 1H, H_{Ar}), 6.64–6.47 (m, 5H, H_{Ar}), 6.29 (qd, *J* = 7.8, 1.8 Hz, 2H, H_{Ar}), 4.16 (ddd, *J* = 12.8, 10.2, 2.5 Hz, 1H, H_{PC}), 3.20 (ddd, *J* = 12.9, 10.3, 2.4 Hz, 1H, H_{PC}), 3.12–2.97 (m, 5H, H_{PC}), 2.89 (ddd, *J* = 12.9, 10.3, 5.8 Hz, 1H, H_{PC}). **^{13}C NMR (100 MHz, $CDCl_3$, ppm):** δ = 153.3 (C_q, C_{Ar}), 150.7 (C_q, C_{Ar}), 141.3 (C_q, C_{Ar}), 141.1 (C_q, C_{Ar}), 140.2 (C_q, C_{Ar}), 139.2 (C_q, C_{Ar}), 135.7 (+, CH, C_{Ar}), 135.2 (+, CH, C_{Ar}), 133.3 (+, CH, C_{Ar}), 133.0 (+, CH, C_{Ar}), 132.0 (+, CH, C_{Ar}), 131.3 (+, CH, C_{Ar}), 130.6 (+, CH, C_{Ar}), 129.2 (+, 2C, CH, C_{Ar}), 122.8 (+, 2C, CH, C_{Ar}), 121.5 (+, CH, C_{Ar}), 35.9 (–, CH_2), 35.3 (–, CH_2), 34.9 (–, CH_2), 32.6 (–, CH_2). **MS (EI, 70 eV, 20 °C, %):** m/z = 313/314 (100/22) $[M+H]^+$. **HRMS-EI (m/z):** $[M+H]^+$, calc. for $C_{22}H_{20}N_2$, 313.1700; found: 313.1696. **IR (ATR, ṽ):** = 2928 (m), 2918 (w), 2850 (w), 1442 (w), 905 (m), 897 (w), 861 (m), 796 (m), 762 (vs), 720 (vs), 686 (vs), 663 (m), 646 (vs), 594 (s), 540 (w), 527 (m), 507 (vs) cm^{-1}.

https://dx.doi.org/10.14272/reaction/SA-FUHFF-UHFFFADPSC-GTJYEJNVJG-UHFFFADPSC-NUHFF-NWIMK-NUHFF-ZZZ

4-(*E*)-Azophenyl-16-bromo[2.2]paracyclophane (27):

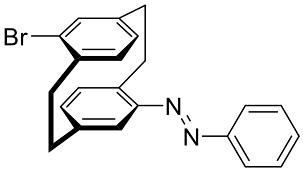

To a solution of 4-amino-16-bromo[2,2]paracyclophane (405 mg, 1.34 mmol, 1 equiv.) in acetic acid (20 mL) nitrosobenzene (287 mg, 2.68 mmol, 2 equiv.) was added and the reaction mixture was stirred for 48 hours at room temperature. The reaction mixture was extracted with dichloromethane, washed with water and brine, dried over $MgSO_4$, filtered, and concentrated under reduced pressure. The crude product was purified via flash column chromatography on silica gel using cyclohexane/ethyl acetate 20:1, obtaining the title compound (263 mg, 0.67 mmol, 50%) as a yellow solid. $\mathbf{R_f}$ = 0.4 (cyclohexane/ethyl acetate 20:1). **^{1}H NMR (400 MHz, $CDCl_3$, ppm):** δ = 8.00–7.92 (m, 2H, H_{Ar}), 7.60–7.53 (m, 2H, H_{Ar}), 7.53–7.47 (m, 1H, H_{Ar}), 7.30 (dd, *J* = 7.9, 2.0 Hz, 1H, H_{Ar}), 6.67 (d, *J* = 2.0 Hz, 1H, H_{Ar}), 6.64–6.58 (m, 2H, H_{Ar}), 6.39 (dd, *J* = 7.8, 1.8 Hz, 1H, H_{Ar}), 6.29 (d, *J* = 7.8 Hz, 1H, H_{Ar}), 4.26 (ddd, *J* = 13.1, 7.6, 5.8 Hz, 1H, H_{PC}), 3.52 (ddd, *J* = 13.1, 10.2, 2.6 Hz, 1H, H_{PC}), 3.26–3.05 (m, 4H, H_{PC}), 3.02–2.93 (m, 1H, H_{PC}), 2.86 (ddd, *J* = 13.3, 10.7, 5.5 Hz, 1H, H_{PC}). **^{13}C NMR (100 MHz, $CDCl_3$, ppm):** δ = 153.2 (C_q, C_{Ar}), 150.9 (C_q, C_{Ar}), 142.1 (C_q, C_{Ar}), 140.7 (C_q, C_{Ar}), 140.6 (C_q, C_{Ar}), 138.7 (C_q, C_{Ar}), 137.4 (+, CH, C_{Ar}), 134.2 (+, CH, C_{Ar}), 133.8 (+, CH, C_{Ar}), 132.2 (+, CH, C_{Ar}), 130.7 (+, CH, C_{Ar}), 130.2 (+, CH, C_{Ar}), 129.2 (+, 2C, CH, C_{Ar}), 126.8 (C_q, C_{Ar}), 122.8 (+, 2C, CH, C_{Ar}), 121.7 (+, CH, C_{Ar}), 35.3 (–, CH_2), 35.0 (–, CH_2), 32.9 (–, CH_2), 31.9 (–, CH_2). **MS (EI, 70 eV, 20 °C, %):** m/z = 390/392 (8/8) $[M]^+$, 364/366/368 (17/33/16) $[C_{16}H_{14}{}^{79}Br_2]^+$, 311/312 (46/12) $[M–Br]^+$, 207/208/209 (100/58/8) $[M–C_8H_7Br]^+$, 182/184 (77/72) $[C_8H_7Br]^+$. **HRMS-EI (m/z):** $[M]^+$, calc. for $C_{22}H_{19}{}^{79}BrN_2$, 390.0732; found: 390.2492. **IR (ATR, ṽ):** = 1050 (vs), 1034 (vs), 965 (m), 798 (m), 792 (m), 766 (m), 449 (vs), 442 (vs), 433 (vs), 418 (vs), 399 (vs), 390 (vs), 378 (vs) cm^{-1}.
https://dx.doi.org/10.14272/reaction/SA-FUHFF-UHFFFADPSC-QSBJTYQFFM-UHFFFADPSC-NUHFF-NWMXW-NUHFF-ZZZ

4-((*E*)-Azo-2',6'-difluorophenyl)-16-bromo[2.2]paracyclophane (28):

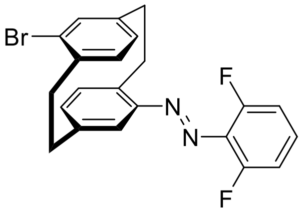

To a solution of 4-amino-16-bromo[2,2]paracyclophane (268 mg, 0.89 mmol, 1 equiv.) in acetic acid (10 mL) nitrosobenzene (254 mg, 1.77 mmol, 2 equiv.) was added and the reaction mixture was stirred for 48 hours at room temperature. The reaction mixture was extracted with dichloromethane, washed with water and brine, dried over $MgSO_4$, filtered, and concentrated under reduced pressure. The crude product was purified via flash column chromatography on silica gel using cyclohexane/ethyl acetate 20:1, obtaining the title compound (166 mg, 0.39 mmol, 44%) as a orange solid. $\mathbf{R_f}$ = 0.42 (cyclohexane/ethyl acetate 20:1). **^{1}H NMR (400 MHz, $CDCl_3$, ppm)**: δ = 7.30–7.22 (m, 2H, H_{Ar}), 7.06–6.97 (m, 2H, , H_{Ar}), 6.59–6.54 (m, 3H, H_{Ar}), 6.45 (dd, *J* = 7.8, 1.8 Hz, 1H, H_{Ar}), 6.25 (d, *J* = 7.8 Hz, 1H, H_{Ar}), 4.10 (ddd, *J* = 13.1, 9.9, 3.5 Hz, 1H, H_{pc}), 3.45 (ddd, *J* = 13.0, 10.1, 2.5 Hz, 1H, H_{pc}), 3.19–2.95 (m, 4H, H_{pc}), 2.93–2.73 (m, 2H, H_{pc}). **^{13}C NMR (100 MHz, $CDCl_3$, ppm):** δ = 152.4 (C_q, C-F), 151.9 (C_q, C-F), 145.0 (C_q, C_{Ar}), 143.4 (C_q, C_{Ar}), 142.3 (C_q, C_{Ar}), 141.6 (C_q, C_{Ar}), 140.8 (C_q, C_{Ar}), 138.7 (C_q, C_{Ar}), 137.5 (+, CH, C_{Ar}), 134.3 (+, CH, C_{Ar}), 133.9 (+, CH, C_{Ar}), 133.1 (+, CH, C_{Ar}), 130.1 (+, CH, C_{Ar}), 129.7 (+, t, *J* = 10.0 Hz, CH, C_{Ar}), 126.8 (C_q, C_{Ar}), 121.0 (+, CH, C_{Ar}), 112.7–112.4 (+, m, 2C, CH, C_{Ar}), 35.3 (–, CH_2), 34.7 (–, CH_2), 32.8 (–, CH_2), 31.8 (–, CH_2).

MS (ESI, 70 eV, 20 °C, %): m/z = 427/429 (21/20) $[M+H]^+$. **HRMS-ESI (m/z):** $[M+H]^+$, calc. for $C_{22}H_{18}{}^{79}BrF_2N_2$, 427.0616; found: 427.0615. **IR (ATR, ṽ):** = 2927 (m), 1615 (w), 1587 (m), 1475 (vs), 1442 (m), 1435 (m), 1239 (s), 1024 (vs), 977 (w), 898 (m), 880 (w), 836 (m), 775 (vs), 734 (m), 710 (m), 669 (m), 654 (m), 514 (s), 487 (m), 475 (s) cm^{-1}.
https://dx.doi.org/10.14272/reaction/SA-FUHFF-UHFFFADPSC-OTKUMVPKUY-UHFFFADPSC-NUHFF-NYMLC-NUHFF-ZZZ

4-(4'-Pyridinyl)-16-(*E*)-azophenyl[2.2]paracyclophane (29):

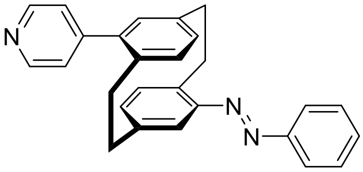

A vessel was charged with 4-azophenyl-16-bromo[2,2]paracyclophane (245 mg, 0.67 mmol, 1 equiv.), pyridine-4-boronic acid (128 mg, 1.0 mmol, 1.7 equiv.), palladium-tetrakis(triphenylphosphine) (46 mg, 0.04 mmol, 0.06 equiv.), potassium phosphate (341 mg, 1.61 mmol, 2.5 equiv.), dioxane (8 mL) and water (2 mL). The mixture was heated under reflux for 16 h. Then the reaction mixture was then cooled to room temperature, extracted with ether (3 × 30 mL), and the extracts were dried ($MgSO_4$) and evaporated under reduced pressure. The crude product was purified via flash column chromatography on silica gel using dichloromethane /ethyl acetate 4:1, obtaining the title compound (98 mg, 0.25 mmol, 40%) as an orange solid. $\mathbf{R_f}$ = 0.5 (dichloromethane /ethyl acetate 1:1). **^{1}H NMR (400 MHz, $CDCl_3$, ppm)**: δ = 8.73 (d, *J* = 5.1 Hz, 2H, H_{Py}), 8.03–7.95 (m, 2H, H_{Ar}), 7.58 (dd, J = 8.3, 6.6 Hz, 2H, H_{Ar}), 7.54–7.49 (m, 1H, H_{Ar}), 7.45–7.41 (m, 2H, H_{Py}), 6.81 (d, *J* = 2.0 Hz, 1H, H_{Ar}), 6.73–6.63 (m, 3H, H_{Ar}), 6.53–6.45 (m, 2H, H_{Ar}), 4.37–4.26 (m, 1H, H_{pc}), 3.40 (ddd, *J* = 13.7, 10.1, 3.9 Hz, 1H, H_{pc}), 3.25 (dd, *J* = 9.1, 5.4 Hz, 2H, H_{pc}), 3.10–2.98 (m, 2H, H_{pc}), 2.92 (ddd, *J* = 14.1, 10.4, 3.9 Hz, 1H, H_{pc}), 2.72 (ddd, *J* = 13.8, 10.1, 4.3 Hz, 1H, H_{pc}). **^{13}C NMR (100 MHz, $CDCl_3$, ppm)**: δ = 153.2 (C_q, C_{Ar}), 151.0 (C_q, C_{Ar}), 150.1 (+, CH, 2C, C_{Py}), 148.6 (C_q, C_{Py}), 141.1 (+, CH, C_{Ar}), 141.0 (+, CH, C_{Ar}), 140.7 (+, CH, C_{Ar}), 139.6 (+, CH, C_{Ar}), 136.9 (+, CH, C_{Ar}), 135.1 (+, CH, C_{Ar}), 134.0 (+, CH, C_{Ar}), 132.9 (+, CH, C_{Ar}), 132.1 (+, CH, C_{Ar}), 131.9 (+, CH, C_{Ar}), 130.8 (+, CH, C_{Ar}), 129.2 (+, CH, 2C, C_{Ar}), 124.6 (+, CH, 2C, C_{Ar}), 122.9 (+, CH, 2C, C_{Py}), 121.8 (+, CH, C_{Ar}), 35.5 (–, CH_2), 34.4 (–, CH_2), 33.4 (–, CH_2), 32.1 (–, CH_2). **MS (ESI, 70 eV, 20 °C, %)**: m/z = 389/390 (81/23) $[M]^+$, 285 (28) $[M–C_6H_5N_2]^+$, 311/312 (46/12) $[M–Br]^+$, 207/208 (100/63) $[M–C_{13}H_{11}N]^+$, 181/182 (24/56) $[C_{13}H_{12}N]^+$. **HRMS-ESI (m/z):** $[M]^+$, calc. for $C_{27}H_{23}N_3$, 389.1892; found: 389.3588. **IR (ATR, ṽ)**: = 1047 (vs), 973 (m), 795 (m), 441 (vs), 432 (vs), 391 (vs), 380 (vs) cm^{-1}.
https://dx.doi.org/10.14272/reaction/SA-FUHFF-UHFFFADPSC-WEVXSCCUJI-UHFFFADPSC-NUHFF-NGFDO-NUHFF-ZZZ

4-(4'-Pyridinyl)-16-((*E*)-azo-2',6'-difluorophenyl)[2.2]paracyclophane (30):

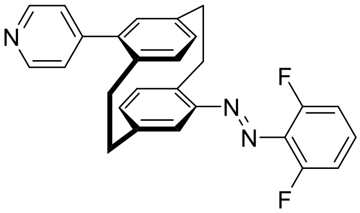

A vessel was charged with 4-((*E*)-Azo-2',6'-difluorophenyl)-16-bromo[2.2]paracyclophane (160 mg, 0.37 mmol, 1 equiv.), pyridine-4-boronic acid (71 mg, 0.58 mmol, 1.5 equiv.), palladium-tetrakis(triphenylphosphine) (23 mg, 0.02 mmol, 0.05 equiv.), potassium phosphate (188 mg, 0.89 mmol, 2.5 equiv.), dioxane (6 mL) and water (3 mL). The mixture was heated under reflux for 16 h. Then the reaction mixture was then cooled to room temperature, extracted with ether (3 × 20 mL), and the extracts were dried ($MgSO_4$) and evaporated under reduced pressure. The crude product was purified via flash column chromatography on silica gel using dichloromethane

/ethyl acetate 4:1, obtaining the title compound (49 mg, 0.12 mmol, 31%) as an orange solid. **R_f** = 0.48 (dichloromethane /ethyl acetate 1:1). **^{1}H NMR (400 MHz, $CDCl_3$, ppm):** δ = 8.68–8.60 (m, 2H, H_{Py}), 7.38–7.34 (m, 2H, H_{Py}), 7.33–7.23 (m, 1H, H_{Ar}), 7.09–6.98 (m, 2H, H_{Ar}), 6.71 (d, J = 1.7 Hz, 1H, H_{Ar}), 6.67–6.61 (m, 3H, H_{Ar}), 6.53 (dd, J = 7.8, 1.9 Hz, 1H, H_{Ar}), 6.45 (d, J = 7.8 Hz, 1H, H_{Ar}), 4.17 (ddd, J = 13.0, 9.5, 3.7 Hz, 1H, H_{PC}), 3.34 (ddd, J = 13.7, 10.1, 3.9 Hz, 1H, H_{PC}), 3.18–3.08 (m, 2H, H_{PC}), 3.01–2.90 (m, 2H, H_{PC}), 2.85 (ddd, J = 14.1, 10.4, 3.9 Hz, 1H, H_{PC}), 2.64 (ddd, J = 13.8, 10.1, 4.3 Hz, 1H, H_{PC}). **^{13}C NMR (100 MHz, $CDCl_3$, ppm):** δ = 157.2 (C_q, d, $J_{C\text{-}F}$ = 4.4 Hz, C-F), 154.6 (C_q, d, $J_{C\text{-}F}$ = 4.4 Hz, C-F), 151.9 (C_q, C_{Ar}), 150.2 (+, 2C, C_{Py}), 148.6 (C_q, C_{Py}), 142.0 (C_q, C_{Ar}), 141.2 (C_q, C_{Ar}), 140.9 (C_q, C_{Ar}), 139.5 (C_q, C_{Ar}), 136.8 (C_q, C_{Ar}), 135.4 (C_q, C_{Ar}), 135.2 (+, CH, C_{Ar}), 134.1 (+, CH, C_{Ar}), 133.9 (+, CH, C_{Ar}), 132.2 (+, CH, C_{Ar}), 131.8 (+, CH, C_{Ar}), 129.8 (+, t, J = 10.0 Hz, CH, C_{Ar}), 124.5 (+, 2C, C_{Py}), 121.1 (+, CH, C_{Ar}), 112.7–112.4 (+, m, 2C, CH, C_{Ar}), 35.2 (–, CH_2), 34.4 (–, CH_2), 33.4 (–, CH_2), 32.0 (–, CH_2). **MS (ESI, 70 eV, 20 °C, %):** m/z = 426/427(72/20) $[M+H]^+$. **HRMS-ESI (m/z):** $[M+H]^+$, calc. for $C_{27}H_{22}F_2N_3$, 426.1777; found: 426.1775. **IR (ATR, ṽ)** = 3053 (vw), 3024 (vw), 2925 (w), 2850 (w), 1613 (w), 1591 (s), 1540 (w), 1475 (m), 1451 (w), 1436 (m), 1414 (w), 1400 (w), 1307 (vw), 1278 (w), 1238 (w), 1217 (w), 1191 (s), 1159 (w), 1137 (w), 1118 (s), 1095 (w), 1068 (w), 1023 (s), 1011 (m), 992 (m), 979 (w), 945 (w), 912 (w), 899 (w), 878 (w), 858 (w), 844 (w), 829 (m), 781 (s), 748 (m), 735 (w), 718 (vs), 694 (vs), 669 (w), 653 (m), 620 (m), 577 (w), 538 (vs), 506 (s), 482 (s), 458 (m), 387 (w) cm^{-1}.
https://dx.doi.org/10.14272/reaction/SA-FUHFF-UHFFFADPSC-BRNWOJQAZQ-UHFFFADPSC-NUHFF-NLWWK-NUHFF-ZZZ

4-(*N*-Methyl-4'-pyridinium)-16-(*E*)-Azophenyl- [2.2]paracyclophane iodide (31):

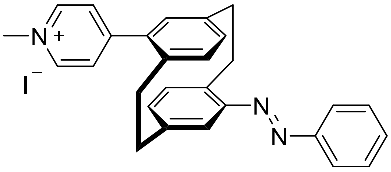

4-(4'-Pyridinyl)-16-(*E*)-azophenyl[2,2]paracyclophane (199 mg, 1 equiv.) dissolved in acetonitrile 6 mL, then methyl iodide (0.3 mL) was added. The mixture was stirred at 40 °C for 16 h excluded of the light. The solvent was removed under reduced pressure to give a residue and then recrystallized from methanol to obtain 4-(*N*-methyl-4'-pyridinium)-16-(*E*)-azophenyl[2.2]paracyclophane iodide (132 mg, 0.33 mmol, 80%) as an orange solid. **^{1}H NMR (400 MHz, DMSO-d6, ppm):** δ = 9.04 (d, J = 6.4 Hz, 2H, H_{Py}), 8.32–8.27 (m, 2H, H_{Py}), 8.02–7.94 (m, 2H, H_{Ar}), 7.71–7.55 (m, 3H, H_{Ar}), 7.08 (d, J = 1.8 Hz, 1H, H_{Ar}), 7.03 (d, J = 7.9 Hz, 1H, H_{Ar}), 6.75 (d, J = 2.0 Hz, 1H, H_{Ar}), 6.65 (d, J = 7.7 Hz, 1H, H_{Ar}), 6.58 (dd, J = 7.9, 1.9 Hz, 1H, H_{Ar}), 6.50 (dd, J = 7.8, 1.8 Hz, 1H, H_{Ar}), 4.41 (s, 3H, CH_3), 4.23–4.13 (m, 1H, H_{pc}), 3.33–3.27 (m, 2H, H_{pc}), 3.24–3.08 (m, J = 19.3, 10.4, 5.2 Hz, 3H, H_{pc}), 2.96 (ddd, J = 13.9, 10.2, 4.0 Hz, 1H, H_{pc}), 2.71–2.60 (m, 1H, H_{pc}). **^{13}C NMR (100 MHz, DMSO-d6, ppm):** δ = 155.5 (C_q, C_{Py}), 153.2 (C_q, C_{Ar}), 150.9 (C_q, C_{Ar}), 145.9 (+, CH, 2C, C_{Py}), 144.3 (C_q, C_{Ar}), 141.4 (C_q, C_{Ar}), 141.3 (C_q, C_{Ar}), 138.6 (C_q, C_{Ar}), 137.0 (C_q, C_{Ar}), 136.0 (+, CH, C_{Ar}), 134.8 (+, CH, C_{Ar}), 133.8 (+, CH, C_{Ar}), 133.5 (+, CH, C_{Ar}), 133.1 (+, CH, C_{Ar}), 131.6 (+, CH, C_{Ar}), 130.0 (+, CH, 2C, C_{Ar}), 127.9 (+, CH, 2C, C_{Ar}), 123.0 (+, CH, 2C, C_{Py}), 121.9 (+, CH, C_{Ar}), 47.6 (+, CH_3), 35.2 (–, CH_2), 34.3 (–, CH_2), 33.3 (–, CH_2), 31.7 (–, CH_2). **MS (ESI, 70 eV, 20 °C, %):** m/z = 404/405 (100/30) $[M–I]^+$. **HRMS-ESI (m/z):** $[M–I]^+$, calc. for $C_{27}H_{23}N_3$, 404.2122; found: 404.2118. **IR (ATR, ṽ)** = 3026 (m), 2961 (w), 2942 (m), 2928 (m), 2851 (m), 1635 (vs), 1585 (m), 1561 (m), 1548 (m), 1509 (m), 1479 (m), 1466 (m), 1451 (vs), 1439 (vs), 1402 (m), 1322 (m), 1288 (m), 1198 (vs), 1143 (vs), 1122 (s), 1094 (m), 1069 (s), 1043 (m), 914 (m), 901 (m), 878 (m), 861 (s), 847 (vs), 802 (m), 773 (vs), 739 (s), 727 (s), 690 (vs), 667 (s), 647 (vs), 620 (m), 585

(m), 555 (s), 531 (s), 510 (vs), 484 (vs), 459 (s), 446 (vs), 429 (s), 412 (m), 402 (m), 391 (s), 381 (m) cm^{-1}.
https://dx.doi.org/10.14272/reaction/SA-FUHFF-UHFFFADPSC-BYPDVTHQGX-UHFFFADPSC-NUHFF-MLXNZ-NUHFF-ZZZ

4-(*N*-Methyl-4'-pyridinium)-16-((*E*)-Azo-2',6'-difluorophenyl)[2.2]paracyclophane iodide (32):

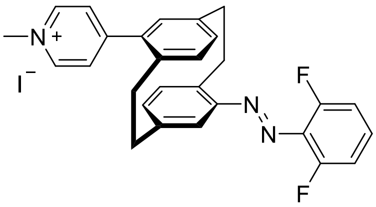

4-(4'-Pyridinyl)-16-((*E*)-azo-2',6'-difluorophenyl)[2.2]paracyclophane (60 mg, 0.14 mmol, 1 equiv.) dissolved in acetonitrile 5 mL, then methyl iodide (0.1 mL) was added. The mixture was stirred at 40 °C for 16 h excluded of the light. The solvent was removed under reduced pressure to give a residue and then recrystallized from methanol to obtain 4-(*N*-methyl-4'-pyridinium)-16-((*E*)-Azo-2',6'-difluorophenyl)[2.2]paracyclophane iodide (38 mg, 0.07 mmol, 47%) as an orange solid. **^{1}H NMR (400 MHz, CDCl$_3$, ppm):** δ = 9.19 (d, *J* = 6.3 Hz, 2H, H_{Py}), 8.05 (d, *J* = 6.9 Hz, 2H, H_{Py}), 7.29 (tt, *J* = 8.4, 5.9 Hz, 1H, H_{Ar}), 7.03 (t, *J* = 8.6 Hz, 2H, H_{Ar}), 6.83–6.73 (m, 2H, H_{Ar}), 6.68–6.58 (m, 3H, H_{Ar}), 6.50 (d, *J* = 7.8 Hz, 1H, H_{Ar}), 4.64 (s, 3H, CH_3), 4.17–4.08 (m, 1H, H_{pc}), 3.29 (ddd, *J* = 13.9, 9.5, 4.3 Hz, 1H, H_{pc}), 3.20–3.11 (m, 2H, H_{pc}), 3.07–2.95 (m, 2H, H_{pc}), 2.90–2.75 (m, 2H, H_{pc}). **^{13}C NMR (100 MHz, CDCl$_3$, ppm):** δ = 157.2 (C_q, d, $J_{C\text{-}F}$ = 5.2 Hz, C-F), 154.6 (C_q, d, $J_{C\text{-}F}$ = 5.2 Hz, C-F), 156.9 (C_q, C_{Py}), 151.9 (C_q, C_{Ar}), 145.3 (+, CH, 2C, C_{Py}), 142.2 (C_q, C_{Ar}), 142.0 (C_q, C_{Ar}), 140.9 (C_q, C_{Ar}), 139.9 (C_q, C_{Ar}), 138.0 (C_q, C_{Ar}), 136.0 (+, CH, 2C, C_{Ar}), 134.3 (+, CH, C_{Ar}), 134.2 (+, CH, C_{Ar}), 134.0 (+, CH, C_{Ar}), 132.8 (+, CH, C_{Ar}), 130.1 (+, t, *J* = 10.0 Hz, CH, C_{Ar}), 128.1 (+, CH, 2C, C_{Py}), 121.4, 112.7–112.5 (+, m, 2C, CH, C_{Ar}), 48.9 (+, CH_3), 35.0 (–, CH_2), 34.8 (–, CH_2), 33.4 (–, CH_2), 31.9 (–, CH_2). **MS (ESI, 70 eV, 20 °C, %)** m/z = 440/441 (100/29) [M–I]$^+$. **HRMS-ESI (m/z):** [M–I]$^+$, calc. for $C_{28}H_{24}F_2N_3$, 440.1933; found: 440.1916. **IR (ATR, ṽ)** = 2924 (s), 1639 (vs), 1613 (vs), 1587 (s), 1567 (m), 1548 (m), 1516 (s), 1477 (vs), 1465 (vs), 1449 (s), 1239 (s), 1222 (m), 1194 (s), 1026 (vs), 1014 (vs), 843 (s), 783 (vs), 720 (s), 656 (m), 507 (m), 487 (vs) cm^{-1}.
https://dx.doi.org/10.14272/reaction/SA-FUHFF-UHFFFADPSC-JDTNNFQGVP-UHFFFADPSC-NUHFF-MBMRQ-NUHFF-ZZZ

3 Copies of NMR Spectra

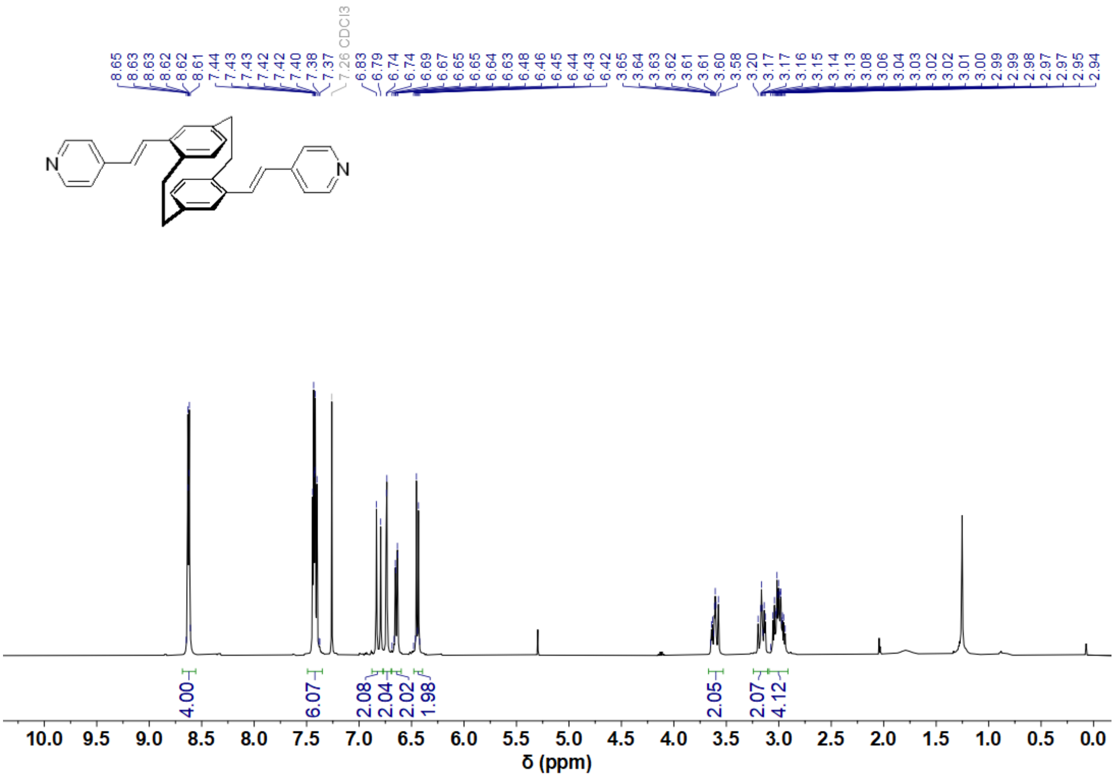

Figure S 1 ^{1}H NMR spectra (400 MHz, $CDCl_3$, r.t.) for **4,16-di(4'-pyridyl-(*E*)-vinyl[2.2]paracyclophane**

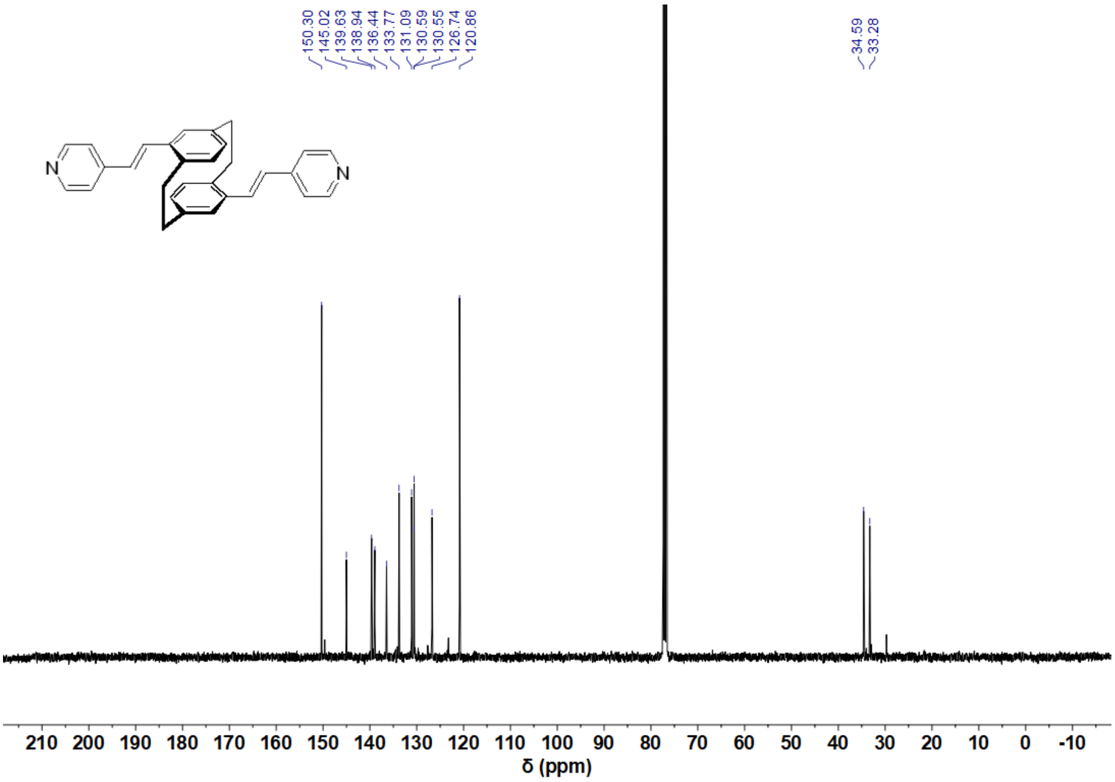

Figure S 2 ^{13}C NMR spectra (400 MHz, $CDCl_3$, r.t.) for **4,16-di(4'-pyridyl-(*E*)-vinyl[2.2]paracyclophane**

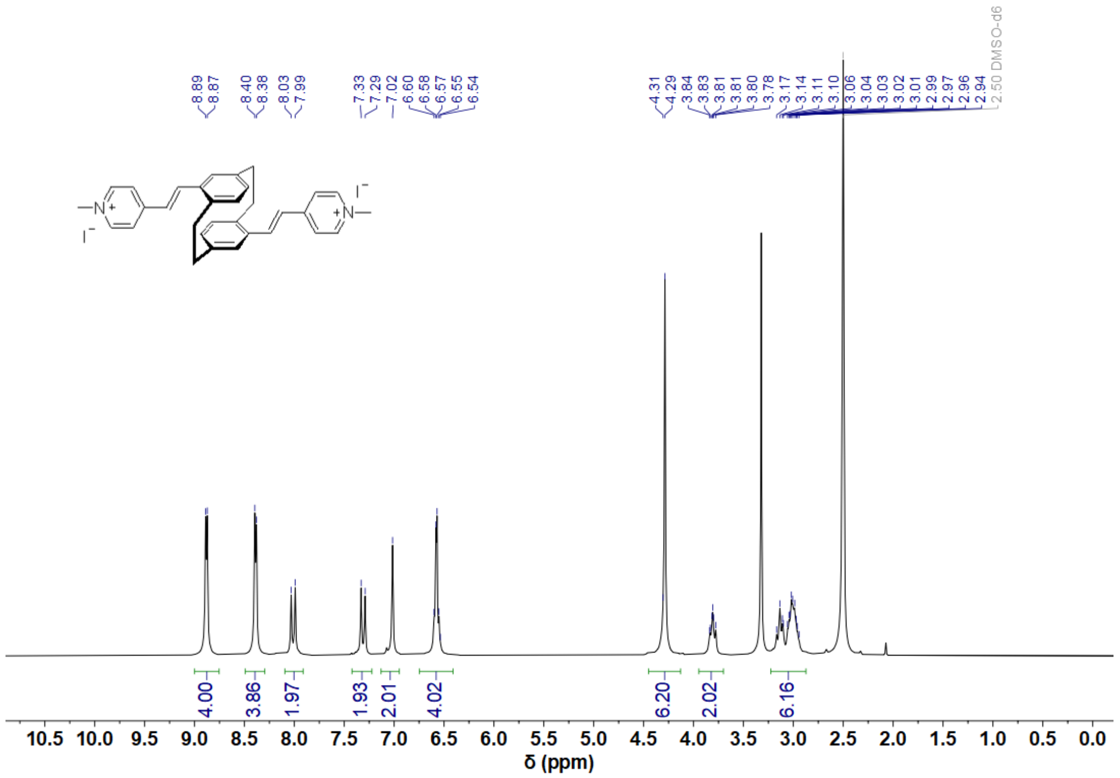

Figure S 3 ^{1}H NMR spectra (400 MHz, $CDCl_3$, r.t.) for **4,16-di(*N*-methyl-4'-pyridinium-(*E*)-vinyl)[2.2]paracyclophane iodide (pDMPCP)**

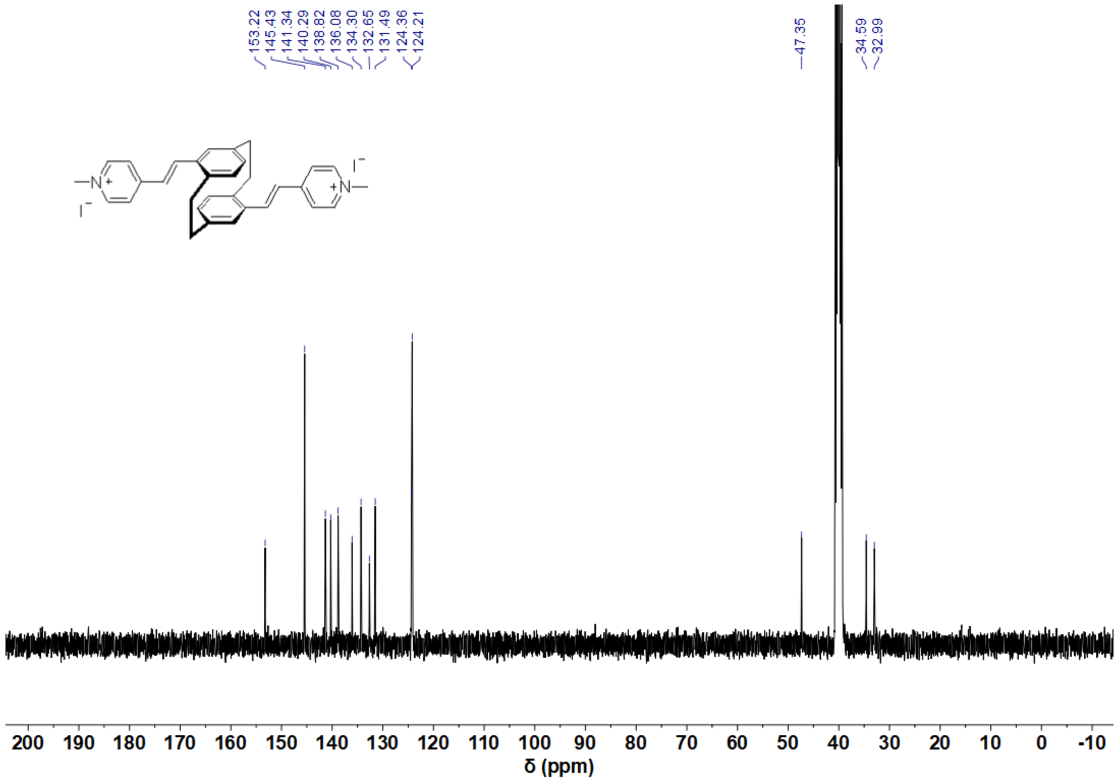

Figure S 4 ^{13}C NMR spectra (400 MHz, $CDCl_3$, r.t.) for **4,16-di(*N*-methyl-4'-pyridinium-(*E*)-vinyl)[2.2]paracyclophane iodide (pDMPCP)**

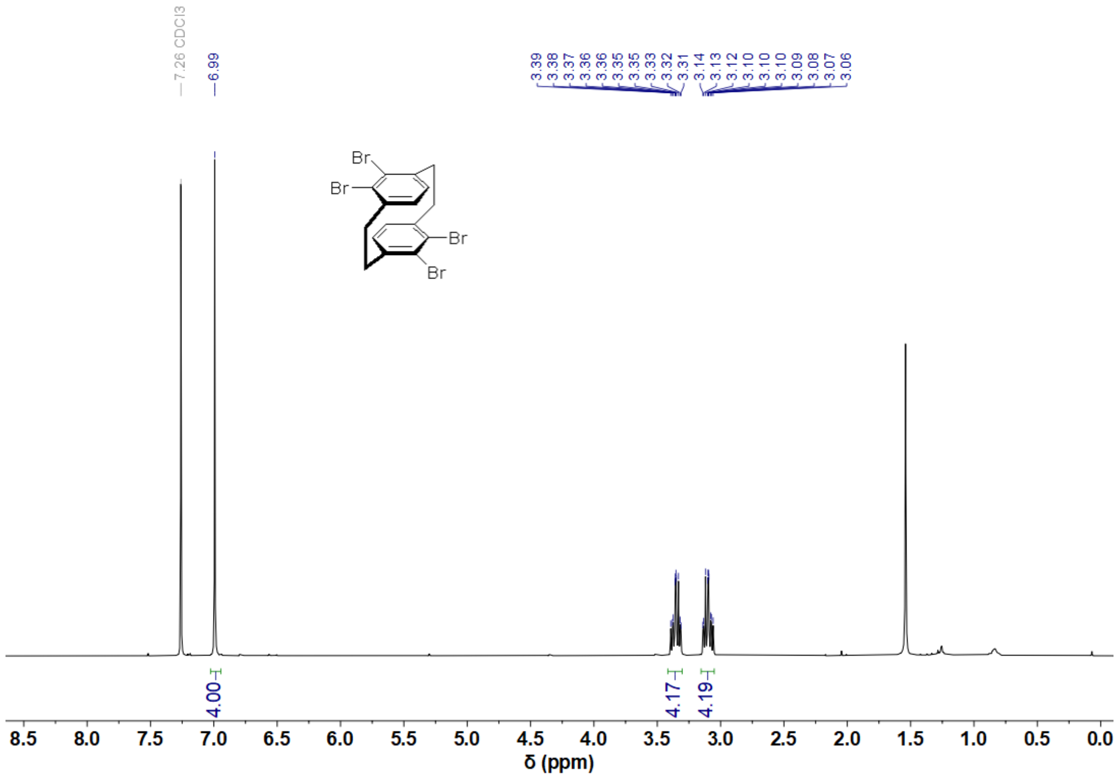

Figure S 5 ^{1}H NMR spectra (400 MHz, $CDCl_3$, r.t.) for **4,5,12,13-tetrabromo[2.2]paracyclophane**

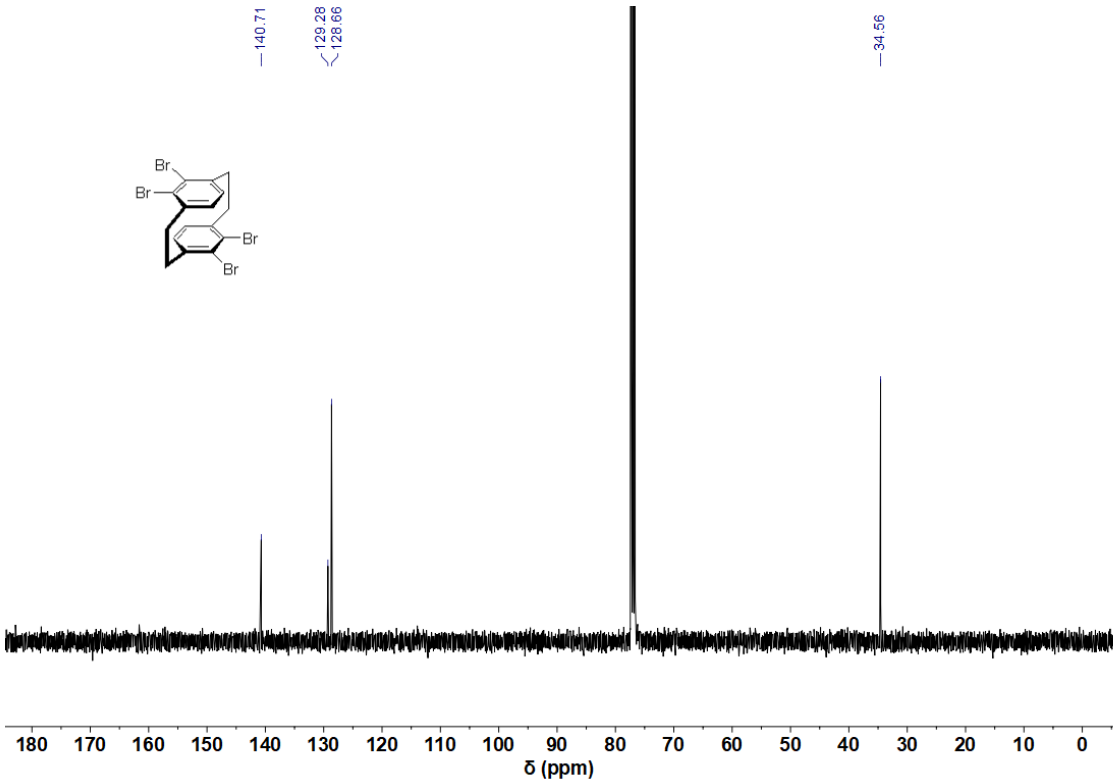

Figure S 6 ^{13}C NMR spectra (400 MHz, $CDCl_3$, r.t.) for **4,5,12,13-tetrabromo[2.2]paracyclophane**

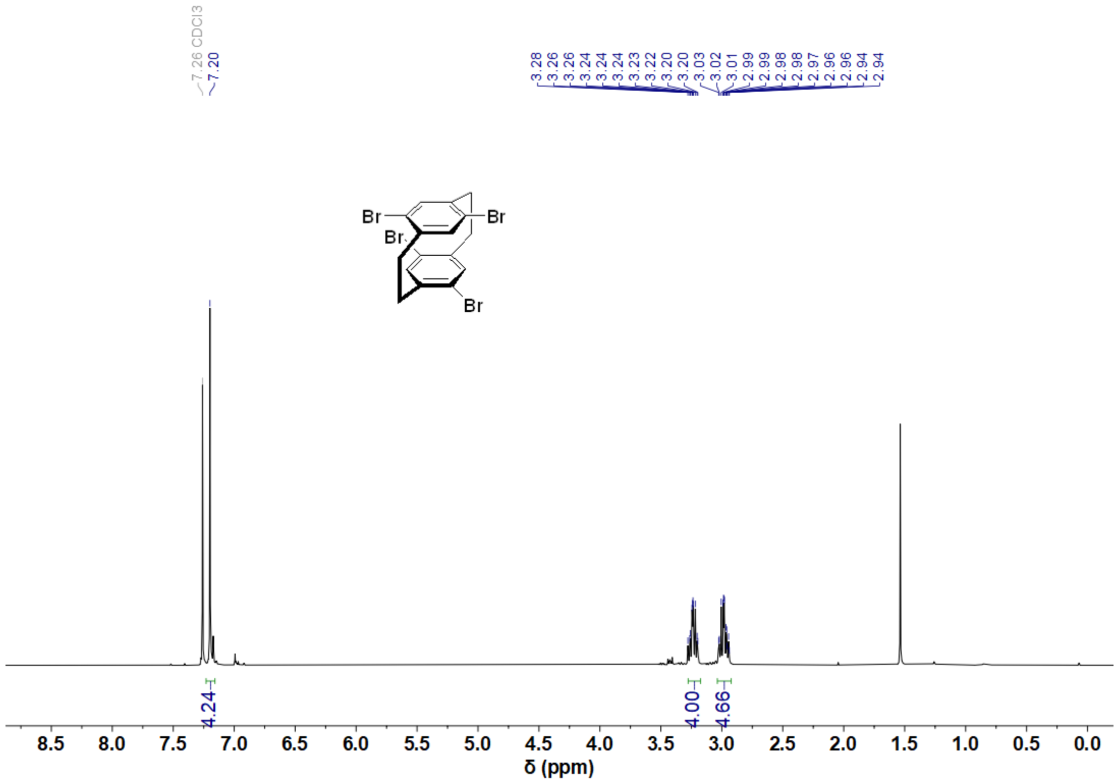

Figure S 7 ^{1}H NMR spectra (400 MHz, $CDCl_3$, r.t.) for **4,7,12,15-tetrabromo[2.2]paracyclophane**

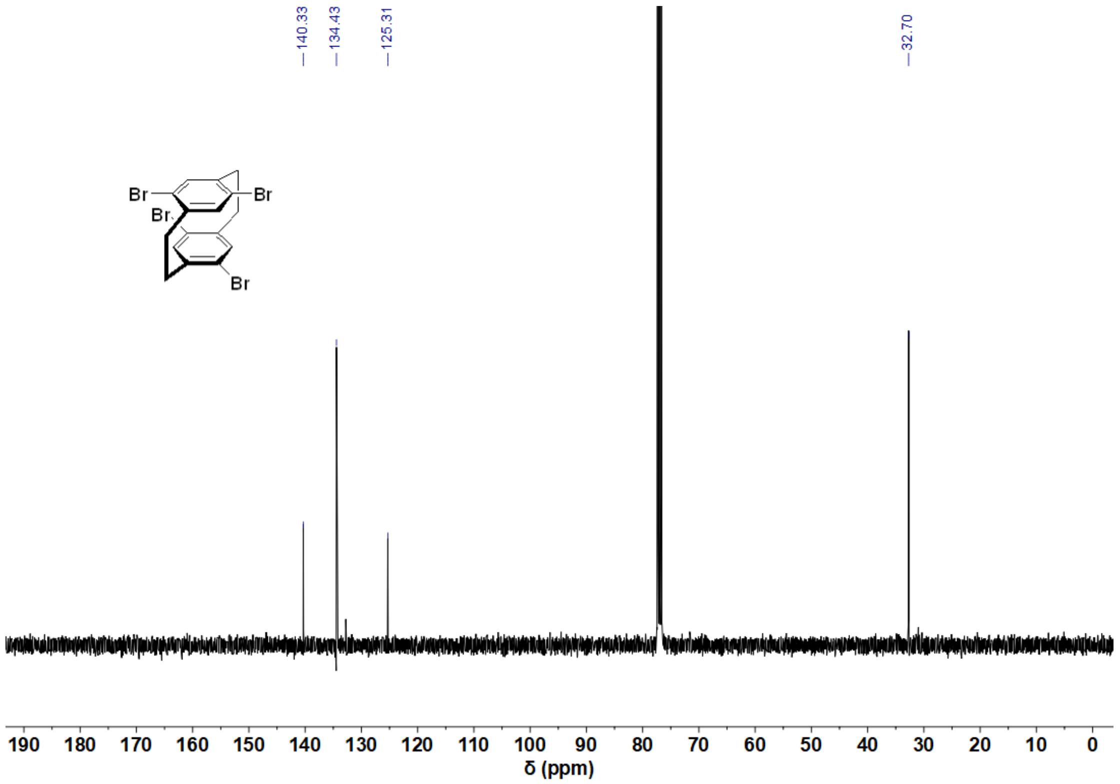

Figure S 8 ^{13}C NMR spectra (400 MHz, $CDCl_3$, r.t.) for **4,7,12,15-tetrabromo[2.2]paracyclophane**

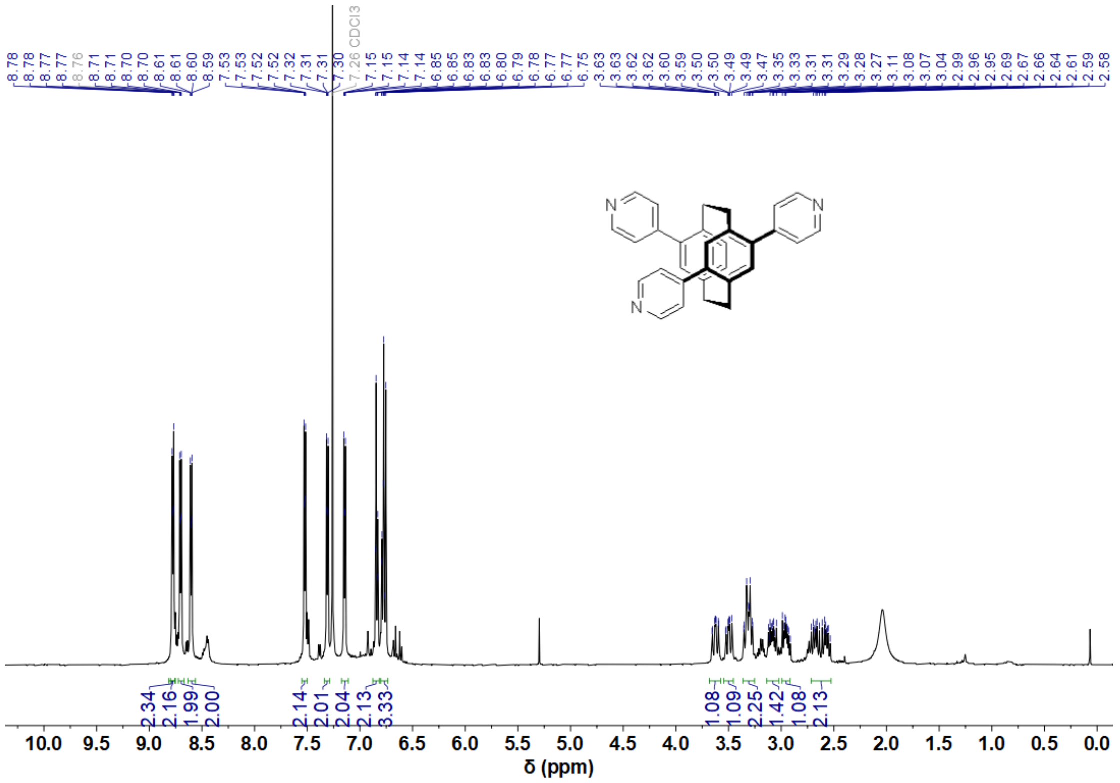

Figure S 9 ¹H NMR spectra (400 MHz, $CDCl_3$, r.t.) for **4,7,12-tri(4'-pyridinyl)[2.2]paracyclophane**

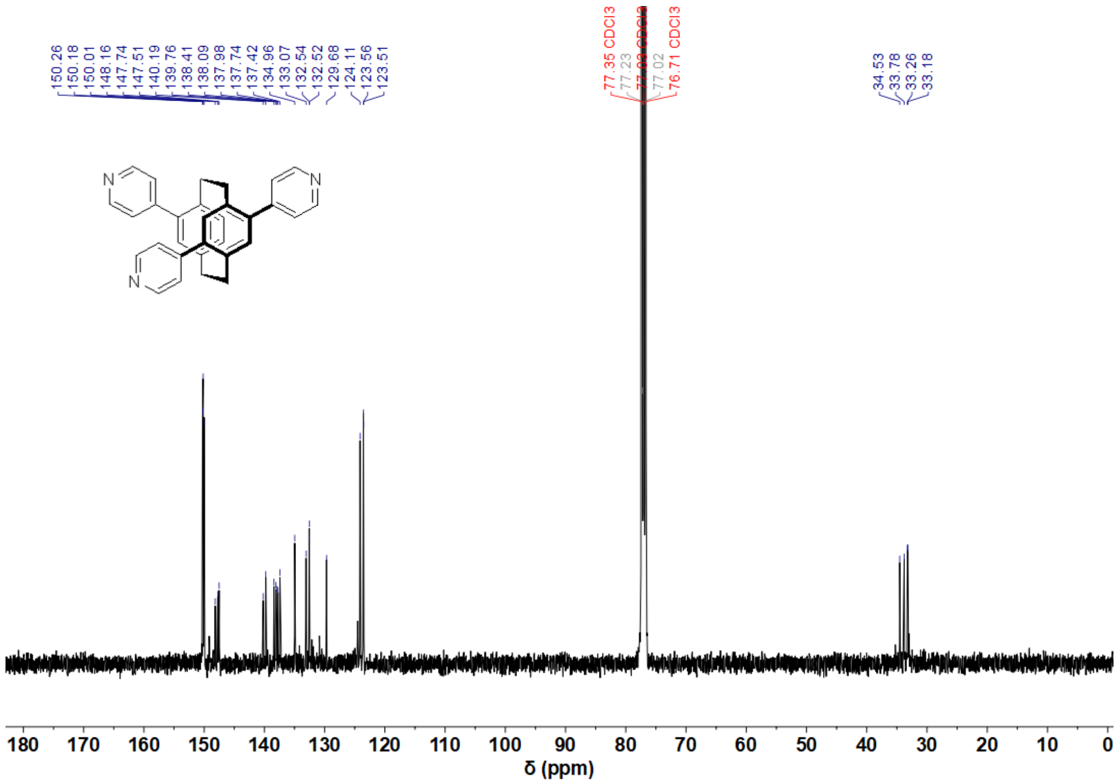

Figure S 10 ¹³C NMR spectra (400 MHz, $CDCl_3$, r.t.) for **4,7,12-tri(4'-pyridinyl)[2.2]paracyclophane**

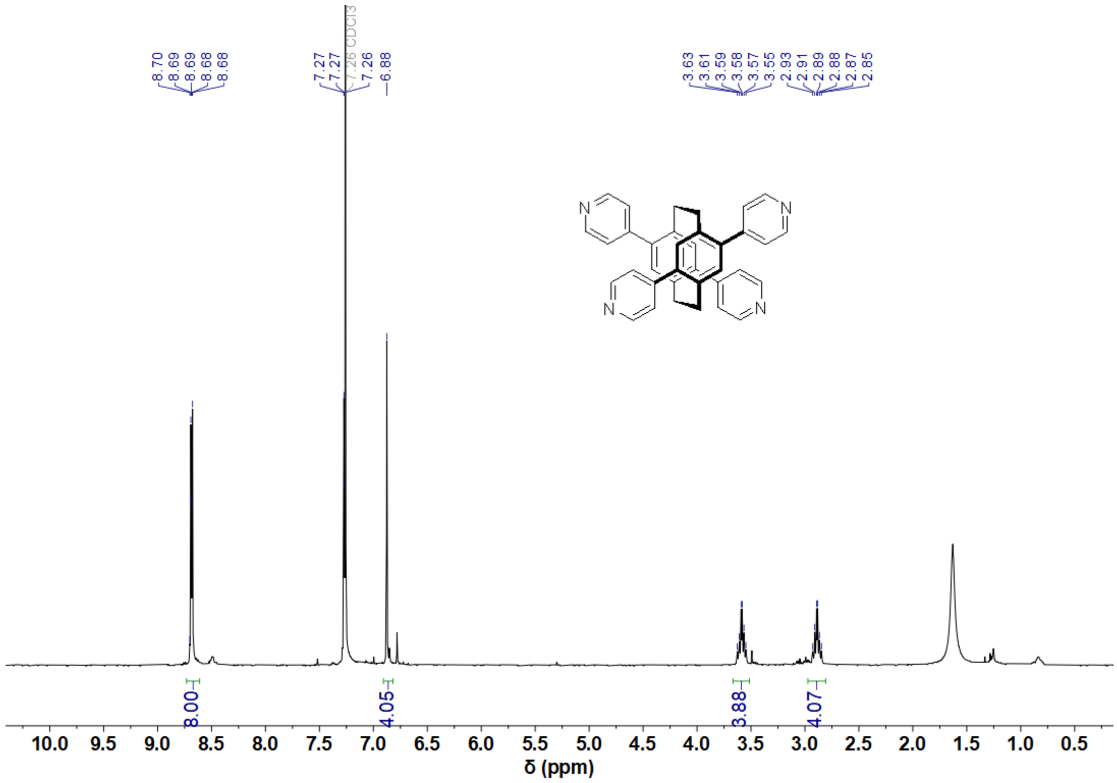

Figure S 11 ^{1}H NMR spectra (400 MHz, $CDCl_3$, r.t.) for **4,7,12,15-tetra(4'-pyridinyl)[2.2]paracyclophane**

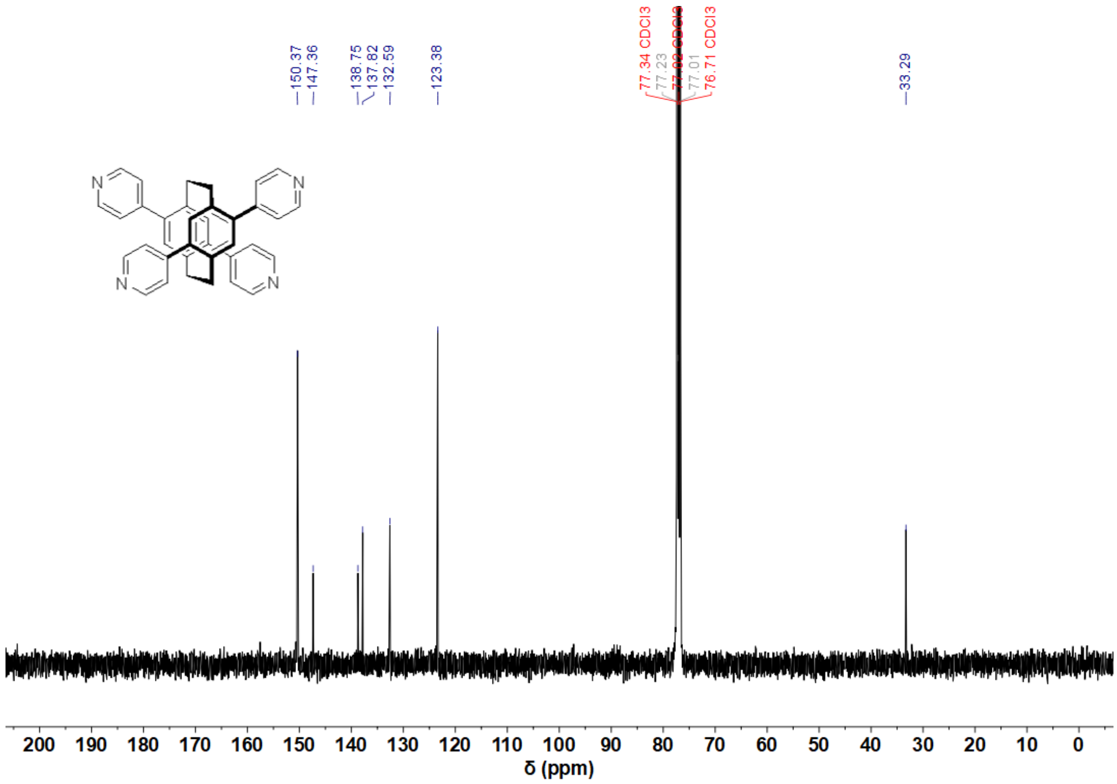

Figure S 12 ^{13}C NMR spectra (400 MHz, $CDCl_3$, r.t.) for **4,7,12,15-tetra(4'-pyridinyl)[2.2]paracyclophane**

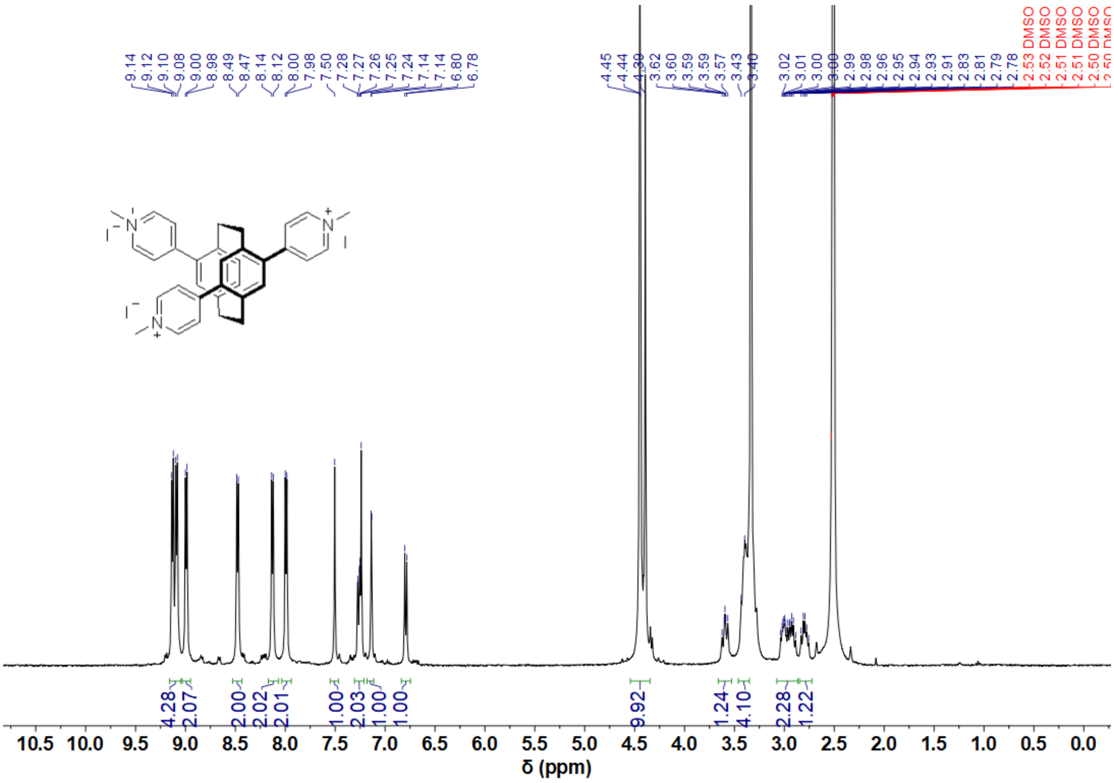

Figure S 13 ^{1}H NMR spectra (400 MHz, $CDCl_3$, r.t.) for **4,7,12-tri(*N*-methyl-4'-pyridinium)[2.2]paracyclophane iodide (TMPCP)**

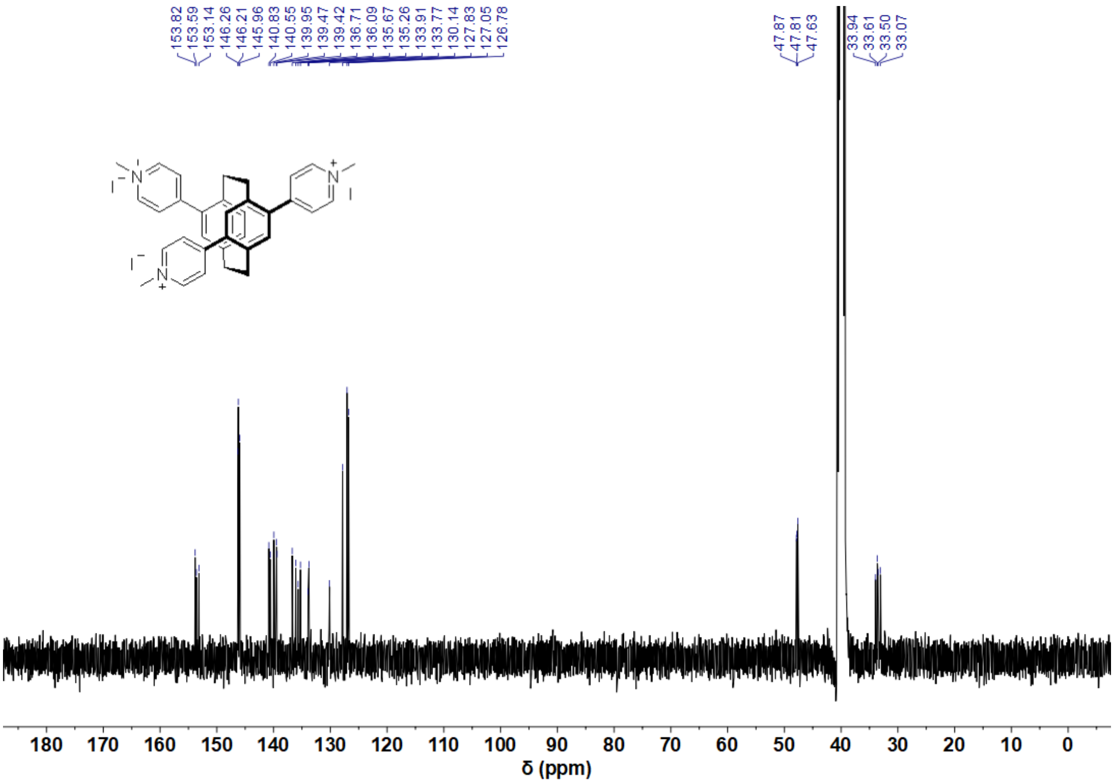

Figure S 14 ^{13}C NMR spectra (400 MHz, $CDCl_3$, r.t.) for **4,7,12-tri(*N*-methyl-4'-pyridinium)[2.2]paracyclophane iodide (TMPCP)**

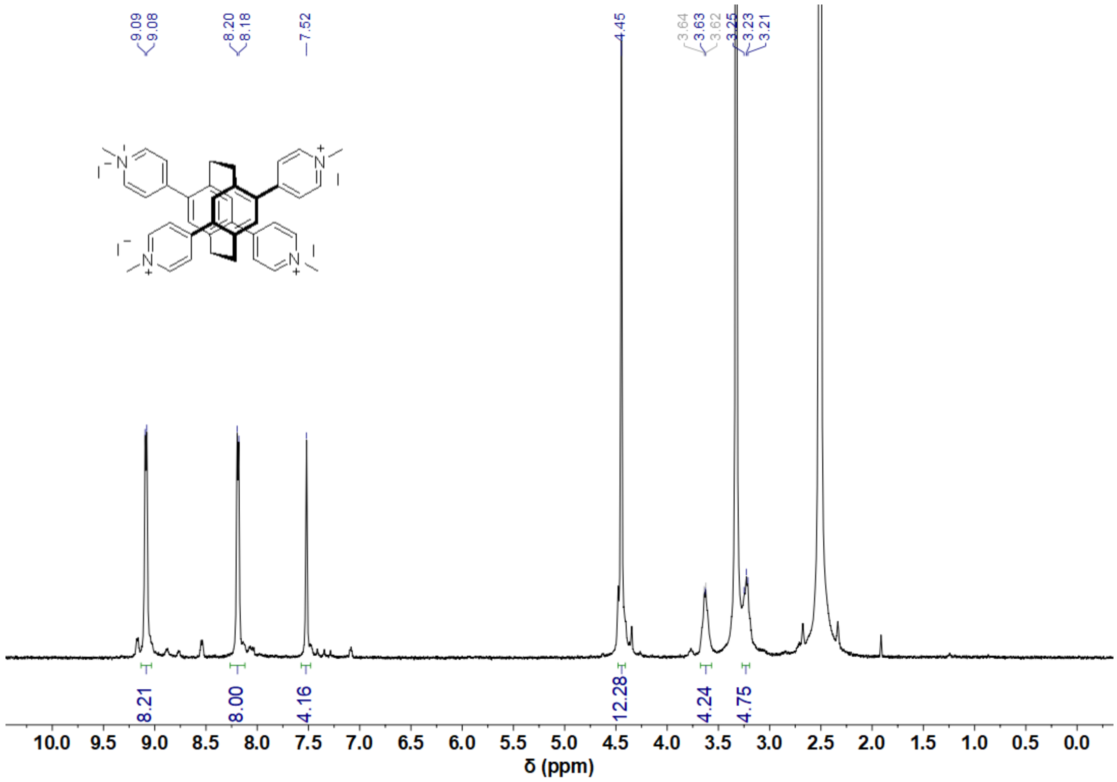

Figure S 15 ^{1}H NMR spectra (400 MHz, $CDCl_3$, r.t.) for **4,7,12,15-tetra(*N*-methyl-4'-pyridinium)[2.2]paracyclophane iodide (QMPCP)**

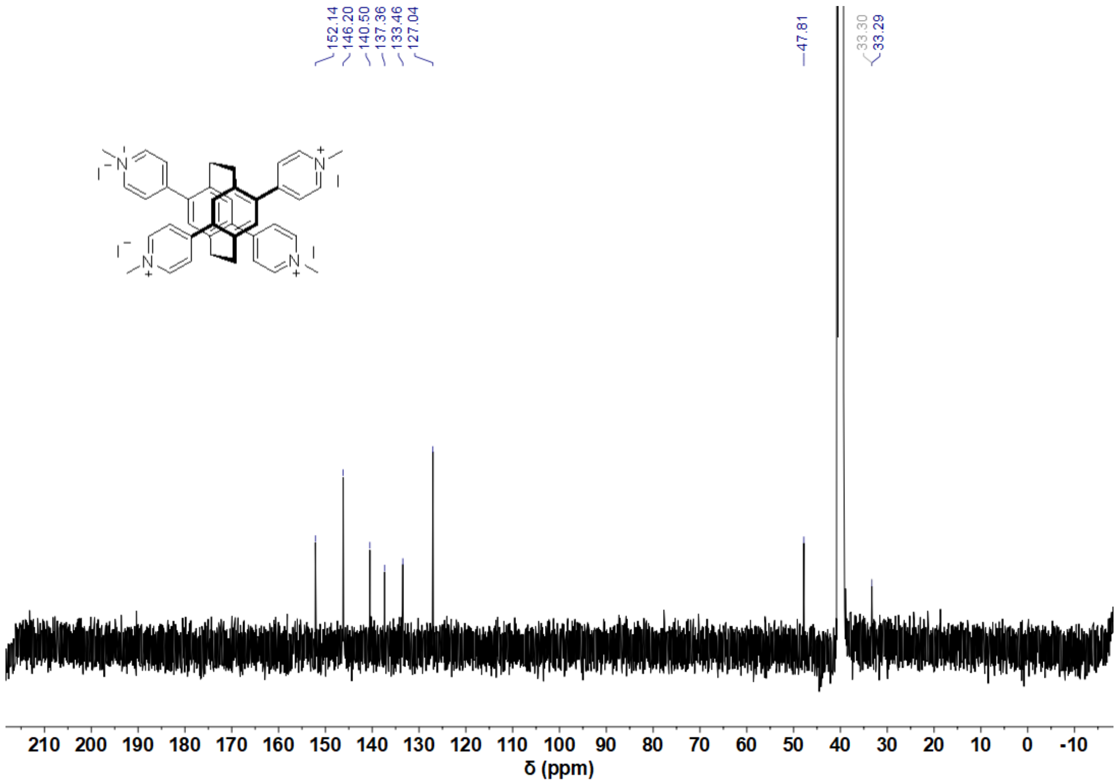

Figure S 16 ^{13}C NMR spectra (400 MHz, $CDCl_3$, r.t.) for **4,7,12,15-tetra(*N*-methyl-4'-pyridinium)[2.2]paracyclophane iodide (QMPCP)**

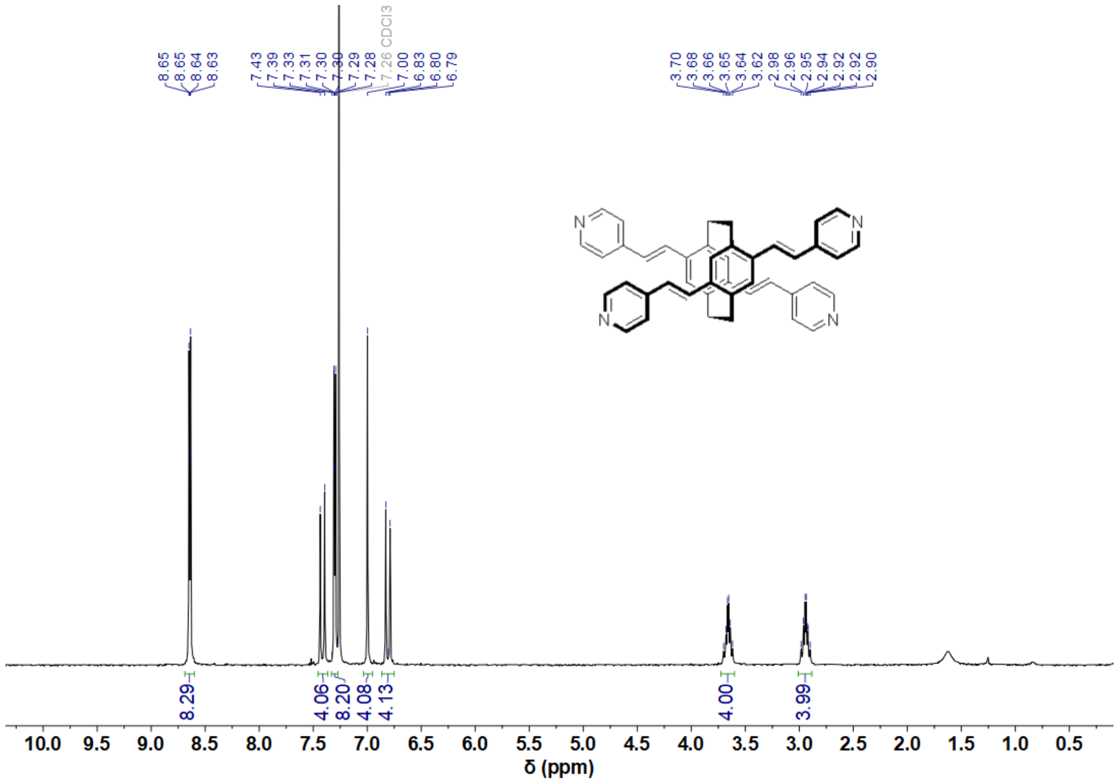

Figure S 17 ^{1}H NMR spectra (400 MHz, $CDCl_3$, r.t.) for **4,7,12,15-tetra(4'-pyridyl-(*E*)-vinyl)[2.2]paracyclophane**

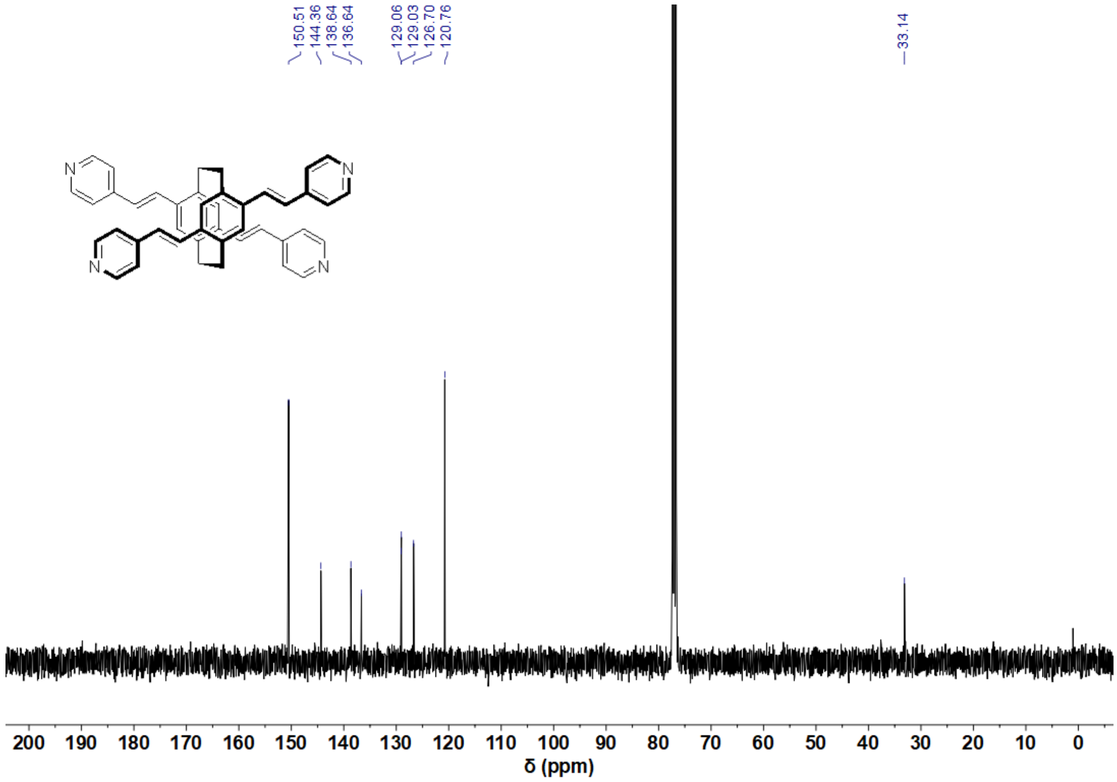

Figure S 18 ^{13}C NMR spectra (400 MHz, $CDCl_3$, r.t.) for **4,7,12,15-tetra(4'-pyridyl-(*E*)-vinyl)[2.2]paracyclophane**

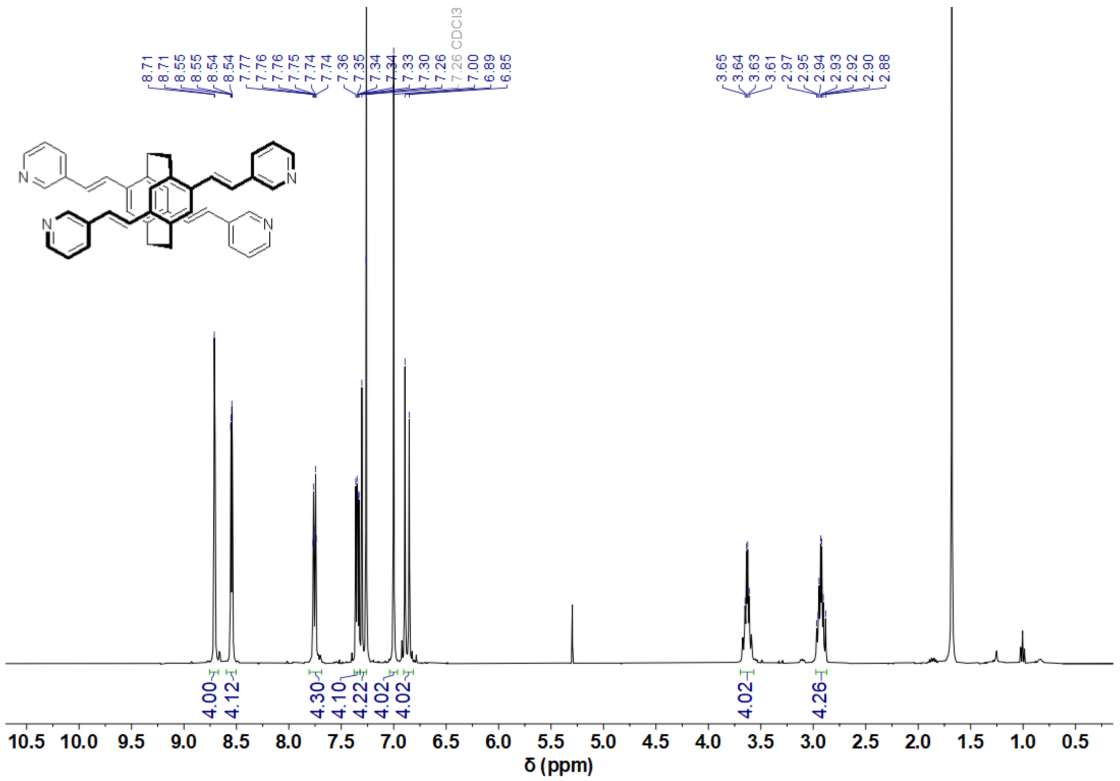

Figure S 19 ^{1}H NMR spectra (400 MHz, $CDCl_3$, r.t.) for **4,7,12,15-tetra(3'-pyridyl-(*E*)-vinyl)[2.2]paracyclophane**

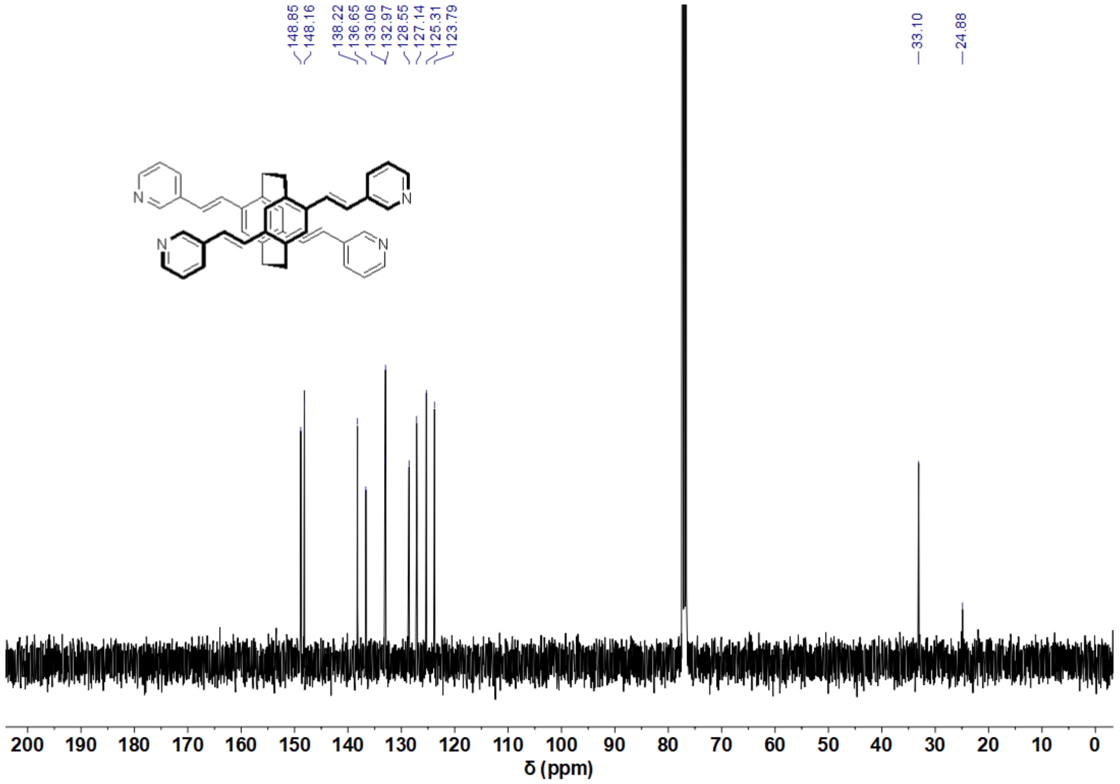

Figure S 20 ^{13}C NMR spectra (400 MHz, $CDCl_3$, r.t.) for **4,7,12,15-tetra(3'-pyridyl-(*E*)-vinyl)[2.2]paracyclophane**

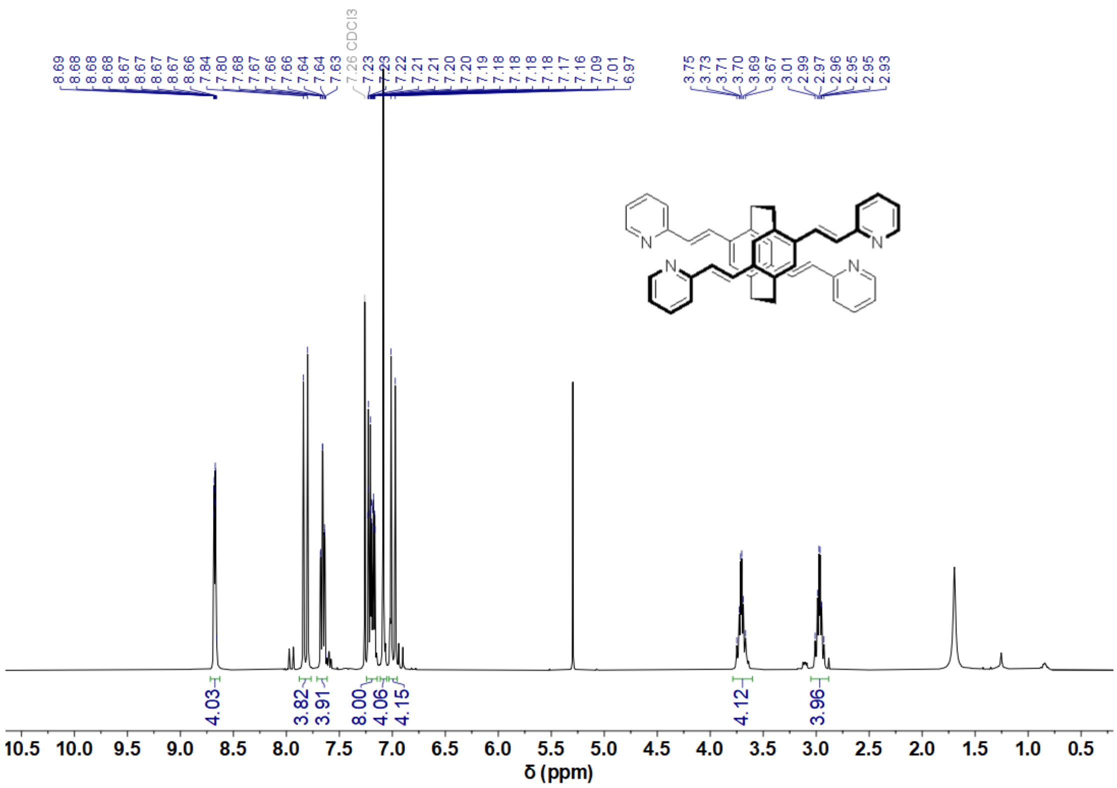

Figure S 21 ^{1}H NMR spectra (400 MHz, $CDCl_3$, r.t.) for **4,7,12,15-tetra(2'-pyridyl-(*E*)-vinyl)[2.2]paracyclophane**

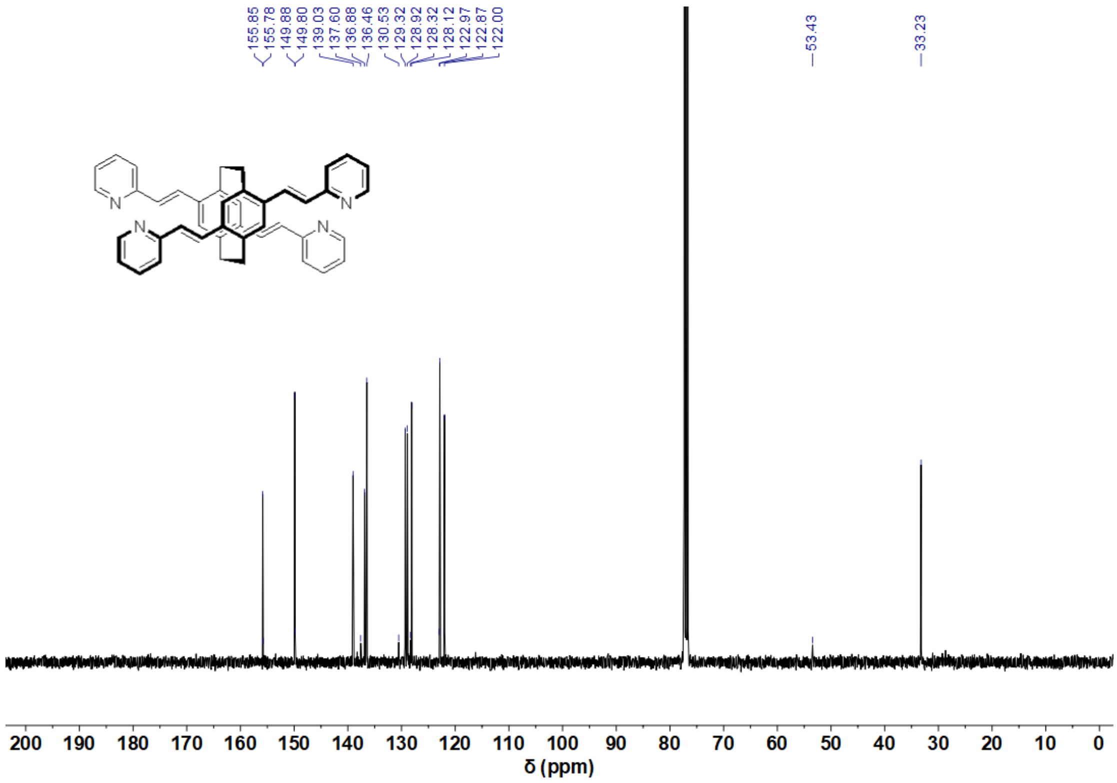

Figure S 22 ^{13}C NMR spectra (400 MHz, $CDCl_3$, r.t.) for **4,7,12,15-tetra(2'-pyridyl-(*E*)-vinyl)[2.2]paracyclophane**

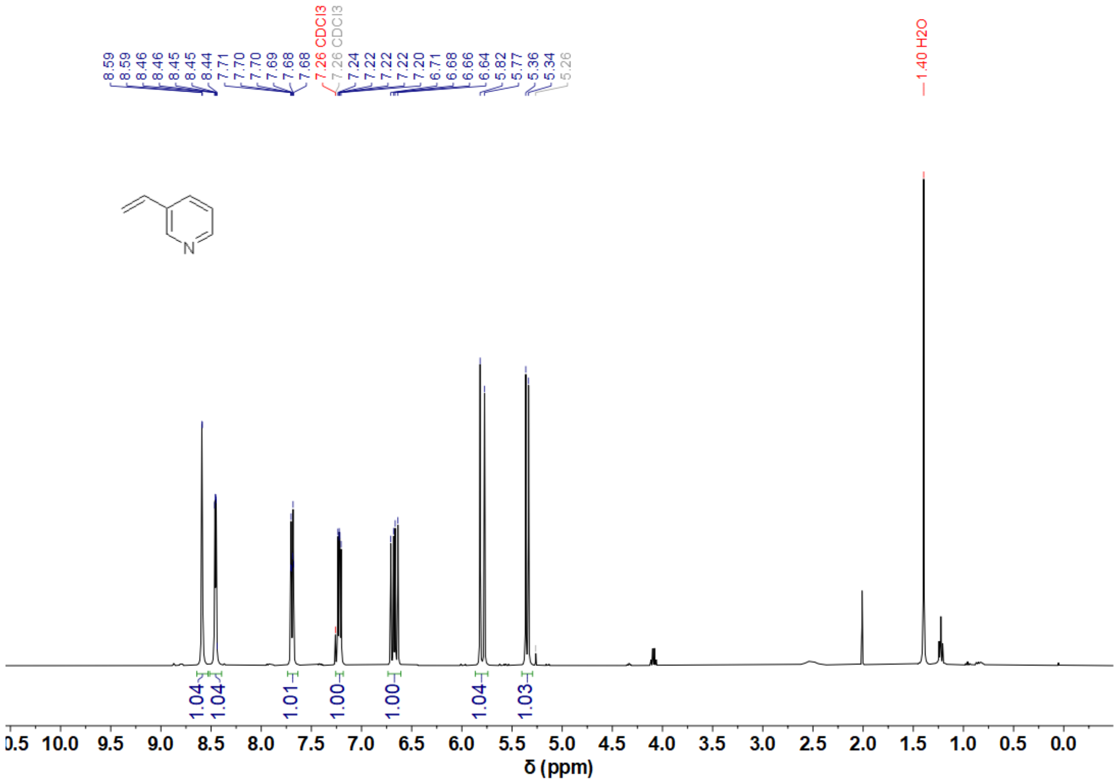

Figure S 23 ^{1}H NMR spectra (400 MHz, $CDCl_3$, r.t.) for **3-vinylpyridine**

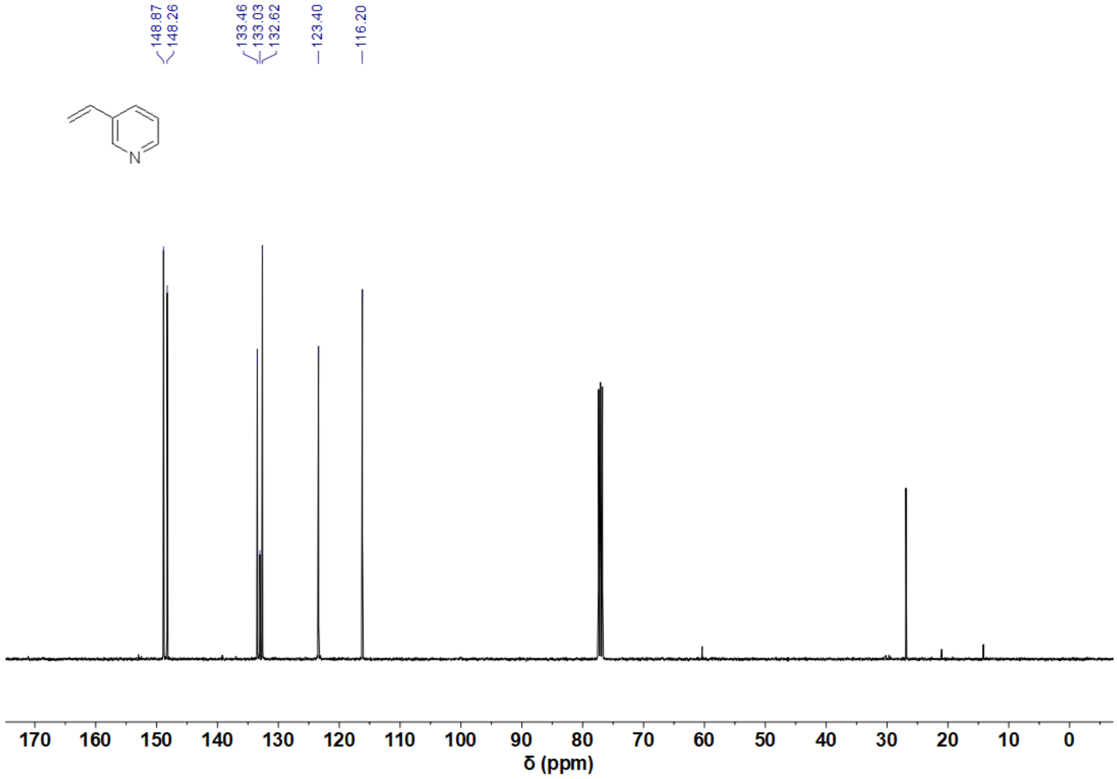

Figure S 24 ^{13}C NMR spectra (400 MHz, $CDCl_3$, r.t.) for **3-vinylpyridine**

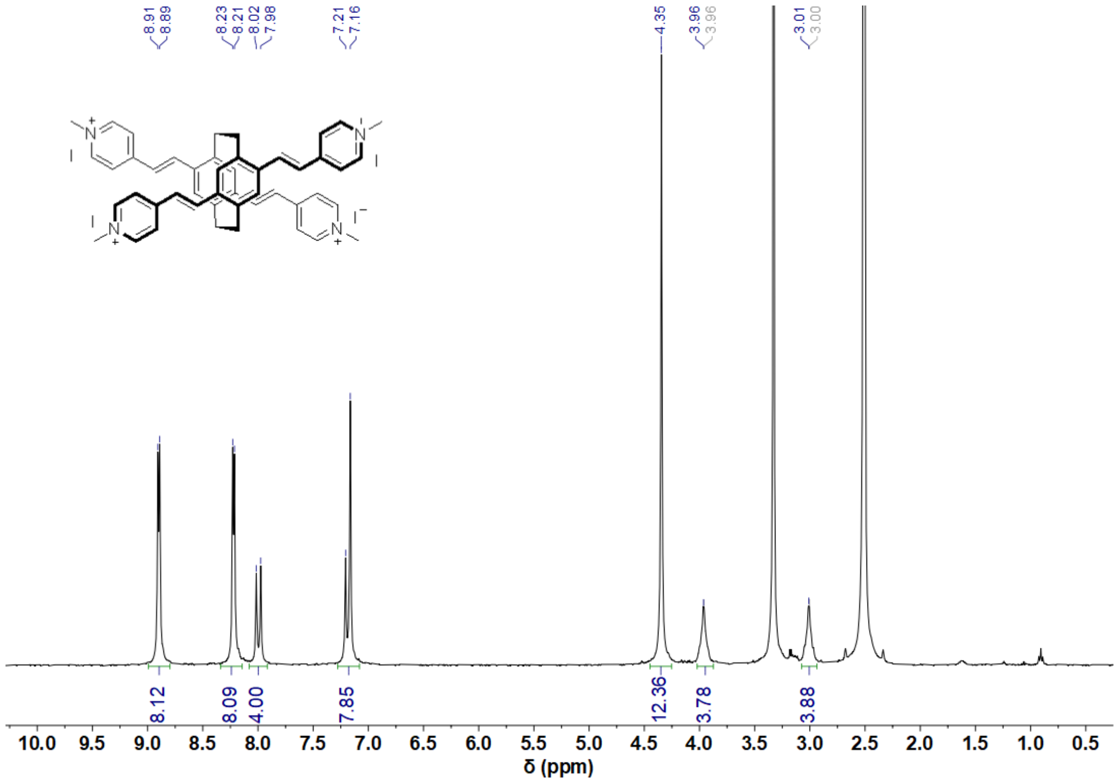

Figure S 25 ^{1}H NMR spectra (400 MHz, $CDCl_3$, r.t.) for **4,7,12,15-tetra(*N*-Methyl-4'-pyridinium-(*E*)-vinyl)[2.2]paracyclophane iodide (QMVCPp)**

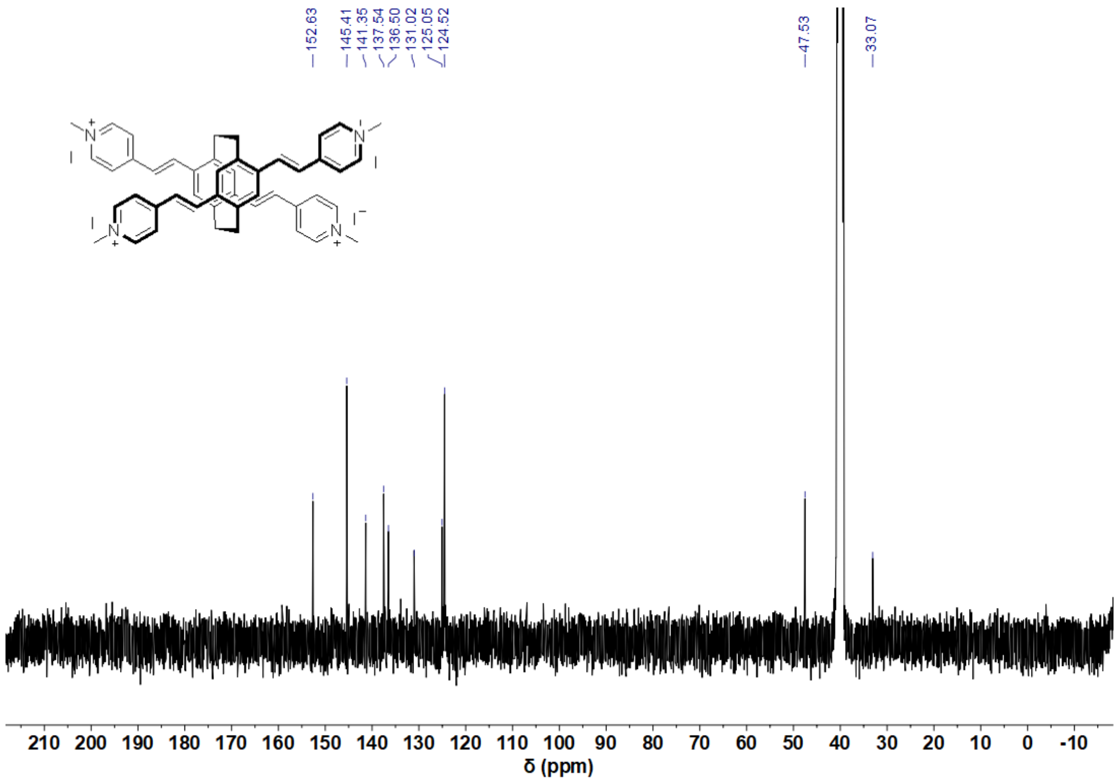

Figure S 26 ^{13}C NMR spectra (400 MHz, $CDCl_3$, r.t.) for **4,7,12,15-tetra(*N*-Methyl-4'-pyridinium-(*E*)-vinyl)[2.2]paracyclophane iodide (QMVCPp)**

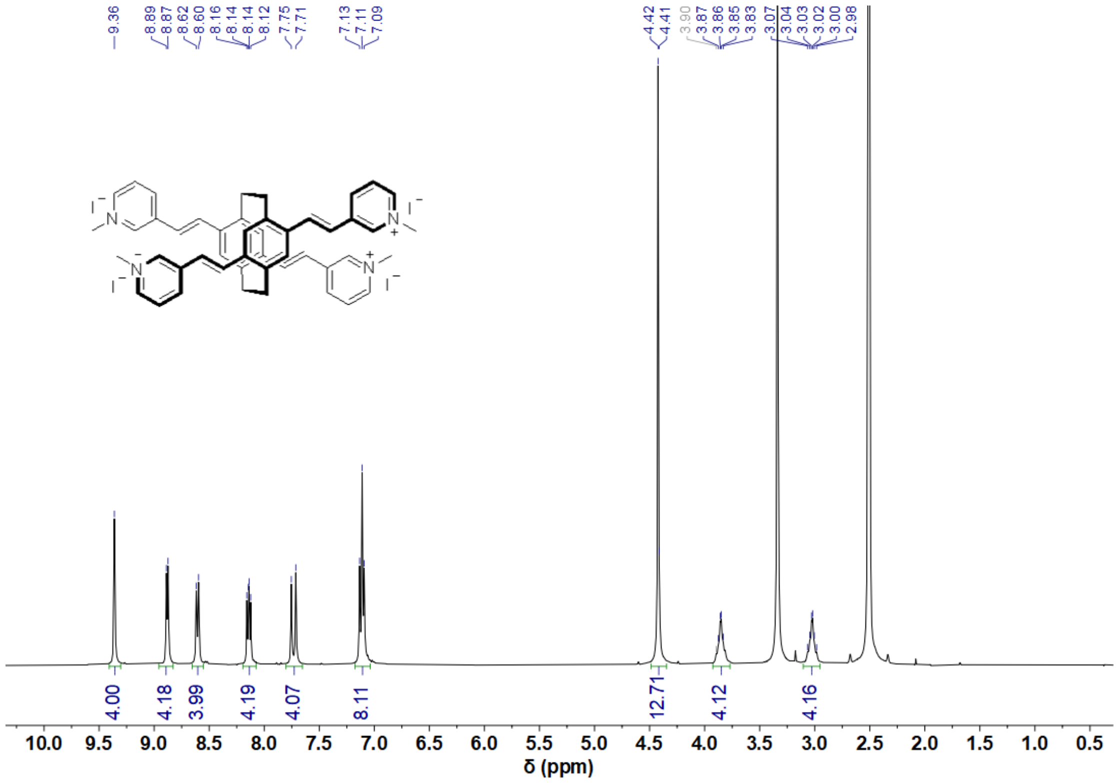

Figure S 27 ^{1}H NMR spectra (400 MHz, $CDCl_3$, r.t.) for **4,7,12,15-tetra(*N*-methyl-3'-pyridinium-(*E*)-vinyl)[2.2]paracyclophane iodide (QMVCPm)**

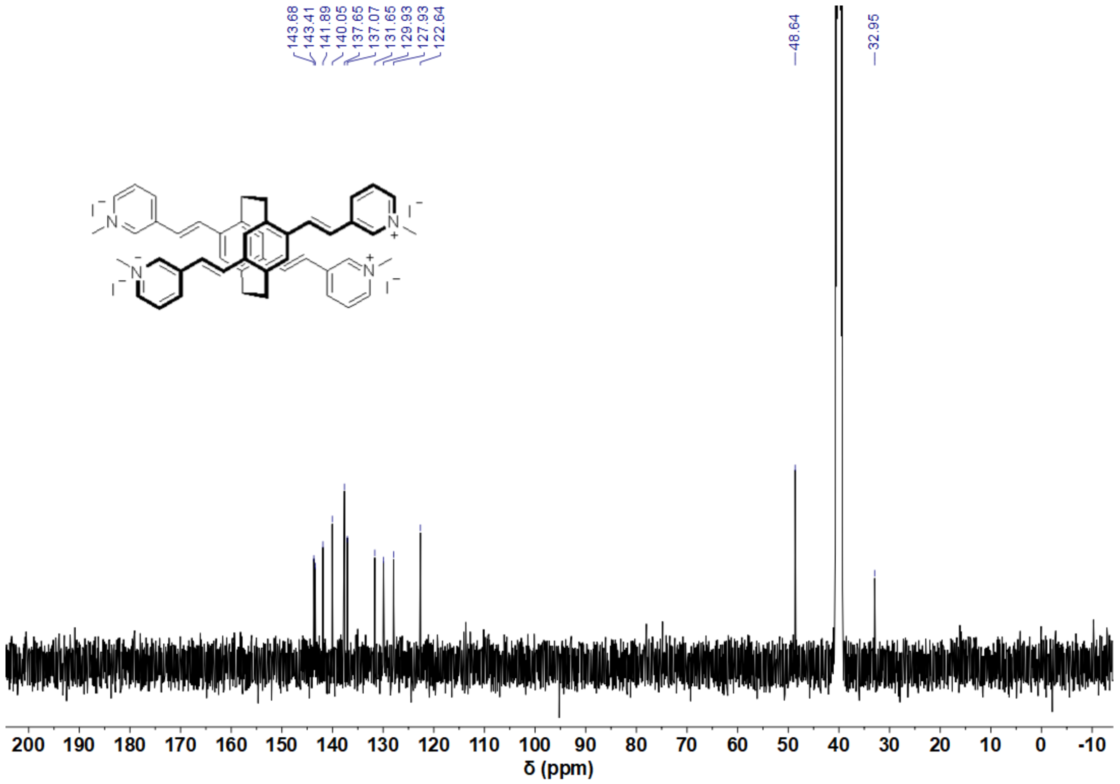

Figure S 28 ^{13}C NMR spectra (400 MHz, $CDCl_3$, r.t.) for **4,7,12,15-tetra(*N*-methyl-3'-pyridinium-(*E*)-vinyl)[2.2]paracyclophane iodide (QMVCPm)**

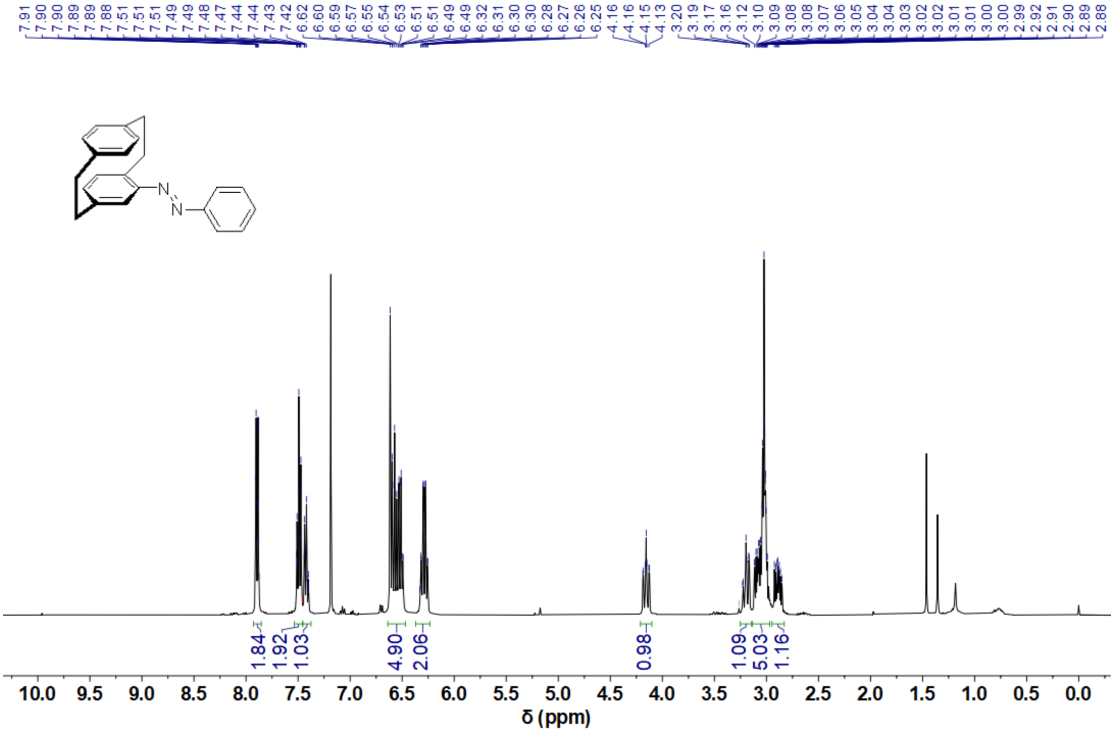

Figure S 29 ^{1}H NMR spectra (400 MHz, $CDCl_3$, r.t.) for **4-(*E*)-Azophenyl[2.2]paracyclophane**

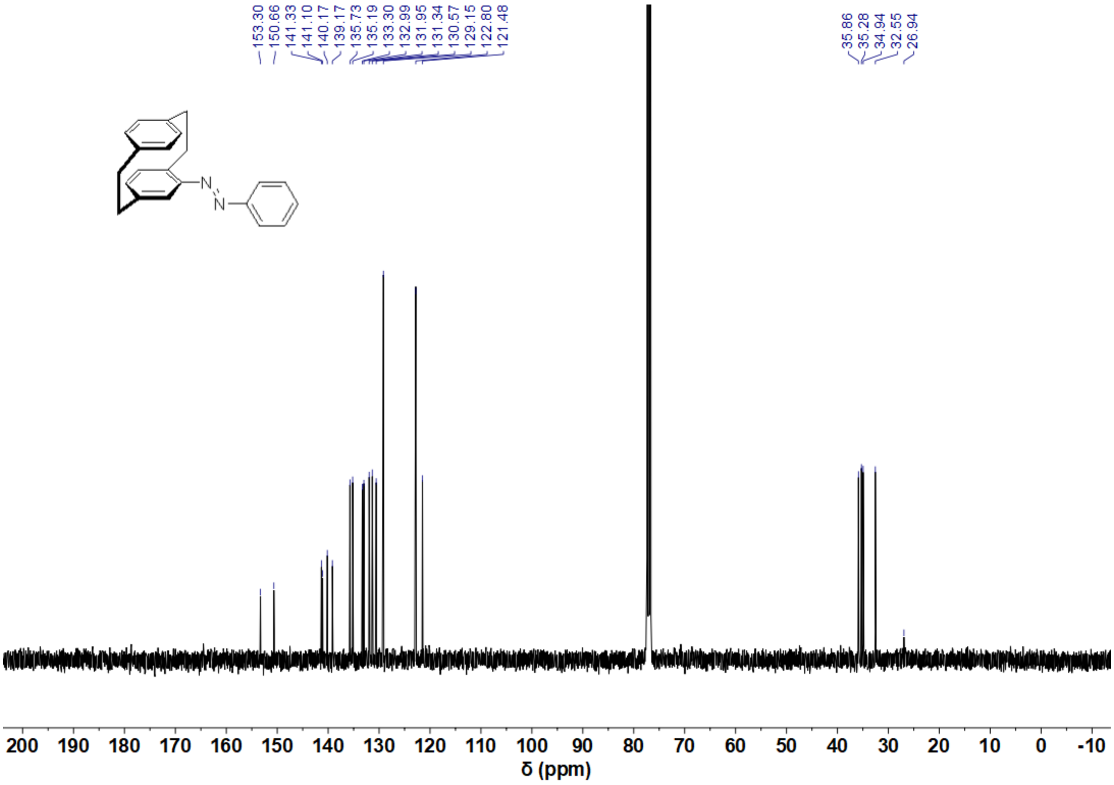

Figure S 30 ^{13}C NMR spectra (400 MHz, $CDCl_3$, r.t.) for **4-(*E*)-Azophenyl[2.2]paracyclophane**

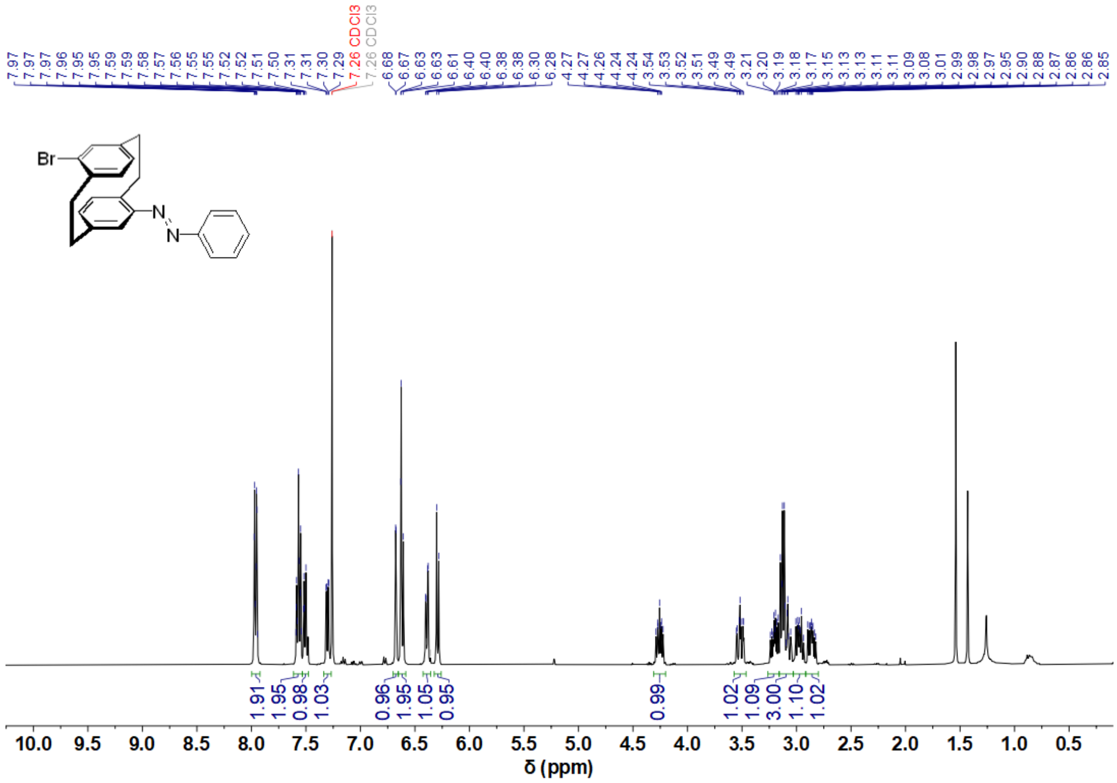

Figure S 31 ^{1}H NMR spectra (400 MHz, $CDCl_3$, r.t.) for **4-(*E*)-Azophenyl-16-bromo[2.2]paracyclophane**

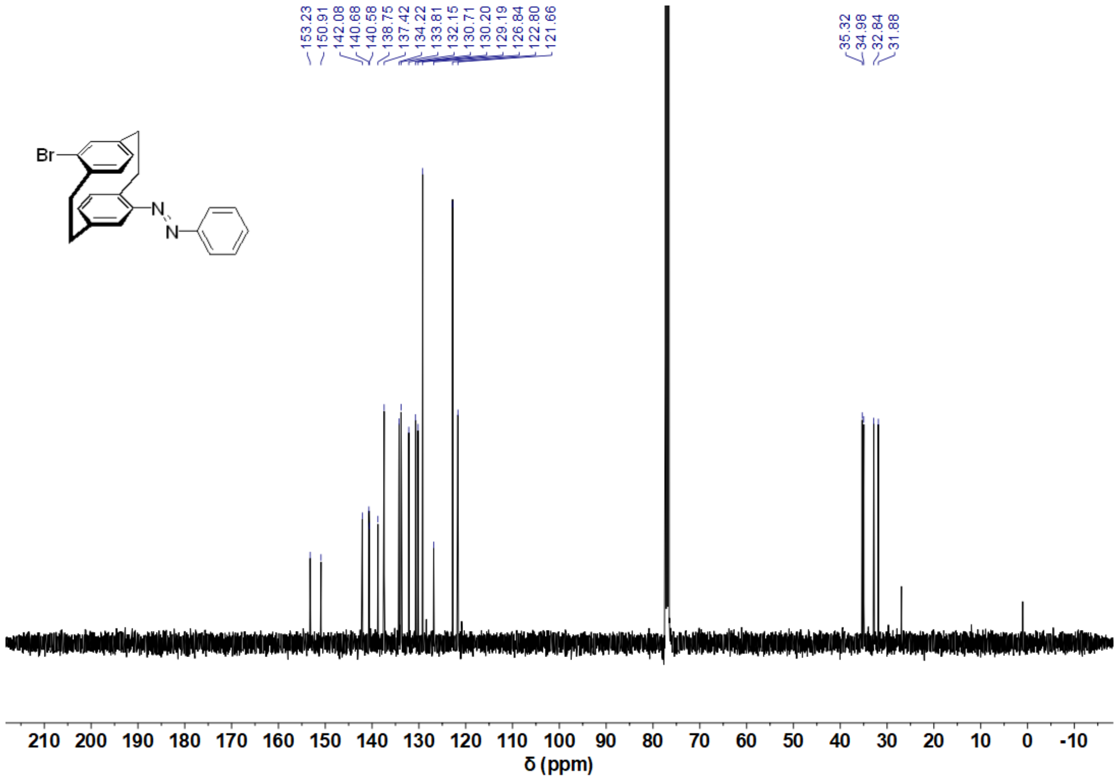

Figure S 32 ^{13}C NMR spectra (400 MHz, $CDCl_3$, r.t.) for **4-(*E*)-Azophenyl-16-bromo[2.2]paracyclophane**

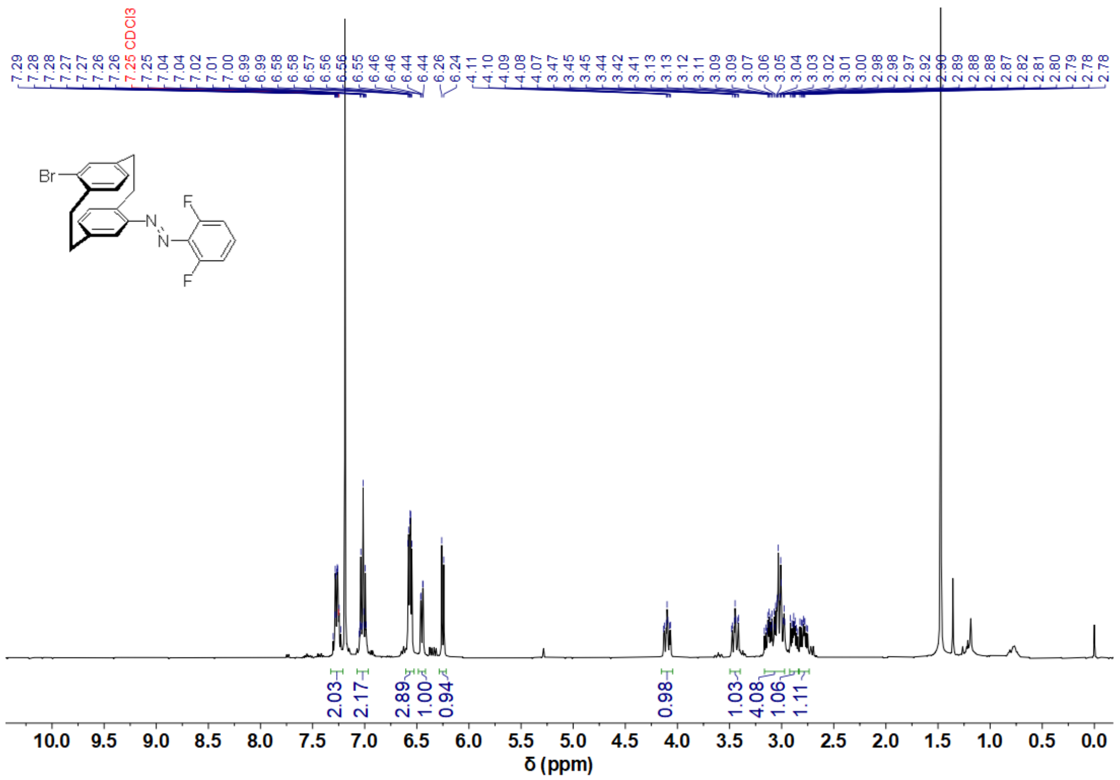

Figure S 33 ^{1}H NMR spectra (400 MHz, $CDCl_3$, r.t.) for **4-((*E*)-Azo-2',6'-difluorophenyl)-16-bromo[2.2]paracyclophane**

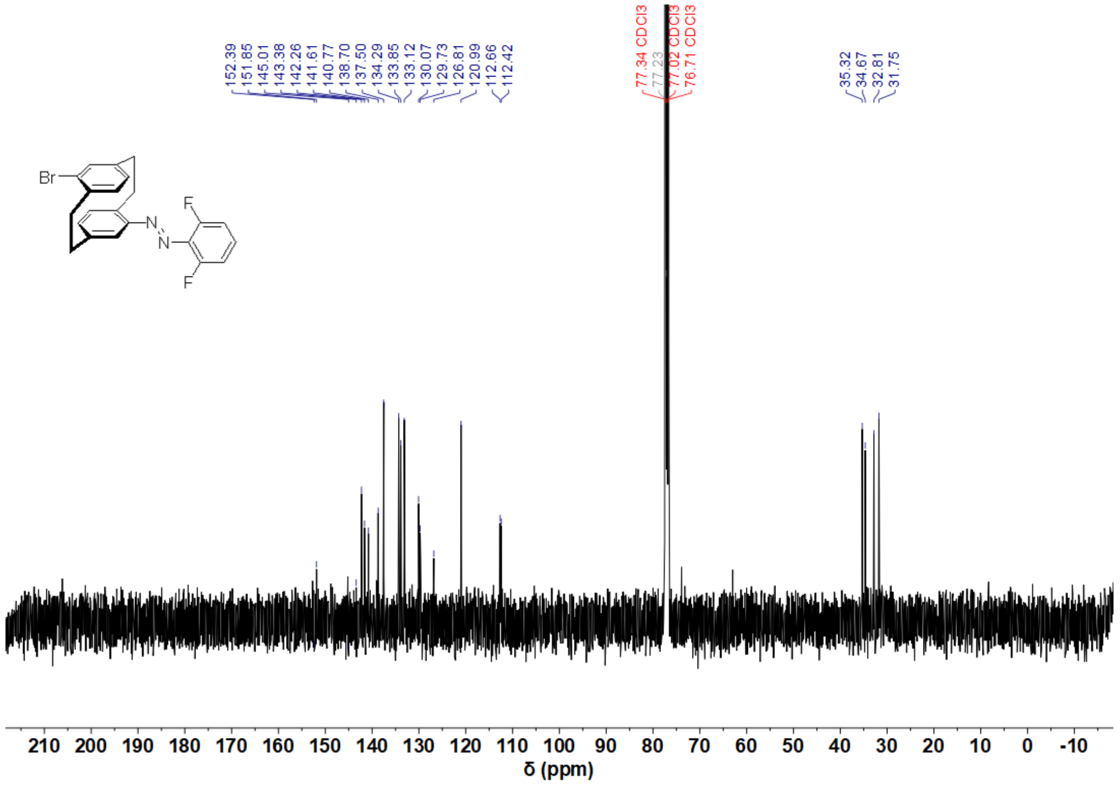

Figure S 34 ^{13}C NMR spectra (400 MHz, $CDCl_3$, r.t.) for **4-((*E*)-Azo-2',6'-difluorophenyl)-16-bromo[2.2]paracyclophane**

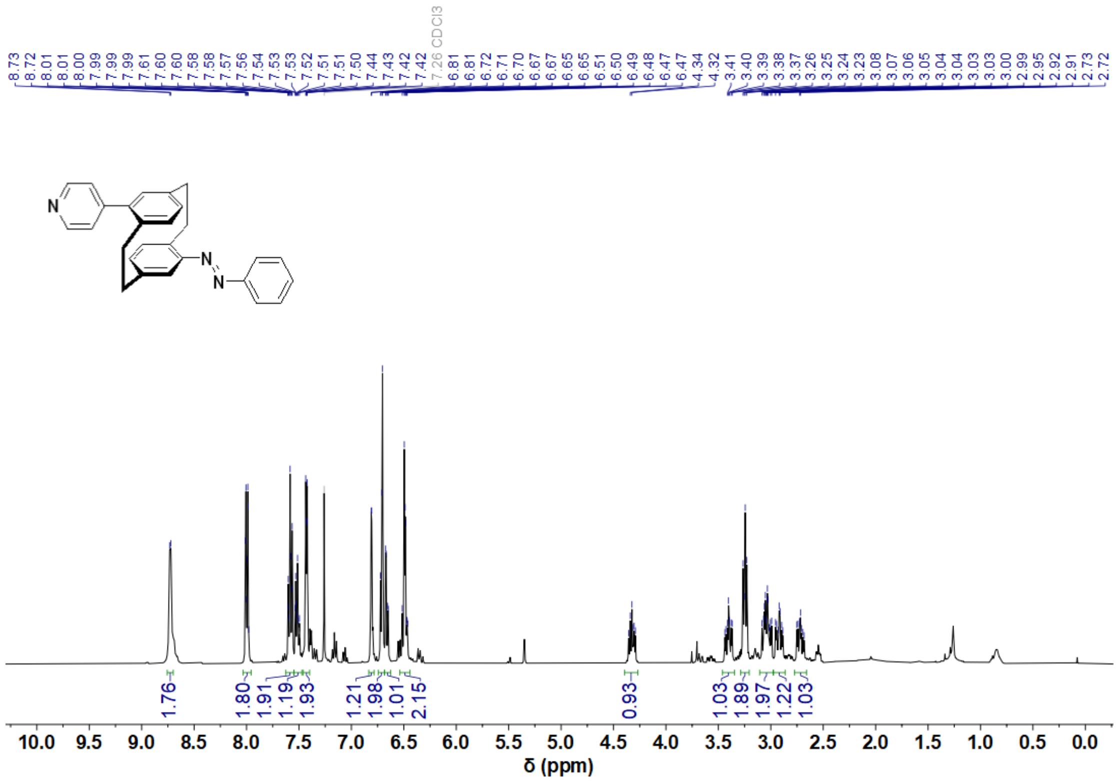

Figure S 35 ^{1}H NMR spectra (400 MHz, $CDCl_3$, r.t.) for **4-(4’-Pyridinyl)-16-(*E*)-azophenyl[2.2]paracyclophane**

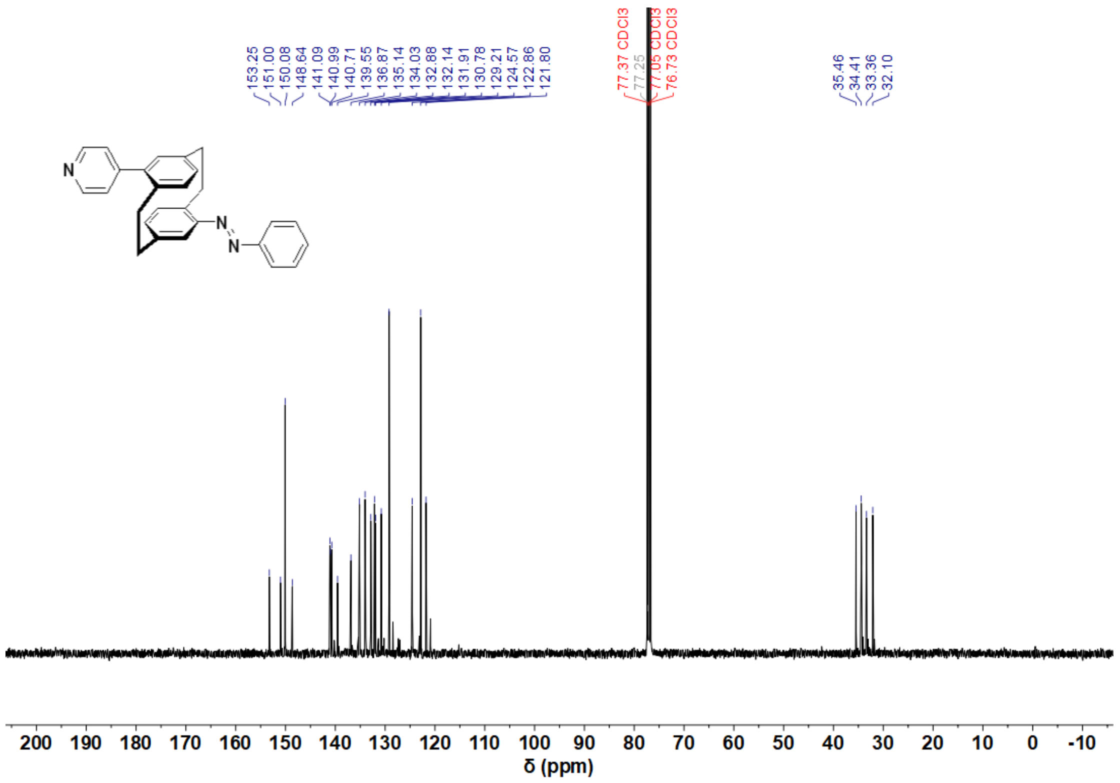

Figure S 36 ^{13}C NMR spectra (400 MHz, $CDCl_3$, r.t.) for **4-(4’-Pyridinyl)-16-(*E*)-azophenyl[2.2]paracyclophane**

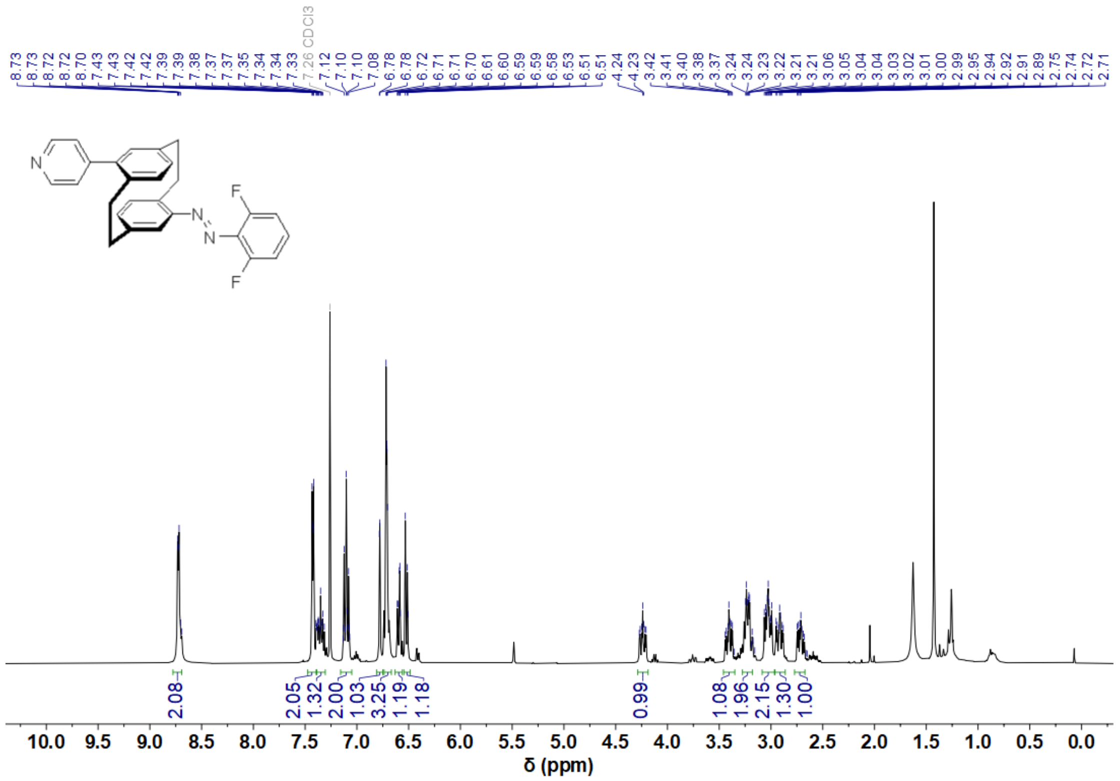

Figure S 37 ^{1}H NMR spectra (400 MHz, $CDCl_3$, r.t.) for **4-(4'-Pyridinyl)-16-((*E*)-azo-2',6'-difluorophenyl)[2.2]paracyclophane**

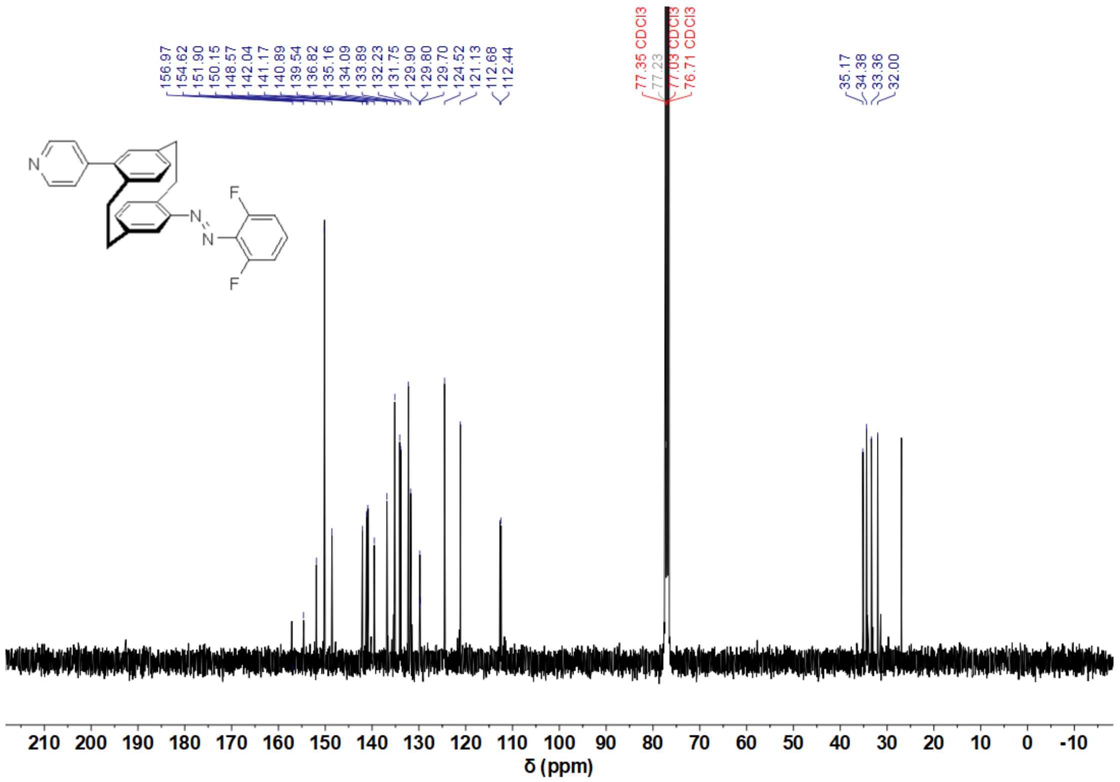

Figure S 38 ^{13}C NMR spectra (400 MHz, $CDCl_3$, r.t.) for **4-(4'-Pyridinyl)-16-((*E*)-azo-2',6'-difluorophenyl)[2.2]paracyclophane**

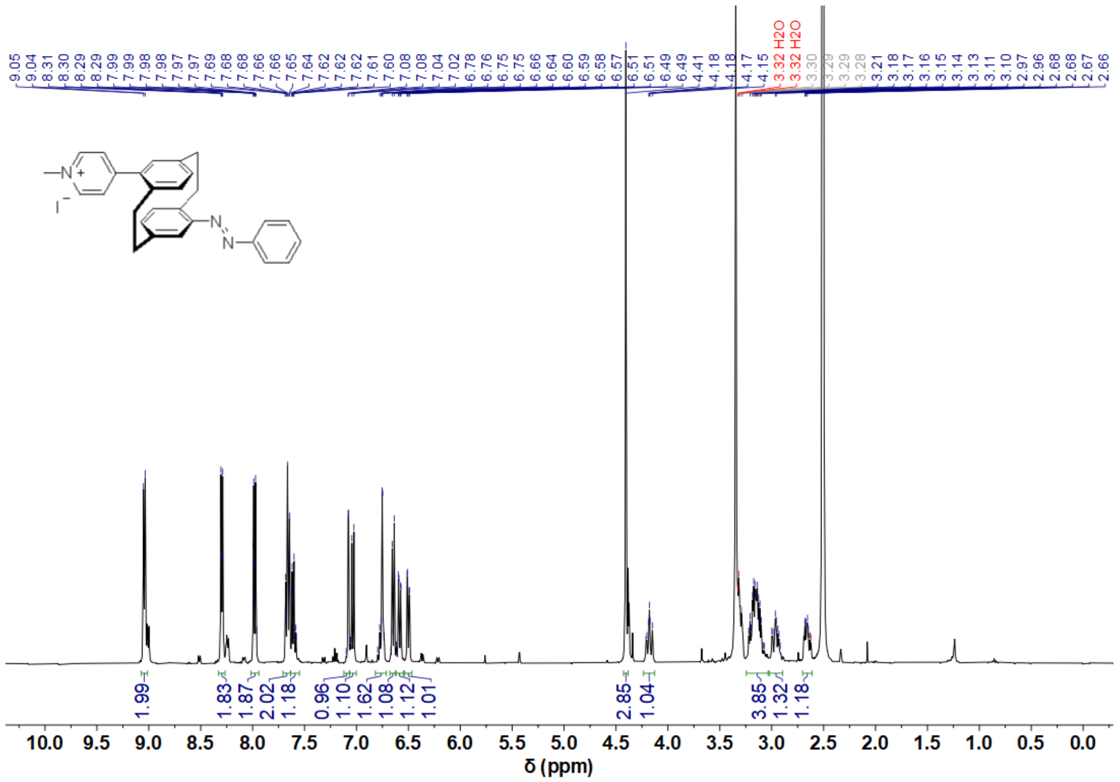

Figure S 39 ¹H NMR spectra (400 MHz, $CDCl_3$, r.t.) for **4-(*N*-Methyl-4'-pyridinium)-16-(*E*)-Azophenyl- [2.2]paracyclophane iodide**

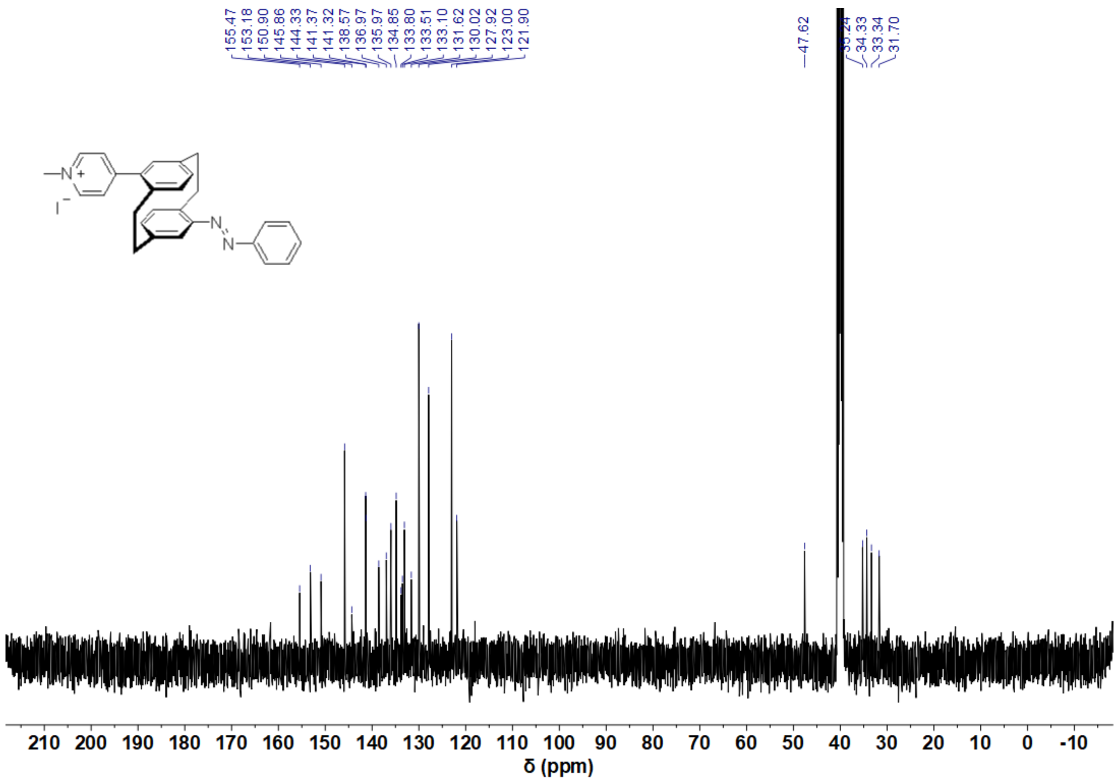

Figure S 40 ¹³C NMR spectra (400 MHz, $CDCl_3$, r.t.) for **4-(*N*-Methyl-4'-pyridinium)-16-(*E*)-Azophenyl- [2.2]paracyclophane iodide**

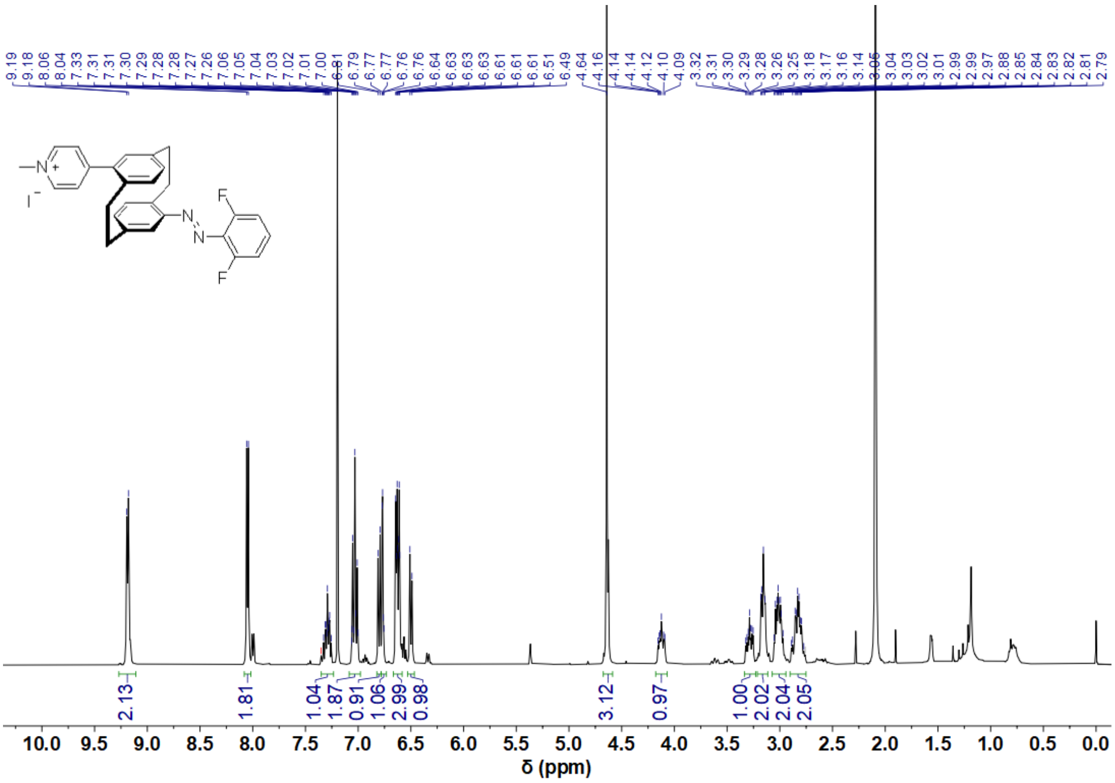

Figure S 41 ^{1}H NMR spectra (400 MHz, $CDCl_3$, r.t.) for **4-(*N*-Methyl-4’-pyridinium)-16-((*E*)-Azo-2’,6’-difluorophenyl)[2.2]paracyclophane iodide**

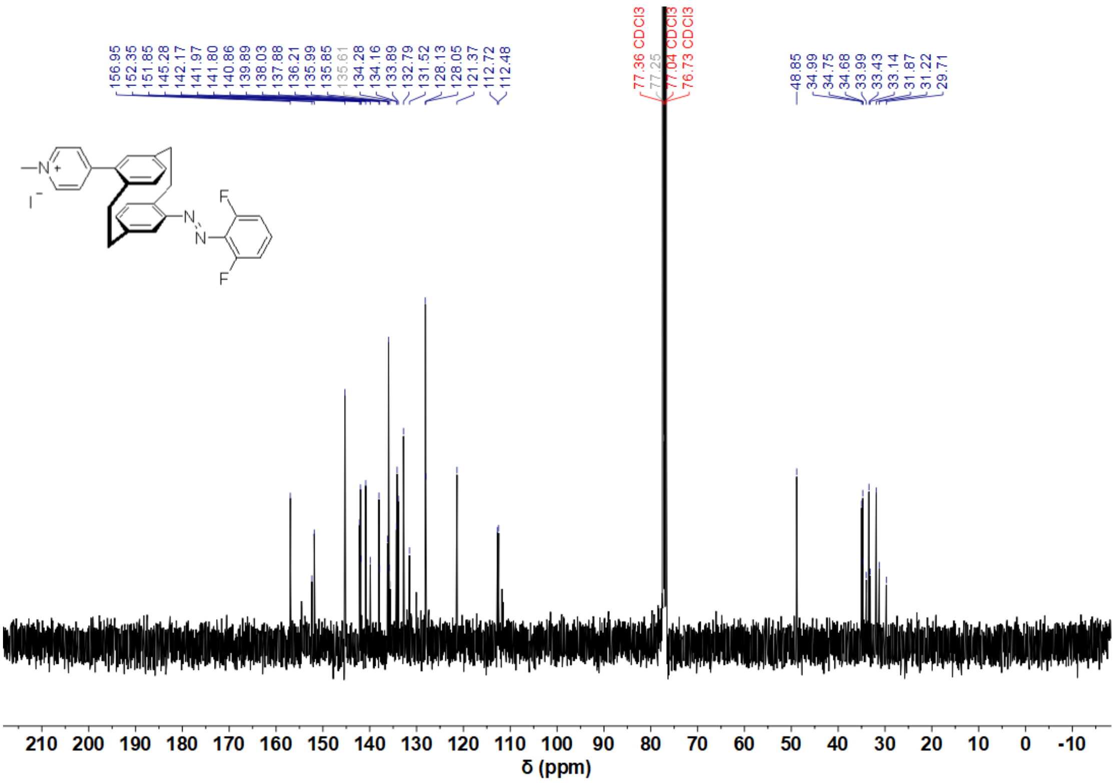

Figure S 42 ^{13}C NMR spectra (400 MHz, $CDCl_3$, r.t.) for **4-(*N*-Methyl-4’-pyridinium)-16-((*E*)-Azo-2’,6’-difluorophenyl)[2.2]paracyclophane iodide**

4 X-Ray Diffractometry

Single crystal X-ray diffraction data of **SBe-P2R1** (CCDC 2240974) and **YCW-103** (CCDC 2240975) were collected on a STOE STADI VARI diffractometer with monochromated Ga Kα (1.34143 Å) radiation at low temperature. Using Olex2 [1], the structures were solved with the ShelXT [2] structure solution program using Intrinsic Phasing and refined with the ShelXL [3] refinement package using Least Squares minimization. Refinement was performed with anisotropic temperature factors for all non-hydrogen atoms; hydrogen atoms were calculated on idealized positions.

[1] O.V. Dolomanov, L.J. Bourhis, R.J. Gildea, J.A.K. Howard, H. Puschmann, *J. Appl. Cryst.* **2009**, *42*, 339–341.
[2] G.M. Sheldrick, *Acta Cryst. A* **2015**, *A71*, 3–8.
[3] G.M. Sheldrick, *Acta Cryst. C* **2015**, *C71*, 3–8.

Crystal Structure Determinations

The single-crystal X-ray diffraction study were of **6** (sb1304_hy YCW-164) and **31** (sb1205_hy_YCW97_sq) carried out on a Bruker D8 Venture diffractometer with PhotonII detector at 123(2) K using Cu-Kα radiation (λ = 1.54178 Å). Dual space methods (SHELXT) [G. M. Sheldrick, *Acta Crystallogr.* 2015, **A71**, 3-8] were used for structure solution and refinement was carried out using SHELXL-2014 (full-matrix least-squares on F^2) [G. M. Sheldrick, *Acta Crystallogr.* 2015, **C71**, 3-8]. Hydrogen atoms refined using a riding model. Semi-empirical absorption corrections were applied.

In **31** (sb1205_hy_YCW97_sq) the iodide and methyl-pyridin-1-ium moiety were disordered. Disordered atoms were refined anisotropically with fixed occupancy (10.52 : 10.48), using restraints. In addition refinement with the listed atoms show residual electron density due to a heavily disordered (unknown, acetonitrile or dichloromethane) solvent molecules which could not be refined with split atoms (2 voids with38 electrons per void). Therefore the option "SQUEEZE" of the program package PLATON (Spek, A.L. (2009). Acta Cryst. D65, 148-155. Spek, A.L. (2015). Acta Cryst. C71, 9-18.) was used to create a hkl file taking into account the residual electron density in the void areas (see attached cif-file for details). Due to the disorder and the squeezed out unknown solvent the data were not deposited into the Cambride database, but could be used for determination of the constitution and confrormation of the compound **31** (SB1255_HY_YCW97).

CCDC Deposition Numbers SB1304_HY CCDC 2241449 (compound **6** in MS X-rays analysis **by M. Nieger**); SB1255_HY_YCW97 (due to the disorder and the squeezed out unknown solvent the data were not deposited into the Cambride database, but could be used for determination of the constitution and confrormation of the compound **31** in MS X-rays analysis **by M. Nieger**).

4,16-Di((*E*)-4’-pyridylvinyl[2.2]paracyclophane (06) (SB1304_HY YCM-164)

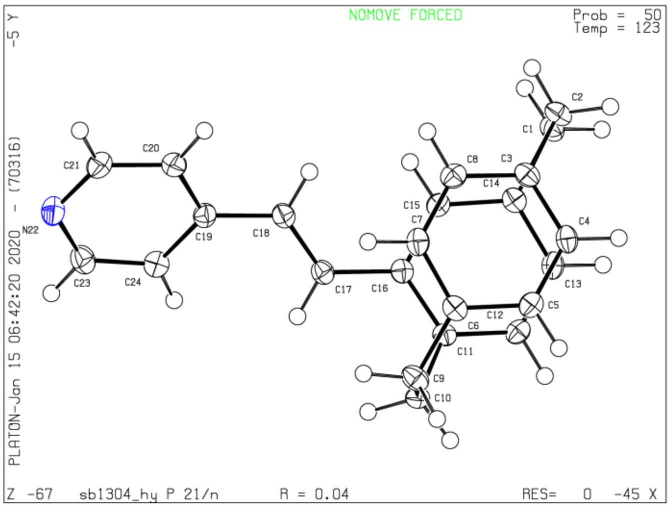

Crystal data

$C_{23}H_{21}N$	*F*(000) = 664
M_r = 311.41	D_x = 1.295 Mg m^{-3}
Monoclinic, *P*2$_1$/*n (no.14)*	Cu *K*□ radiation, □ = 1.54178 Å
a = 9.1926 (8) Å	Cell parameters from 9758 reflections
b = 7.7306 (6) Å	□ = 6.9–72.2°
c = 22.8689 (19) Å	□ = 0.56 mm^{-1}
□ = 100.571 (2)°	*T* = 123 K
V = 1597.6 (2) Å^3	Blocks, colourless
Z = 4	0.36 × 0.24 × 0.12 mm

Data collection

Bruker D8 VENTURE diffractometer with PhotonII CPAD detector	3088 reflections with *I* > 2□(*I*)
Radiation source: INCOATEC microfocus sealed tube	R_{int} = 0.028
rotation in □ and □, 1°, shutterless scans	□$_{max}$ = 72.3°, □$_{min}$ = 3.9°
Absorption correction: multi-scan *SADABS* (Sheldrick, 2014)	*h* = -11□11
T_{min} = 0.838, T_{max} = 0.915	*k* = -9□9
26702 measured reflections	*l* = -28□28
3140 independent reflections	

Refinement

Refinement on F^2	Primary atom site location: dual
Least-squares matrix: full	Secondary atom site location: difference Fourier map
$R[F^2 > 2□(F^2)] = 0.040$	Hydrogen site location: difference Fourier map
$wR(F^2) = 0.103$	H-atom parameters constrained
$S = 1.04$	$w = 1/[□^2(F_o^2) + (0.0464P)^2 + 0.8253P]$ where $P = (F_o^2 + 2F_c^2)/3$
3140 reflections	$(□/□)_{max} = 0.001$
217 parameters	$□□_{max} = 0.26$ e Å^{-3}
0 restraints	$□□_{min} = -0.25$ e Å^{-3}

4,16-Di((*E*)-4’-pyridylvinyl[2.2]paracyclophane (09)_ SBe-P2R1

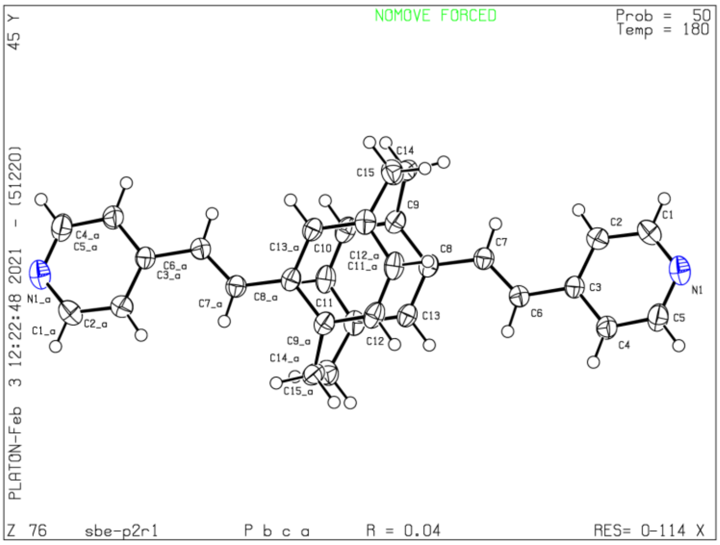

Crystal data and structure refinement for SBe-P2R1.

Identification code	SBe-P2R1
Empirical formula	$C_{30}H_{26}N_2$
Formula weight	414.53
Temperature/K	180.0
Crystal system	orthorhombic
Space group	*Pbca*
a/Å	11.4371(3)
b/Å	7.5642(2)
c/Å	24.9819(9)
α/°	90
β/°	90
γ/°	90
Volume/Å^3	2161.25(11)
Z	4
ρ_{calc}g/cm^3	1.274
μ/mm^{-1}	0.365
F(000)	880.0
Crystal size/mm^3	0.14 × 0.12 × 0.02
Radiation	GaKα (λ = 1.34143)
2Θ range for data collection/°	12.33 to 124.974
Index ranges	-10 ≤ h ≤ 15, -10 ≤ k ≤ 8, -32 ≤ l ≤ 32
Reflections collected	26131
Independent reflections	2420 [R_{int} = 0.0223, R_{sigma} = 0.0079]

Data/restraints/parameters	2420/0/145
Goodness-of-fit on F^2	1.036
Final R indexes [I>=2σ (I)]	R_1 = 0.0411, wR_2 = 0.1087
Final R indexes [all data]	R_1 = 0.0447, wR_2 = 0.1112
Largest diff. peak/hole / e $Å^{-3}$	0.19/-0.18

4,7,12,15-Tetra-(4'-pyridyl-(E)-vinyl)[2.2]paracyclophane (19)_YCW-106

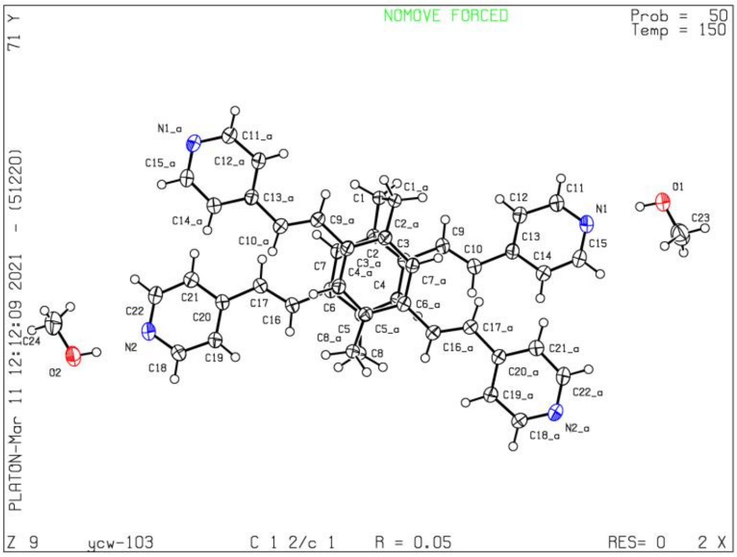

Crystal data and structure refinement for YCW-103.

Identification code	YCW-103
Empirical formula	$C_{48}H_{52}N_4O_4$
Formula weight	748.93
Temperature/K	150.0
Crystal system	monoclinic
Space group	*C*2/*c*
a/Å	13.9463(4)
b/Å	20.2162(7)
c/Å	14.8817(4)
α/°	90
β/°	108.919(2)
γ/°	90
Volume/Å^3	3969.1(2)
Z	4
ρ_{calc}g/cm^3	1.253
μ/mm^{-1}	0.405
F(000)	1600.0
Crystal size/mm^3	0.16 × 0.14 × 0.12
Radiation	GaKα (λ = 1.34143)
2Θ range for data collection/°	6.96 to 124.998
Index ranges	$-15 \leq h \leq 18$, $-24 \leq k \leq 26$, $-19 \leq l \leq 10$
Reflections collected	28257
Independent reflections	4795 [R_{int} = 0.0361, R_{sigma} = 0.0139]

Data/restraints/parameters	4795/0/257
Goodness-of-fit on F^2	1.039
Final R indexes [I>=2σ (I)]	R_1 = 0.0540, wR_2 = 0.1451
Final R indexes [all data]	R_1 = 0.0582, wR_2 = 0.1487
Largest diff. peak/hole / e $Å^{-3}$	0.38/-0.39

(E)-1-methyl-4-(4^3(phenyldiazenyl)-1,4(1,4)dibenzenacyclohexaphane-1^2-yl)pyridin-1-ium iodide (31) – SB1255_HY_YCW97

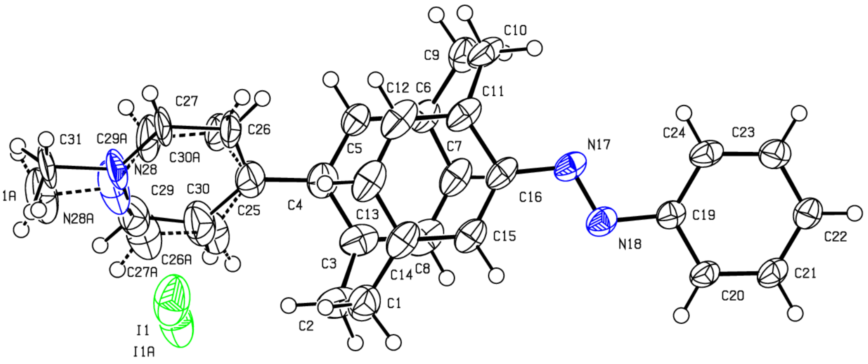

Crystal data

$C_{28}H_{26}IN_3$	*Z* = 8
M_r = 531.42	*F*(000) = 2144
Monoclinic, *C*2/*c (no.15)*	D_x = 1.414 Mg m^{-3}
a = 58.0228 (17) Å	Cu *K*□ radiation, □ = 1.54178 Å
b = 11.6337 (4) Å	□ = 10.22 mm^{-1}
c = 7.4143 (2) Å	*T* = 123 K
□ = 94.203 (2)°	0.06 × 0.04 × 0.02 mm
V = 4991.3 (3) Å^3	

Data collection

25912 measured reflections	$□_{max}$ = 72.1°, $□_{min}$ = 3.1°
4856 independent reflections	*h* = -71□71
3122 reflections with *I* > 2□(*I*)	*k* = -14□14
R_{int} = 0.068	*l* = -8□9
